# Comparative Proteomic Profiling Reveals Molecular Characteristics Associated with Oogenesis and Oocyte Maturation during Ovarian Development of *Bactrocera dorsalis* (Hendel)

**DOI:** 10.3390/ijms18071379

**Published:** 2017-06-30

**Authors:** Dong Wei, Ran Li, Meng-Yi Zhang, Yu-Wei Liu, Zheng Zhang, Guy Smagghe, Jin-Jun Wang

**Affiliations:** 1 Key Laboratory of Entomology and Pest Control Engineering, College of Plant Protection, Southwest University, Chongqing 400716, China; dong_wei1988@yahoo.com (D.W.); liran9610@163.com (R.L.); mengyi_zhang@yeah.net (M.-Y.Z.); yuwei1994@yahoo.com (Y.-W.L.); zhangzheng9523@sina.com (Z.Z.); 2 Academy of Agricultural Sciences, Southwest University, Chongqing 400716, China; 3 Department of Crop Protection, Ghent University, Ghent 9000, Belgium

**Keywords:** oriental fruit fly, isobaric tags for relative and absolute quantitation (iTRAQ), liquid chromatography-tandem mass spectrometry (LC-MS/MS), proteome, ovary, reproduction, oogenesis

## Abstract

Time-dependent expression of proteins in ovary is important to understand oogenesis in insects. Here, we profiled the proteomes of developing ovaries from *Bactrocera dorsalis* (Hendel) to obtain information about ovarian development with particular emphasis on differentially expressed proteins (DEPs) involved in oogenesis. A total of 4838 proteins were identified with an average peptide number of 8.15 and sequence coverage of 20.79%. Quantitative proteomic analysis showed that a total of 612 and 196 proteins were differentially expressed in developing and mature ovaries, respectively. Furthermore, 153, 196 and 59 potential target proteins were highly expressed in early, vitellogenic and mature ovaries and most tested DEPs had the similar trends consistent with the respective transcriptional profiles. These proteins were abundantly expressed in pre-vitellogenic and vitellogenic stages, including tropomyosin, vitellogenin, eukaryotic translation initiation factor, heat shock protein, importin protein, vitelline membrane protein, and chorion protein. Several hormone and signal pathway related proteins were also identified during ovarian development including piRNA, notch, insulin, juvenile, and ecdysone hormone signal pathways. This is the first report of a global ovary proteome of a tephritid fruit fly, and may contribute to understanding the complicate processes of ovarian development and exploring the potentially novel pest control targets.

## 1. Introduction

The oriental fruit fly, *Bactrocera dorsalis* (Hendel), is one of the most devastating agricultural pests worldwide due to its highly reproductive and invasive ability. Several factors that regulate the fecundity and mating behavior of *B. dorsalis* have been previously documented [1,2]. However, due to the long-term and frequent applications of chemical insecticides, *B. dorsalis* has evolved high levels of resistance to many commonly used insecticides [3,4,5]. Insecticide resistance poses a serious threat to current control effort for *B. dorsalis* and other tephritid flies, and there is a great need for novel, environmentally safe, and efficient technologies that are integrated with conventional chemical control methods for sustainable control of *B. dorsalis*. Developing novel pest control strategies can be facilitated by the knowledge of biology and genetics of *B. dorsalis*.

A number of databases with genetic information of *B. dorsalis* are now available such as the transcriptomes of various developmental stages that help identify genes involved in development and reproduction [6], as well as sex-determination [7]. Considerable efforts have also been devoted to creating a male-biased reproductive tissue-specific transcriptome to identify genes involved in spermatogenesis [8]. These genetic information can be served as sources for exploring new molecular targets for pest control; however, it is not always feasible to correlate the transcription levels of mRNAs with the synthesis of the corresponding proteins, and it is impossible to observe post-translational events such as protein modifications from gene expression studies. Therefore, it is necessary to identify proteins in developing testis/ovary directly rather than making inferences about their protein expression from the transcription profile. Thus far, little research has focused on the proteome of *B. dorsalis* for potential target identification. In male accessory glands, 90 proteins that likely regulate female reproduction were identified previously through proteomic approaches [9]. Although such transcriptomes of *B. dorsalis* are available at NCBI, large-scale molecular analysis of reproductive proteins expressed in *B. dorsalis* ovary and their abundance during the ovarian development remains unknown. Understanding the molecular mechanisms of ovary development and oogenesis is essential to manipulate female fertility.

Among the technologies, mass spectrometry has been widely used to analyze proteomes in reproductive tissues, such as testis, male accessory gland, ejaculatory duct, and ejaculatory bulb of *Drosophila melanogaster* [10]. Analysis of ovary proteome is required for a complete understanding of the physiological processes involved in oogenesis and ovary development. Global proteomic characterization of the *D. melanogaster* ovary was recently sequenced and analyzed [11], allowing the discovery of novel regulators and pathways. Such study also provides a systemic view of networks that govern ovarian pathophysiology and embryonic development in flies. In some aspects, *Drosophila* oogenesis represents a valuable developmental platform to genetically and morphologically dissect a wide range of biological processes, such as stem cell self-renewal [12], axis specification [13], cell differentiation and pattern formation [14].

It would be advantageous to identify ovarian proteins since they may have the potential to serve as targets for pest control. Targeting the ovary can be effective because it could affect reproduction by decreasing oogenesis and embryogenesis, thus leading to a reduction in the reproductive rate. Therefore, identification of molecular targets in *B. dorsalis* ovary may aid in the development of novel pest control methods that interfere with female reproduction. In this study, we performed a global proteome analysis of developing ovaries from *B. dorsalis* by using isobaric tags for relative and absolute quantitation (iTRAQ) labeling followed by liquid chromatography-tandem mass spectrometry (LC-MS/MS) with the aim to gather information about ovarian proteins with particular emphasis on differentially expressed proteins (DEPs) during development. The identification of these proteins will increase our understanding of ovarian and oocyte development, and aid in the identification of novel targets for improving tephritid fruit fly control strategies.

## 2. Results and Discussion

### 2.1. Proteome-Wide Identification of Proteins in Ovaries

**Figure 1:**
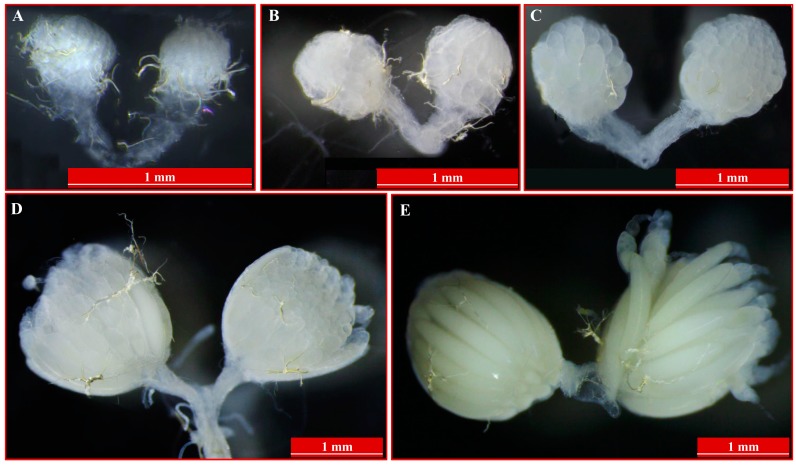
The images of ovaries at different development stages of *Bactrocera dorsalis*. Panels of (**A**–**E**) represent ovaries of 1-, 3-, 5-, 7-, and 9-day-old adults.

**Figure 2:**
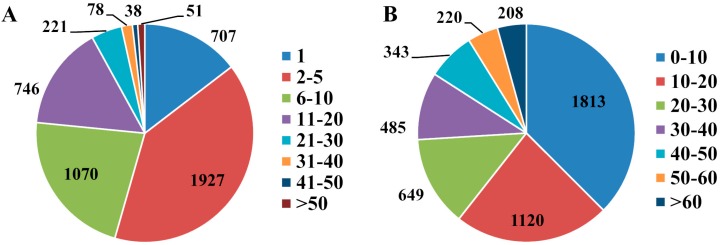
The distribution of peptide number (**A**) and sequence coverage (**B**) of proteins identified in ovaries of *Bactrocera dorsalis* during development.

We observed the ovarian morphology during development at different times (Figure 1), and found that the ovary develops to the vitellogenic stage at 5 days after emergency (Figure 1C). Also, the ovarian size changes significantly during the vitellogenic period. We identified 38,028 peptides, which were assembled to 4838 proteins from 1-, 6-, 9-day-old female ovaries (ov-1, ov-6 and ov-9, respectively) by using LC-MS/MS. Among these, identification of 4053 (83.77%) proteins was based on the identity of more than one peptide. Most of the proteins were identified by 2–20 peptides; 1927 (39.83%) proteins were identified by 2–5 peptides, and 1070 (22.12%) proteins by 6–10 peptides (Figure 2A). About 3.45% of the proteins were identified by more than 30 peptides. The average peptide number of all proteins identified was 8.15. The sequence coverage of specifically identified proteins was estimated as the percentage of matching amino acids between the identified peptides having more than 95% confidence divided by the total number of amino acids in the protein sequence. The sequence coverage of 1813 (37.47%) proteins was less than 10%, and that of 1120 (23.15%) was 10–20% (Figure 2B). The average coverage was 20.79%, in which 15.94% were identified to have more than 40% sequence coverage.

### 2.2. Functional Annotation of Proteins

Because of the accessibility of the *B. dorsalis* genomic information, all identified proteins were transferred to UniProt. Among these identified proteins, 3758, 2401, and 2468 proteins were functionally annotated in Gene ontology (GO) and Cluster of Orthologous Groups (COG) databases, as well as the Kyoto Encyclopedia of Gene and Genomes (KEGG) pathway analysis, respectively. Then, 3758 proteins were categorized into 48 hierarchically structured GO classification, including three ontologies named biological process, cellular component, and molecular function. In the biological process ontology, “cellular progress” and “metabolic progress” were the most dominant categories containing >2000 proteins (Figure S1A). Many proteins involved in reproduction were identified in the biological process and included “reproduction”, “reproductive process”, and “viral reproduction”. In the cellular component ontology, “cell” and “cell part” were the most predominant categories containing >2000 proteins (Figure S1B). However, in the molecular function ontology, the highly represented categories were “binding” and “catalytic activity,” which contained >1600 proteins (Figure S1C). In the COG analysis, 2401 proteins were categorized into 24 COG classifications (Figure S1D). In addition to “general function prediction only,” two categories of “posttranslational modification, protein turnover, chaperones” and “translation, ribosomal structure and biogenesis” were the most dominant categories, while the functions of 90 proteins were unknown. The pathway analysis by KEGG annotation showed that a total of 2468 proteins were mapped to 259 pathways in the KEGG database (Figure S1E). “Metabolic pathways” (687, 27.84%) and “biosynthesis of secondary metabolites” (236, 9.56%) were the largest two categories. There were 72 and 57 proteins assigned to “insulin signaling” and “oocyte meiosis” pathways respectively, that were functionally related with a specialized organ.

### 2.3. Differentially Expressed Proteins

**Figure 3:**
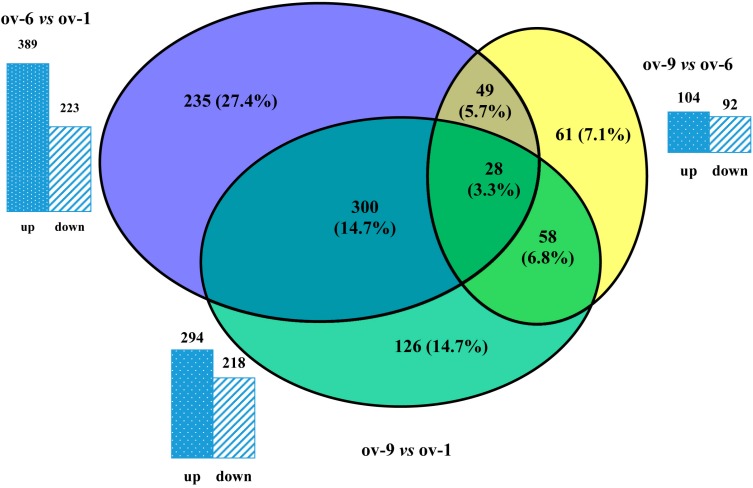
Venn diagrams of differentially expressed proteins between each of the developmental stages during ovary maturation of *Bactrocera dorsalis*. Circles are shown to scale but their overlaps are estimated in each diagram. Up and down mean the protein abundance in the later staged ovaries compared to the early staged ovaries. Number above the bar means the number of protein differentially expressed in two staged ovaries. Ov-1, ov-6 and ov-9 represent ovaries of 1-, 5- and 9-day-old adult of *B. dorsalis*. Different colors of blue, yellow and green represent the protein number of comparisons between ov-6 vs. ov-1, ov-9 vs. ov-6, and ov-9 vs. ov-1, respectively.

In order to analyze the DEPs during the development of ovary, relative quantification of proteins was performed to analyze the abundance of proteins identified in three stages (Figure 3). Briefly, there were 612 DEPs abundant in “ov-1 vs. ov-6” comparison, 389 of which were highly expressed in ov-6 (Table S1), including chitinase-like proteins, heat shock proteins (Hsps), importin (Imp), eukaryotic translation initiation factors (eIFs), NADH dehydrogenases, piRNA pathway proteins, ribosomal proteins, and vitellogenins. Six chitinase-like proteins and three tropomyosins (Tpms) were down-regulated in ov-6. In the quantitative analysis of proteins, 104 and 92 DEPs were highly abundant in ov-9 and ov-6, respectively (Table S2). Three chorion related proteins were highly abundant in the mature ovary, while there were seven ATP-dependent RNA helicases, four eIFs, four ribosomal proteins, and four piRNA pathway proteins that were down-regulated in the mature ovary. Upon comparison with ovary of 1-day-old adult, 294 out of 512 DEPs were highly expressed in ov-9 (Table S3). Most of these DEPs (328) were also identified in the first quantitative comparison.

**Figure 4:**
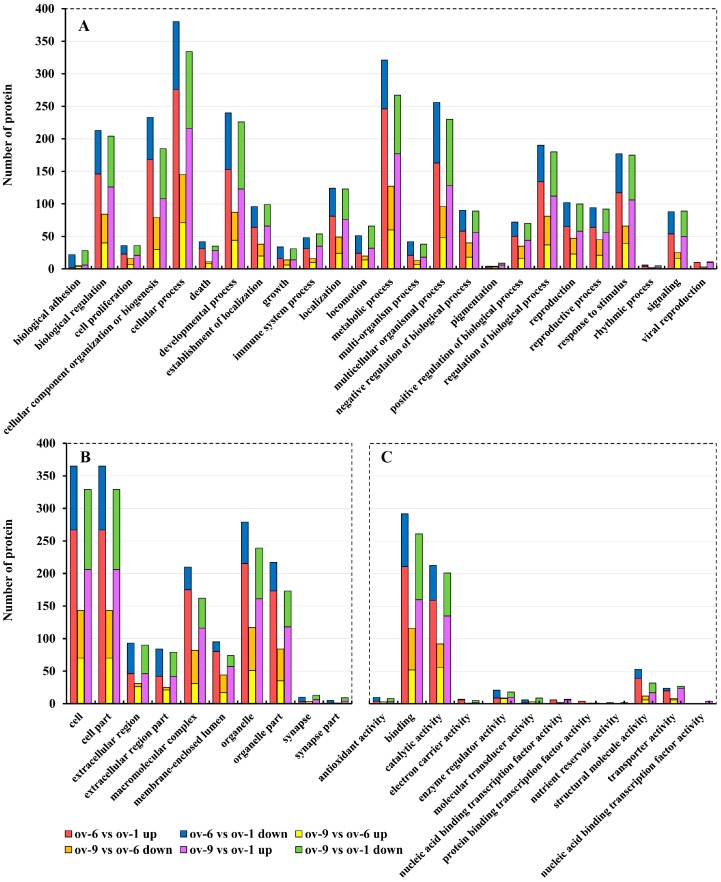
Gene ontology (GO) analysis of differentially expressed proteins in three comparisons by iTRAQ analysis. Shown above is the classification of these proteins in different categories based on biological process (**A**), cellular component (**B**), and molecular function (**C**). Ov-1, ov-6 and ov-9 represent ovaries of 1-, 6- and 9-day-old adult of *B. dorsalis*.

**Figure 5:**
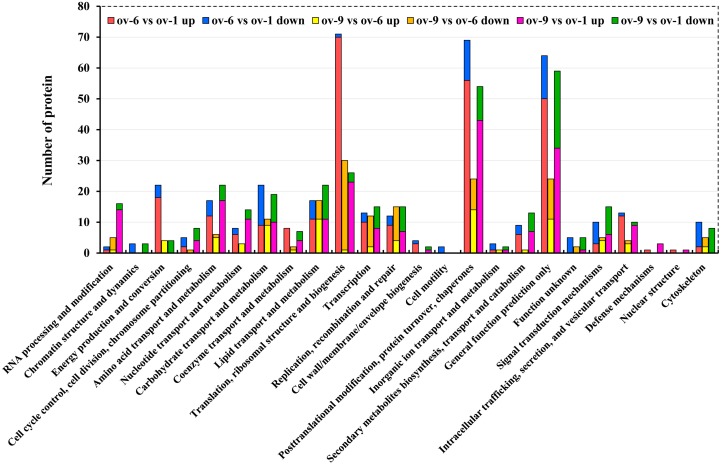
Clusters of Orthologous Groups of protein classification of differentially expressed proteins in three comparisons by iTRAQ analysis. Ov-1, ov-6 and ov-9 represent ovaries of 1-, 6- and 9-day-old adult of *B. dorsalis*.

All the DEPs between each comparison were functionally annotated as above. A total of 672 DEPs were functionally annotated into three ontologies by GO analysis (Figure 4), among which 419, 170, and 407 DEPs were involved in the biological process. The highly expressed categories were “cellular process”, “metabolic process”, and “multicellular organismal process” (Figure 4A). There were 249 and 231 DEPs involved in “reproduction” and “reproductive process,” respectively. Moreover, “cell”, “cell part” and “organelle” were the predominant categories in cellular component ontology (Figure 4B). In the molecular function category, most DEPs were assigned to “binding” and “catalytic activity” (Figure 4C). A total of 450 DEPs from each comparison were aligned to 24 categories after COG annotation (Figure 5). Most of the up-regulated DEPs in “ov-6 vs. ov-1” comparison were involved in “translation, ribosomal stricture and biogenesis” (70 DEPs), “posttranslational modification, protein turnover, chaperones” (56 DEPs), “energy production and conversion” (18 DEPs) and “general function prediction only” (50 DEPs). In the identification analysis, the largest category was “general functional prediction only” as mentioned above, indicating strong biogenesis and metabolism towards protein synthesis in ov-6. Twenty-nine DEPs involved in “translation, ribosomal stricture and biogenesis” were down-regulated in the mature ovary implying their roles specific to the vitellogenic stage.

### 2.4. DEPs Highly Abundant Ovary of 1-Day-Old Adult

**Figure 6:**
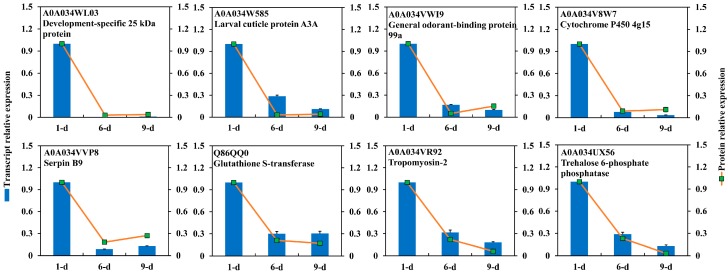
Transcriptional expression of proteins highly abundant in early ovary of 1-day-old *Bactrocera dorsalis* adult. Gene expression in three time-point stages of ovary, 1-day, 6-day and 9-day, were determined by quantitative PCR. All relative expression was compared to that in ovary of 1-day-old adult.

A total of 153 DEPs were identified as highly abundant in ov-1 compared to later stages (Table 1), including tropomyosins (Tpms), myosin regulatory light chain, chitinase-like protein, cytochrome P450 (P450 4g15), development-specific protein, general odorant-binding protein (GOBP99a), glutathione *S*-transferase (GSTo1), and larval cuticle protein. Eight of these DEPs were validated at the transcriptional level by qRT-PCR. All eight genes showed high expression in 1-day-old ovary, which is consistent with the protein abundance (Figure 6). The high expression at both transcriptional and protein levels implied their crucial roles in the initial development of *B. dorsalis* ovary.

Tpms are actin-binding cytoskeletal proteins, which play vital roles in various cellular processes including cytokinesis, cell migration, embryogenesis, and oocyte maturation [15,16]. Several Tpms isoforms were found to be expressed in specific developmental patterns, correlating with the differentiation of embryonic stem cells and response to polarizing signals at early embryonic development in the mouse ovary [15,17]. Tpm3 localizes in the cortex before metaphase II of the mouse oocyte, showing a role in asymmetric cell division and maintenance of cortical integrity [16]. In *Drosophila*, five Tpms are identified, and Tpm2 plays a role in *oskar* RNA localization in the posterior pole of oocytes resulting in the development of the abdomen and germ line [18]. Lack of Tpm2 affects head morphogenesis leading to death at the first instar stage [19]. In this study, three Tpms were identified, namely Tpm1 (A0A034VND6, A0A034VPE5) and Tpm2 (A0A034VR92), which showed high abundance in ov-1. The transcriptional expression of Tpm2 showed a consistent pattern with the proteomic data, indicating a similar role in oogenesis and ovarian development in *B. dorsalis*.

Dramatic change in the localization of cytoplasmic myosin (non-muscle) is a characteristic feature of early embryogenesis in *D. melanogaster* [20]. Maternal supply of myosin II is required for cytoplasmic transport during oogenesis [21]. Germ line cystoblasts lacking a functional light chain myosin II show severe defects in proliferation and cytokinesis. For instance, the cytoplasmic bridges linking the oocyte to the nurse cells in the egg chamber are abnormal during oogenesis [22,23]. In addition, numerous myosin heavy chain accumulate in the light chain deficient cells. Similar to *Drosophila*, a non-muscle light chain myosin was identified as abundant in ov-1. The essential role of myosin II for rapid cytoplasmic transport during oogenesis was also investigated [21]. A myosin V is confirmed to regulate *oskar* mRNA localization in the *Drosophila* oocyte [24], and an unconventional myosin VI, encoded by *myosin heavy chain at 95F*, is required for follicle cell epithelial development during egg chamber morphogenesis [25]. Both homologous myosin V (A0A034V675) and myosin VI (A0A034V7N4) were also identified to be highly abundant in ov-6.

There were many proteins that were highly abundant in the initial stage of *B. dorsalis* ovary including GOBP, GSTs, P450, development-specific protein, notch-like protein, serine/threonine protein kinase and its inhibitor (Serpin), and metalloproteases. It has been reported that chemosensory proteins are abundantly expressed in the female reproductive organ [26], although their function in reproduction has been studied mostly in male insects [27]. Formation of notch signaling regulation, maintenance of the germ stem cell niche, and the role of cap cells in determining the niche size in the *Drosophila* ovary has been well studied [28,29]. GSTs expressed in the reproductive organ have been identified in testis germ cells of male rats, and their activity increased after exposure to oxidative stress [30]. Transcriptional expression showed that one GST (Q86QQ0) was highly expressed in the early stage of ovary. Additionally, another detoxifying enzyme, P450, was also identified to be abundant in ov-1. The specific functions of these DEPs should be determined prior to their use for pest control. In addition, a development-specific protein (A0A034WL03), and SerpinB9 (A0A034VVP8) were abundantly present in ov-1, indicating their stage specific function in oogenesis. Interestingly, four larval cuticle proteins and three chitinase-like proteins were identified as abundant in ov-1, but their functions in ovarian development are not known. Twenty seven uncharacterized proteins were identified during the initial stage of ovary. These proteins likely play roles in cell differentiation during early ovarian development. A large number of proteins (48%) in newly formed ovaries were also not functionally identified in *Metapenaeus ensis* [31]. Further studies should focus on the early development of insect ovary.

### 2.5. DEPs Highly Abundant in Ovary of 6-Day-Old Adult

**Figure 7:**
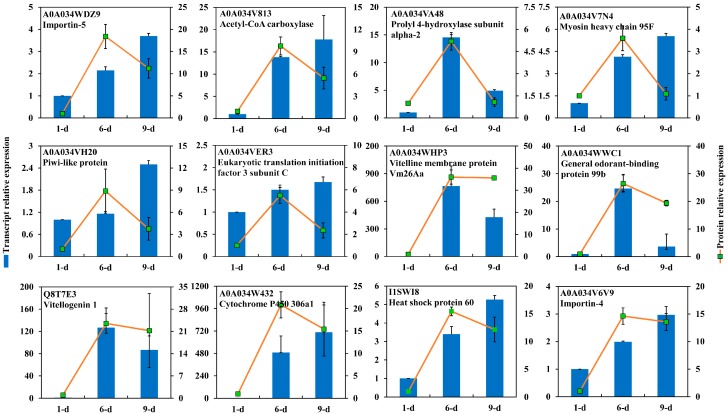
Transcriptional expression of proteins highly abundant in ovary of 6-day-old *Bactrocera dorsalis* adult. Gene expression was calculated as Figure 6.

Among the DEPs, 389 were up-regulated during intermediate stages of ovary. Of these, 44 were down-regulated at mature ovary (Table 2), including eIFs, Imps, ribosomal proteins, villin-like protein, as well as piRNA pathway related proteins. Additionally, 152 proteins highly abundant in ov-6 were also found to be highly expressed in ov-9 (Table S4), such as vitelline membrane protein (Vm26Aa), vitellogenin (Vg1), GOBP99b, P450 306a1, Hsps (Hsp70, Hsp75, Hsp60, and Hsp23), 26S proteasome, and GST, etc. Transcriptional expressions of tested proteins differed from the protein expression (Figure 7). Three out of six DEPs abundant in ov-6 were highly expressed in the mature ovary with only one DEP (prolyl 4-hydroxylase α2) highly expressed in ov-6 at transcriptional levels. Two out of six DEPs abundant in both vitellogenic and mature ovaries were highly expressed in ov-6, namely Vm26Aa and GOBP99b; two DEPs were highly expressed in mature ovary at the transcriptional level; and two DEPs had the same expression patterns.

In *Drosophila*, there are three Piwi proteins termed Aubergine, Piwi, and Argonaute-3 in distinct piRNA pathways with different functions in ovarian germ and somatic cells development [32]. Two interrelated branches of the piRNA system have been reported: somatic cells that support oogenesis only employ Piwi, whereas germ cells utilize a more elaborate pathway centered on the three gonad-specific Argonaute proteins [32,33]. Piwi protein regulates both niche and intrinsic mechanisms to maintain germline stem cells during oogenesis in *Drosophila* [34]. Also, Tudor protein in *Drosophila* is a component of two types of germ granules: nuage, which is assembled during ovary development; and polar granule, which forms at the posterior cytoplasm of the oocyte and is maintained in early embryo [35]. A novel role was recently reported for Tudor domains in the Tudor–Aubergine protein complex assembly and distribution during *Drosophila* oogenesis [36]. It has also been demonstrated that some of the Piwi proteins are necessary for very early stages of oogenesis within the germarium [37]. The complex regulation of piRNA pathway on oogenesis is clear in *Drosophila*, showing a potential use in pest control by regulating reproduction. A piwi-like protein and two tudor-domain-containing proteins were identified to be highly expressed in ov-6 likely due to their regulation in oocyte maturation.

In this study, seven eIFs were identified to be highly abundant in the late stage, indicating their potential roles in ovarian development. Generally, translational repression of mRNAs in the eggs of various insects have been confirmed by interactions, either direct or via intermediate proteins, of repressive factors bound to the 3′-UTRs of proteins in the eIF4E family bound to the 5′-cap of transcripts [38]. In mouse oocyte, a oocyte-specific eIF4E is highly expressed in fully grown oocytes [39]. Insects also rely on the regulated translation of select maternal mRNAs to control oocyte maturation and the initial stages of embryogenesis. These transcripts usually remain silent until their translations are temporally and spatially required during early development. Increasing evidence suggests that eIF4E interacts with cell-specific molecules to control translation during oogenesis and early development in insects [40]. In *Drosophila*, the Cup protein directly associated with eIF4E is known to be crucial for diverse aspects of female germ-line development [41]. A reduction in eIF4E activity deteriorates the development of ovaries [42]. Only one of these seven eIFs (eIF3C) were transcriptionally validated by qRT-PCR and was shown to have a pattern that differed from protein profiles in the proteomics data.

In the present study, four Hsps were identified at low amounts in newly emerged ovaries, indicating their likely function in oogenesis during ovary maturation in *B. dorsalis*. A follicle cell specific Hsp83 was identified in mature ovaries of *Tribolium castaneum* [43]. Moreover, Hsp83 has been reported to function as a component of cap-binding complex and to interact with eIF4E in regulating oogenesis at early stages of egg chamber development during oogenesis in *Drosophila* [44]. Hsps in the reproductive organs were also reported in testis of *B. dorsalis*, such as Hsp70s and Hsp90s [8]. In addition, a small Hsp (sHsp27) was identified as highly expressed in germline nurse cells throughout oocyte development at the late stages of oogenesis in *Drosophila* [45]. Stage and cell-specific expression of sHsp27 was recently identified to be differentially expressed and to be located in the ovary during oogenesis of *Ceratitis capitata* [46]. In *B. mori*, six sHsps were identified to be highly expressed in the ovary [47]. In this study, one Hsp (Hsp60, I1SWI8) was evaluated at the transcriptional level, which showed increased abundance during ovary development (Figure 7).

The intracellular localization of the 26S proteasome in the different ovarian cell types of *D. melanogaster* has been well-studied. During the pre-vitellogenic phase of oogenesis in *Drosophila*, cytoplasmic 26S proteasome is observed in the nurse cells and follicular epithelial cells. However, a characteristic subcellular redistribution occurs in the ovarian cells during the vitellogenic phase of oogenesis, indicating a strictly tissue- and developmental stage-specific distribution [48]. Here, we identified four 26S proteasome non-ATPase regulatory subunits that were highly abundant in the vitellogenic stages. Their high accumulation in the oocyte was also reported in *D. melanogaster* [49]. However, their mechanism of regulation in the oocyte remains unknown. It was not surprising that Vg was identified as highly abundant in ov-6 and ov-9 ovaries. In insects, Vg is synthesized in the fat body in a process that involves substantial structural modifications of the nascent protein prior to its secretion and transport to the ovary [50]. It has been reported that Vgs of *B. dorsalis* are also expressed in the ovary, indicating a complementary Vg function in ovarian development [51]. In this study, a Vg was identified as abundant in the late ovary, as well as the Vg receptor (S4TMC4).

### 2.6. DEPs Highly Abundant in Ovary of 9-Day-Old Adult

**Figure 8:**
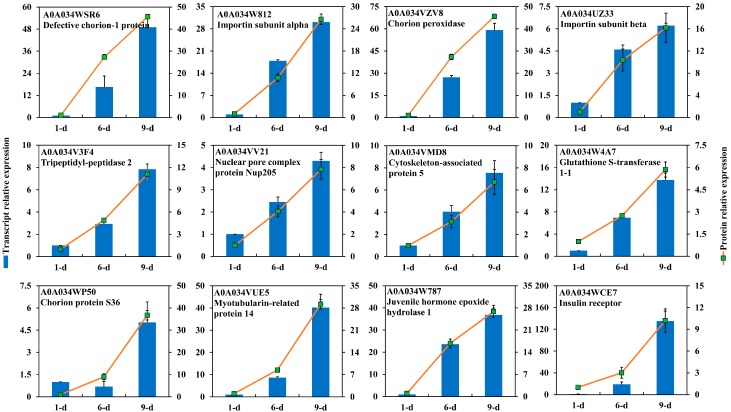
Transcriptional expression of proteins highly abundant in ovary of 9-day-old *Bactrocera dorsalis* adult. Gene expression was calculated as Figure 6.

A total of 59 DEPs were highly abundant in the mature ovary (Table 3), among which 17 DEPs were increasingly abundant during the ovarian development. These included defective chorion-1 protein, chorion peroxidase, Impα, Impβ, nuclear pore complex protein (Nup205), tripeptidyl-peptidase 2, and GST. Other proteins were also identified as highly abundant in mature ovary and include chorion protein S36, myotubularin-related protein, juvenile hormone epoxide hydrolase (JHEH1), and insulin receptor. Most of these DEPs may function in oocyte maturation. Six DEPs showed a similar increasing gene expression pattern along with an increase in the protein levels, while only one DEP (chorion protein s36) out of six DEPs abundant in ov-9 showed a consistent transcriptional expression specifically in ov-9. The remaining five of the tested proteins had increasing expression patterns (Figure 8).

Impβ was originally described to participate in the import of proteins that carry a classical nuclear localization signal (NLS) into the nucleus, a key player in nuclear protein import [52]. Nurse cells synthesize and transfer Impβ into the oocyte cytoplasm from late stage of oogenesis in *D. melanogaster*, and Impβ gene appears to be ubiquitously expressed in embryonic cells [53]. Impβ interacts with the nuclear pore complex (NPC), NLS dependent protein and also Impα forming a trimeric complex. Thus, Impα plays a role in oogenesis in association with Impβ. It has been demonstrated that the concentration of Impα3 within the nurse cell nuclei increases during stages 7–10 (vitellogenic stage) of oogenesis, and plays a crucial role in the completion of oogenesis [54]. Impα2 protein is uniformly distributed in the nurse cell cytoplasm with a moderate accumulation along the oocyte cortex [55]. Indeed, there are three Impα with a specific and limited expression profiles in *D. melanogaster* spermatogenesis, but they play different roles in oogenesis [56]. Homologous Impα and Impβ were both identified as the most abundant in the mature ovaries of *B. dorsalis*. Consistent transcriptional expression indicated their crucial roles in oocyte maturation. The Impα defect in female *Drosophila* causes sterility and leads to the arrest of oogenesis in late stage [54,56,57]. The roles of Imps in the development of both larval and adult tissues were also uncovered, suggesting a potential for their use in pest control. However, their functions and mechanisms of regulation during oogenesis should be well addressed in *B. dorsalis*. In addition, four NPCs were also identified as abundant in ov-6 while another NPC was more abundant in ov-9. Besides, we also identified three Imp (Imp4, -5, -7) that were highly expressed during the vitellogenic development. Imp7 is distantly related to the proteins of Impβ family, and is required for the proper formation of muscle-tendon adhesion sites in developing *Drosophila* embryos [58]. The high expressions of Imp4 and Imp5 were also determined by qRT-PCR, indicating their important roles in oocyte development (Figure 7).

The eggshell, including Vmps and chorion proteins, is a specialized extracellular matrix that is synthesized between the oocyte and overlaying somatic follicle cells during the late stages of oogenesis. As follicles mature, they move through the ovarioles and undergo successively vitellogenesis and choriogenesis (eggshell formation). In insects such as *Drosophila* and *Bombyx*, Vmp is synthesized by and secreted from the cells of the follicular epithelium [59]. In *Drosophila*, eggshell constituents are synthesized in the follicle cells at the beginning of the vitellogenic stage (stage 8) in a well-defined spatial and temporal pattern reflecting their contribution to the eggshell. Vmp gene is expressed earlier than chorion gene, which begins at late stage (stage 11) and proceeds until the end of oogenesis [60]. In *B. mori*, mutation of Vmp is investigated in the egg-lethal phenotype [61]. Another Vmp (Vmp90) is identified to play an essential role in the developing ovarian follicle [62]. In the present study, Vmp26Aa identified in ov-6 was found to be considerably abundant until the mature stage. This protein also showed higher transcriptional expression in ov-6 (Figure 7). Similar to *Drosophila*, high abundance of chorion and chorion peroxidase were also investigated in mature ovary at both transcriptional and protein levels (Figure 8). In addition to the component of eggshell, an ovarian follicular epithelium protein was identified to be expressed exclusively in the cells during specific stages of vitellogenesis and functionally associated with vitelline membrane that contributes to the structural integrity of the follicle [63]. Chorion is produced during the late stages of oogenesis by epithelial follicle cells and develops into a highly organized multi-layered structure that exhibits regional specialization and radial complexity. In this study, a chorion s36 was identified as highly abundant in the mature ovary. In *Drosophila*, a homologous protein plays a crucial role in regulating the morphogenetic integrity of dorsal appendages in follicles inducing severe structural irregularities on chorion’s surface and entirely impairing fly’s fertility [64]. Moreover, a chorion peroxidase was also found to be abundant in mature ovary. This specific peroxidase is also identified in *D. melanogaster* and *B. oleae*, and is reported to be involved in the chorion hardening process through protein crosslinking mediated by the formation of di- and tri-tyrosine bonds [65,66].

Ovarian development is triggered by the steroid hormone, 20-hydroxy-ecdysone, which plays key role in *Drosophila* oogenesis, as its activity is required at multiple steps during egg chamber maturation [67]. Recently, its involvement has been reported on eggshell production by controlling chorion gene transcription and amplification [68]. A Halloween gene cytochrome, P450 306a1 (Phantom), was identified abundant in the vitellogenic stage of oogenesis in *B. dorsalis*. Transcriptional data also showed its high expression. A JHEH1 was also found to be highly expressed in the vitellogenic stage in this study, revealing a complementary regulation during ovarian development. Similarly, insulin receptor was found to be abundant in mature ovary. It was previously reported that insulin plays a role in ovarian development in *B. dorsalis* [69]. It is well known that in *Blattella germanica*, insulin receptor regulates juvenile hormone biosynthesis and vitellogenin production through nutritional signals [70]. In addition, regulation of notch signal pathway in follicle formation was investigated in mouse ovary [71]. Proteins involved in these pathways were also identified to be abundant in the ovary development of *B. dorsalis*.

## 3. Materials and Methods

### 3.1. Insects Culture

The stock flies were originally collected from Hainan Province of China in 2008. All insects were maintained at 27.5 ± 0.5 °C, 75 ± 5% relative humidity, and a 14:10 h (light: dark) photoperiod as described previously [4]. Under these conditions, the flies grew well and adults reached sexual maturity at 8–9 days after emergency [2]. Newly emerged female adults were separated immediately and ovaries were dissected from 1-, 3-, 5-, 7-, 9-day-old virgin females for image capture (Leica Microsystems, Wetzlar, Germany). Because of the differences in ovarian development, only ovaries of 6-day-old adults that appeared similar to the graphics between Figure 1C to Figure 1D were collected. All samples were stored as −80 °C before isolating the proteins. Two biological replicates were prepared for each sample.

### 3.2. Protein Extraction

All samples were powdered in liquid nitrogen and the powder was dissolved in 200 μL lysis buffer (pH = 8.3) containing 7 M urea, 2 M thiourea, and 20 mM Tris. Then, 800 μL of cold acetone containing 10 mM DTT was added and the mixture was incubated for 2 h and centrifuged at 13,000 rpm for 20 min at 15 °C, and the supernatants were discarded. Then, 800 μL of cold acetone containing 10 mM DTT was added and the mixture was incubated at 56 °C for 1 h to break the disulfide bonds in proteins followed by centrifugation as above. The precipitate was dissolved in 100 μL of lysis buffer and the protein concentration in the solute was determined by using the Bradford method with bovine serum albumin as a standard [72].

### 3.3. Sample Digestion and Labeling

The extracted protein (100 μg) was first diluted in 100 μL dissolution buffer. Then, 2 μg of trypsin (Promega, Madison, WI, USA) was added and the mixture was diluted with 500 μL NH_4_HCO_3_ (50 mM) and incubated for 16 h at 37 °C for protein digestion. After protein digestion, equal volume of 0.1% formic acid was added for acidulation. Peptides were purified on Strata –X C18 pillar, which was activated with methanol and then balanced with 1 mL of 0.1% formic acid three times. Then, the peptides were washed with 0.1% formic acid +5% acetonitrile twice and eluted with 1 mL of 0.1% formic acid +80% acetonitrile. Eluted peptides were vacuum dried (ThermoFisher Scientific, Asheville, NC, USA) and the dried peptides powder was dissolved in 20 μL of 0.5 M tetraethylammonium bromide (TEAB) for peptide labeling. Samples were labeled with the iTRAQ Reagent-8 plex Multiplex Kit (AB Sciex, Framingham, MA, USA) according to the manufacturer’s instructions. Six samples were labeled with different iTRAQ tags: 113 and 114 for ov-1, 115 and 116 for ov-6, and 117 and 118 for ov-9. Equal amounts of the labeled samples were pooled together and then fractionated using high-performance liquid chromatography (HPLC) (Thermo Scientific DINOEX Ultimate 3000 BioRS, Waltham, MA, USA) using a Durashell C18 (5 μm, 100 Å, 4.6 × 250 mm). Finally, 12 fractions were collected.

### 3.4. LC-MS/MS Analysis

LC-MS/MS analysis was performed on an AB SCIEX nanoLC-MS/MS system (Triple TOF 5600 plus, Framingham, MA, USA). Samples were chromatographed on a 120-min gradient from 2–35% (buffer A 0.1% formic acid, 5% acetonitrile, buffer B 0.1% formic acid, 95% acetonitrile) after direct injection onto a 20 cm PicoFrit emitter (New Objective) packed to 20 cm with Magic C18 with an inner diameter of 3 μm at 200 Å stationary phase. MS1 spectra were collected in the range of 360–1460 *m*/*z* for 250 ms. The 20 most intense precursors with charge state 2–5 were selected for fragmentation, and MS2 spectra were collected in the range of 50–2000 *m*/*z* for 100 ms; precursor ions were excluded from reselection for 15 s.

### 3.5. Protein Identification and Functional Annotation

The original MS/MS file data were submitted to the ProteinPilot Software (AB SCIEX, version 4.0) for data analysis. For protein identification, the Paragon algorithm, which was integrated into ProteinPilot was employed against uniprot database for database searching [73]. Cysteine was modified with iodoacetamide; biological modifications were selected as ID focus, trypsin digestion, the Quantitate, Bias Correction, and Background Correction was checked for protein quantification and normalization. Only proteins with more than one peptides and unused value ≥1.3 were considered for identification.

To determine the biological and functional properties, all the identified proteins were mapped with Gene Ontology Terms (available online: http://geneontology.org/). For this, homology search was first performed for all the identified proteins with a localized NCBI BlastP program against the UniProt database of *B. dorsalis* species. The e-value was set to less than 10^−5^, and the best hit for each query sequence was taken into account for GO term matching, which was performed with blast2go v4.5 pipeline [74]. Clusters of Orthologous Groups of Proteins System (COG, available online: http://www.ncbi.nlm.nih.gov/COG/) was employed for the functional annotation of genes from new genomes and for research on genome evolution. To identify candidate biomarkers, we employed hypergeometric test to perform GO enrichment and Pathway enrichment. The GO and COG assignment were also performed within the DEPs in this study.

### 3.6. Protein Quantitative Analysis

DEPs were determined based on the ratios of differently labeled proteins and *P* values provided by Proteinpilot. Fold change was calculated as the average comparison pairs among biological replicates. We set restrictive conditions to find the potential proteins involved in the ovarian development in this study. Only the proteins with expression fold changes ≥1.5- or ≤0.67-fold between all the comparisons of biological replicates, as well as *P* value of all the differences between protein abundance comparisons <0.05 were identified as DEPs. All the DEPs between each comparison were functionally annotated as above. In order to screen for potential target proteins involved in the pre-vitellogenic and vitellogenic stages, proteins highly abundant in each independent stage were thereafter functionally classified.

### 3.7. RNA Extraction and RT-qPCR Analysis

To examine the transcriptional expression of potential functional proteins with high expression in each staged ovary, total RNA from ov-1, ov-6, and ov-9 with the similar morphological ovaries were isolated using TRIZoL reagent (Invitrogen, Carlsbad, CA, USA) following the manufacturer’s instructions. First-strand cDNA was obtained from DNase I-treated RNA (~1 μg) using a PrimeScript 1st Strand cDNA Synthesis Kit (Takara, Dalian, China). Some DEPs highly expressed in ov-1, ov-6, and ov-9 were randomly selected for qRT-PCR analysis. PCR primers for each DEP were designed using an online tool, Primer 3, based on the corresponding nucleotide sequences in NCBI. For control purposes, a fragment of α-*tubulin* open reading frame was also amplified using gene specific primers [75]. All PCR primers used in the research presented here were list in Table S5. Each reaction consisted of a 10 μL volume containing 0.5 μL of cDNA template, 5 μL of GoTaq qPCR Master Mix (Promega, Madison, WI, USA), 0.5 μL of each primer (10 μM) and 3.5 μL of nuclease-free water. The reaction conditions and calculation of gene expression were essentially as described before [9].

## 4. Conclusions

In conclusion, a total of 4838 proteins were identified in *B. dorsalis* ovaries. Among these, 612 and 196 proteins were differentially expressed in vitellogenic and mature ovaries, respectively. Together, we identified 153, 44, and 59 proteins to be highly abundant in 1-, 6- and 9-day-old ovaries, respectively. Many DEPs were transcriptionally validated and showed consistent profiles at both transcriptional and translational levels. Many potential target proteins that were highly expressed in the three ovarian stages, including tropomyosin, vitellogenin, eukaryotic translation initiation factor, importin protein, vitelline membrane protein, and chorion protein. Some hormone and signal pathway related proteins were also identified during ovarian development, such as piRNA, notch, insulin, juvenile and ecdysone hormone signal pathways. This is the first report of a global proteome in a tephritid ovary and contributes to our understanding of the complicated processes of ovarian development in insects. These results will also aid in the identification of novel target proteins for improving strategies to control tephritid fruit flies.

## Figures and Tables

**Table 1:** Proteins highly abundant in ovary of 1-day-old adult of *Bactrocera dorsalis*.

Protein ID	Annotation	Length	Coverage	Peptides	Ov-6/Ov-1	Ov-9/Ov-1	Ov-9/Ov-6 ^a,b^
A0A034WJQ4	Fat-body protein 1	511	48.53	31	↓ 0.02 ± 0	↓ 0.02 ± 0	↑ 50.24 ± 28.2 ^a^
A0A034V3B3	Arylphorin subunit A4	1224	45.92	55	↓ 0.02 ± 0	↓ 0.04 ± 0.01	↑ 4.95 ± 2.14 ^a^
A0A034VYM4	Larval serum protein 1 gamma chain	773	31.31	27	↓ 0.02 ± 0	↓ 0.03 ± 0	↑ 2.95 ± 1.03 ^a^
A0A034V6N3	High mobility group protein Z	112	33.04	5	↓ 0.02 ± 0	↓ 0.02 ± 0	↑ 1.87 ± 0.47 ^a^
A0A034WL03	Development-specific 25 kDa protein	259	69.5	19	↓ 0.03 ± 0	↓ 0.04 ± 0	↑ 3.04 ± 1 ^a^
A0A034W585	Larval cuticle protein A3A	138	75.36	17	↓ 0.03 ± 0	↓ 0.04 ± 0	↑ 2.3 ± 0.55 ^a^
A0A034WSW4	Larval cuticle protein 8	104	22.12	2	↓ 0.03 ± 0	↓ 0.04 ± 0.01	↑ 1.98 ± 0.23 ^a^
A0A034WXX0	Histone H1	240	30.83	11	↓ 0.03 ± 0.01	↓ 0.03 ± 0	↑ 8.18 ± 4.64 ^a^
A0A034WP07	Attacin-A	239	72.8	14	↓ 0.03 ± 0.01	↓ 0.13 ± 0.03	↑ 6.37 ± 1.87 ^a^
A0A034V3D6	J domain-containing protein	177	70.62	15	↓ 0.04 ± 0.01	↓ 0.03 ± 0	0.84 ± 0.25 ^b^
A0A034W4Q0	Larval cuticle protein A2B	180	73.89	17	↓ 0.05 ± 0	↓ 0.06 ± 0	↑ 1.61 ± 0.32 ^a^
A0A034VT34	Angiopoietin-4	454	20.04	7	↓ 0.05 ± 0	↓ 0.06 ± 0.01	1.39 ± 0.36 ^b^
A0A034W9Y1	Larval cuticle protein A2B	250	92.4	19	↓ 0.05 ± 0.01	↓ 0.06 ± 0	↑ 1.73 ± 0.6 ^a^
A0A034VU29	Regucalcin	351	64.96	26	↓ 0.05 ± 0.01	↓ 0.05 ± 0.01	1.49 ± 0.42 ^b^
I1T1H2	Heat shock protein 20	170	67.65	10	↓ 0.05 ± 0.02	↓ 0.13 ± 0.03	↑ 4.75 ± 1.43 ^a^
A0A034VSB5	Fasciclin-1	542	35.42	17	↓ 0.07 ± 0	↓ 0.56 ± 0.04	↑ 8.35 ± 0.24
A0A034WRI9	Sarcocystatin-A	125	47.2	6	↓ 0.07 ± 0.02	↓ 0.15 ± 0.04	↑ 3.48 ± 1.58 ^a^
A0A034WF27	TPPP family protein CG4893	201	40.3	7	↓ 0.08 ± 0.01	↓ 0.09 ± 0.02	1.12 ± 0.28 ^b^
A0A034VUV3	Lamin Dm0	617	71.64	46	↓ 0.08 ± 0.02	↓ 0.06 ± 0	0.8 ± 0.28 ^b^
A0A034WSV2	Lamin-C	622	56.27	37	↓ 0.09 ± 0.01	↓ 0.06 ± 0	↓ 0.63 ± 0.13 ^a^
A0A034V8W7	Cytochrome P450 4g15	484	23.76	10	↓ 0.09 ± 0.01	↓ 0.11 ± 0.03	1.27 ± 0.29 ^b^
A0A034VPE5	Tropomyosin-1, isoforms 9A/A/B	188	69.68	19	↓ 0.09 ± 0.01	↓ 0.1 ± 0.02	0.92 ± 0.09 ^b^
A0A034V2U0	Protein 4.1-like protein	1703	57.37	82	↓ 0.09 ± 0.02	↓ 0.07 ± 0	0.96 ± 0.24 ^b^
A0A034VW09	Protein Skeletor	741	29.82	14	↓ 0.1 ± 0.01	↓ 0.21 ± 0.05	↑ 2.25 ± 0.46 ^a^
A0A034W3W2	Regucalcin	296	42.91	12	↓ 0.1 ± 0.02	↓ 0.08 ± 0.01	0.87 ± 0.25 ^b^
A0A034V7Q2	Protein yellow	261	31.42	6	↓ 0.1 ± 0.03	↓ 0.13 ± 0.01	1.48 ± 0.41 ^b^
A0A034VI82	Tropomodulin	386	34.97	10	↓ 0.11 ± 0	↓ 0.08 ± 0	↓ 0.62 ± 0.07 ^a^
A0A034VUS5	Integrin-linked protein kinase	448	32.37	14	↓ 0.12 ± 0.01	↓ 0.13 ± 0.02	1.15 ± 0.3 ^b^
A0A034VKK9	Alcohol dehydrogenase	324	38.89	13	↓ 0.12 ± 0.01	↓ 0.13 ± 0.02	0.94 ± 0.2 ^b^
A0A034VYW9	Contactin	1393	25.2	27	↓ 0.12 ± 0.02	↓ 0.21 ± 0.04	↑ 2.08 ± 0.42 ^a^
A0A034WVT6	Membrane metallo-endopeptidase-like 1	714	22.13	14	↓ 0.12 ± 0.02	↓ 0.15 ± 0.01	1.18 ± 0.19 ^b^
A0A034V5Q1	Ras suppressor protein 1	138	39.86	5	↓ 0.13 ± 0.02	↓ 0.19 ± 0.03	↑ 1.67 ± 0.41 ^a^
A0A034W0B4	Chitinase-like protein CG5210	279	51.25	14	↓ 0.13 ± 0.04	↓ 0.37 ± 0.04	↑ 4.97 ± 1.76 ^a^
A0A034WBA7	Putative G-protein coupled receptor 158	809	29.05	20	↓ 0.14 ± 0.02	↓ 0.28 ± 0.04	↑ 2.24 ± 0.56 ^a^
A0A034V5R2	Talin-1	1928	48.86	69	↓ 0.15 ± 0.01	↓ 0.45 ± 0.02	↑ 3.15 ± 0.09
A0A034VQZ0	Zinc finger protein 512B	427	26.23	7	↓ 0.15 ± 0.01	↓ 0.13 ± 0.02	0.84 ± 0.16 ^b^
A0A034VDB5	Protein lap4	915	20.11	14	↓ 0.16 ± 0	↓ 0.11 ± 0.01	↓ 0.6 ± 0.09 ^a^
A0A034VTK3	Chitinase-like protein Idgf5	432	27.31	9	↓ 0.16 ± 0.05	↓ 0.16 ± 0.01	1.27 ± 0.41 ^b^
A0A034W8P6	Protein E(Sev)2B	211	43.6	9	↓ 0.17 ± 0.03	↓ 0.29 ± 0.02	↑ 2.05 ± 0.44 ^a^
A0A034VVP8	Serpin B9	504	29.76	14	↓ 0.18 ± 0.01	↓ 0.27 ± 0.02	1.49 ± 0.12 ^b^
A0A034W4R7	α,α-trehalose-phosphate synthase A	813	33.46	24	↓ 0.18 ± 0.02	↓ 0.09 ± 0.01	↓ 0.41 ± 0.06 ^a^
A0A034WI29	Protein lin-7-like protein B	195	28.72	5	↓ 0.18 ± 0.03	↓ 0.19 ± 0.02	1.1 ± 0.18 ^b^
A0A034VY88	Protein hu-li tai shao	698	69.63	35	↓ 0.19 ± 0.01	↓ 0.2 ± 0.02	1.07 ± 0.09 ^b^
A0A034W1X2	Alaserpin	398	27.39	10	↓ 0.19 ± 0.02	↓ 0.42 ± 0.03	↑ 2.43 ± 0.28 ^a^
A0A034W8L1	Flotillin-2	425	43.53	17	↓ 0.19 ± 0.02	↓ 0.13 ± 0.02	↓ 0.63 ± 0.08 ^a^
A0A034VQR8	Troponin T, skeletal muscle	384	39.32	22	↓ 0.19 ± 0.02	↓ 0.07 ± 0.01	↓ 0.31 ± 0.07 ^a^
A0A034WRQ2	α-parvin	366	33.06	10	↓ 0.19 ± 0.05	↓ 0.26 ± 0.05	↑ 1.58 ± 0.39 ^a^
A0A034VP83	Poly(U)-specific endoribonuclease-like protein	675	24.74	13	↓ 0.2 ± 0.01	↓ 0.35 ± 0.03	↑ 1.79 ± 0.16 ^a^
A0A034WSM6	Guanine nucleotide-binding protein G(S) subunit α	382	32.2	10	↓ 0.2 ± 0.02	↓ 0.36 ± 0.02	↑ 1.88 ± 0.18 ^a^
Q86QQ0	Glutathione S-transferase	209	37.32	6	↓ 0.21 ± 0.01	↓ 0.17 ± 0.01	0.81 ± 0.07 ^b^
A0A034VND6	Tropomyosin-1, isoforms 33/34	282	48.58	20	↓ 0.21 ± 0.04	↓ 0.13 ± 0.02	↓ 0.61 ± 0.13 ^a^
A0A034VSX0	Phosphate-regulating neutral endopeptidase	683	22.69	13	↓ 0.21 ± 0.04	↓ 0.16 ± 0.02	0.85 ± 0.16 ^b^
A0A034V6M3	60S ribosomal protein L23a	266	26.32	10	↓ 0.21 ± 0.05	↓ 0.26 ± 0.07	↑ 1.62 ± 0.78 ^a^
A0A034UZS9	PDZ and LIM domain protein	495	22.22	9	↓ 0.22 ± 0.01	↓ 0.19 ± 0.04	0.87 ± 0.25 ^b^
A0A034VR92	Tropomyosin-2	284	57.04	17	↓ 0.22 ± 0.02	↓ 0.06 ± 0	↓ 0.22 ± 0.02
A0A034V2K7	Spectrin β chain, non-erythrocytic 5	4200	26.48	94	↓ 0.22 ± 0.02	↓ 0.29 ± 0.03	1.32 ± 0.04 ^b^
A0A034VDP0	Obscurin	631	30.74	13	↓ 0.22 ± 0.03	↓ 0.15 ± 0.02	0.71 ± 0.19 ^b^
A0A034UX56	Trehalose 6-phosphate phosphatase	274	81.75	25	↓ 0.23 ± 0.01	↓ 0.03 ± 0	↓ 0.08 ± 0.01
A0A034VE41	Twitchin	4978	23.54	86	↓ 0.23 ± 0.02	↓ 0.14 ± 0.01	↓ 0.57 ± 0.05 ^a^
A0A034WWI9	Ejaculatory bulb-specific protein 3	127	22.05	3	↓ 0.23 ± 0.02	↓ 0.18 ± 0.04	0.77 ± 0.14 ^b^
A0A034VPA5	Heterogeneous nuclear ribonucleoprotein Q	417	21.34	9	↓ 0.23 ± 0.05	↓ 0.15 ± 0.01	0.69 ± 0.15 ^b^
A0A034VWH3	Myosin regulatory light chain 2	222	52.7	10	↓ 0.25 ± 0.02	↓ 0.12 ± 0.02	↓ 0.41 ± 0.06 ^a^
A0A034VAC6	Protein elav	446	35.65	12	↓ 0.26 ± 0.02	↓ 0.29 ± 0.04	1.08 ± 0.17 ^b^
A0A034V4T6	Fasciclin-2	520	45.19	17	↓ 0.26 ± 0.03	↓ 0.08 ± 0.01	↓ 0.27 ± 0.02 ^a^
A0A034VRQ6	Protein held out wings	356	37.64	11	↓ 0.28 ± 0.01	↓ 0.15 ± 0.01	↓ 0.48 ± 0.02 ^a^
A0A034WML8	Protein takeout	269	20.07	4	↓ 0.29 ± 0.02	↓ 0.14 ± 0.01	↓ 0.46 ± 0.05 ^a^
A0A034WF58	Glutathione peroxidase	278	48.92	13	↓ 0.29 ± 0.05	↓ 0.26 ± 0.03	0.94 ± 0.18 ^b^
A0A034VTK4	DNA topoisomerase 2	1481	42.13	59	↓ 0.3 ± 0.02	↓ 0.24 ± 0.01	0.76 ± 0.06 ^b^
A0A034WS42	Histone H4	103	65.05	15	↓ 0.3 ± 0.06	↓ 0.08 ± 0.01	↓ 0.24 ± 0.05
A0A034WN98	17-β-hydroxysteroid dehydrogenase 13	326	25.15	7	↓ 0.33 ± 0.05	↓ 0.3 ± 0.07	0.98 ± 0.27 ^b^
A0A034V7N7	Protein lethal(2) giant larvae	1176	24.23	21	↓ 0.33 ± 0.05	↓ 0.24 ± 0.04	0.67 ± 0.11 ^b^
M1F3Z9	Glutathione S-transferase ω-1	255	46.67	11	↓ 0.34 ± 0.02	↓ 0.26 ± 0.02	0.76 ± 0.06 ^b^
A0A034VDX0	L-2-hydroxyglutarate dehydrogenase, mitochondrial	455	31.21	10	↓ 0.35 ± 0.03	↓ 0.33 ± 0.07	0.95 ± 0.23 ^b^
A0A034VM61	Clavesin-2	320	33.44	9	↓ 0.35 ± 0.04	↓ 0.45 ± 0.07	1.24 ± 0.16 ^b^
A0A034VPR7	Putative peptidyl-prolyl cis-trans isomerase dodo	160	57.5	11	↓ 0.35 ± 0.06	↓ 0.32 ± 0	0.99 ± 0.18 ^b^
A0A034WSZ7	Heterochromatin protein 1	212	23.11	5	↓ 0.37 ± 0.02	↓ 0.24 ± 0.01	↓ 0.6 ± 0.01 ^a^
A0A034WQQ0	Calcyphosin-like protein	219	27.4	6	↓ 0.37 ± 0.1	↓ 0.35 ± 0.06	1.04 ± 0.21 ^b^
A0A034WBM1	Acetyl-CoA acetyltransferase, cytosolic	394	41.62	11	↓ 0.41 ± 0.08	↓ 0.45 ± 0.05	1.24 ± 0.3 ^b^
A0A034VYI5	Non-specific protein-tyrosine kinase	510	27.25	12	↓ 0.45 ± 0.07	↓ 0.34 ± 0.01	0.79 ± 0.12 ^b^
A0A034VW38	SUN domain-containing protein 1	594	21.55	13	↓ 0.45 ± 0.1	↓ 0.31 ± 0.09	0.7 ± 0.14 ^b^
A0A034VQG5	Nucleoprotein TPR	2410	26.22	53	↓ 0.47 ± 0.03	↓ 0.26 ± 0.01	↓ 0.49 ± 0.05 ^a^
A0A034VKE6	Bifunctional methylenetetrahydrofolate dehydrogenase/cyclohydrolase, mitochondrial	316	32.59	8	↓ 0.5 ± 0.01	↓ 0.21 ± 0.05	↓ 0.43 ± 0.08 ^a^
A0A034VPM4	Vinculin	960	50.63	38	↓ 0.54 ± 0.04	↓ 0.54 ± 0.04	0.98 ± 0.07 ^b^
A0A034VRI7	α-actinin, sarcomeric	895	62.35	53	↓ 0.55 ± 0.04	↓ 0.42 ± 0.02	0.75 ± 0.04 ^b^

^a^ the fold change is ≥1.5- or ≤0.67-fold but at least one *p*-value ≥ 0.05; ^b^ no difference of protein abundance; “↑” represents protein abundance up-regulation; “↓” represents protein abundance down-regulation; Proteins with no functional annotation, and also sequence coverage ≤20% were not listed in this table. Ov-1, ov-6 and ov-9 represent the ovary from 1-, 6- and 9-day-old *B. dorsalis* adult.

**Table 2:** Proteins highly abundant in ovary of 6-d-old adult of *Bactrocera dorsalis*.

Protein ID	Annotation	Length	Coverage	Peptides	Ov-6/Ov-1	Ov-9/Ov-1 ^a,b^	Ov-9/Ov-6
A0A034V9A3	CUGBP Elav-like family member 2	587	25.55	13	↑ 29.06 ± 4.88	↑ 13.14 ± 6.66 ^a^	↓ 0.37 ± 0.06
A0A034WDZ9	Importin-5	1106	53.35	47	↑ 18.4 ± 2.73	↑ 11.19 ± 4.34	↓ 0.53 ± 0.04
A0A034VSG4	Maternal protein exuperantia	495	51.11	26	↑ 17 ± 1.02	↑ 5.37 ± 1.74 ^a^	↓ 0.27 ± 0.04
A0A034WW15	40S ribosomal protein S3	244	80.33	26	↑ 16.58 ± 0.53	↑ 10.31 ± 1.82	↓ 0.54 ± 0.05
A0A034VN85	Eukaryotic translation initiation factor 3 subunit E	434	38.71	15	↑ 11.54 ± 0.68	↑ 5.3 ± 2.25 ^a^	↓ 0.42 ± 0.08
A0A034WLA5	ATP-dependent RNA helicase vasa, isoform A	621	57.49	35	↑ 11.45 ± 3.76	↑ 6.59 ± 4.91 ^a^	↓ 0.51 ± 0.04
A0A034WFM3	60S ribosomal protein L3	413	50.36	26	↑ 10.66 ± 1.06	↑ 5.58 ± 2.23	↓ 0.51 ± 0.07
A0A034WMJ8	40S ribosomal protein S4	280	59.29	23	↑ 10.35 ± 1.61	↑ 4.44 ± 2.48 ^a^	↓ 0.41 ± 0.1
A0A034VGJ1	Clustered mitochondria protein	1362	30.54	35	↑ 9.85 ± 2.74	↑ 5.7 ± 3.47	↓ 0.53 ± 0.02
A0A034V813	Acetyl-CoA carboxylase	2391	34.84	58	↑ 9.8 ± 1.2	↑ 5.48 ± 1.47	↓ 0.51 ± 0.03
A0A034WGD2	Ribosomal L1 domain-containing protein	608	32.07	18	↑ 9.37 ± 0.71	↑ 2.86 ± 1.34 ^a^	↓ 0.29 ± 0.06
A0A034VH20	Piwi-like protein	900	49.56	37	↑ 8.88 ± 2.99	↑ 3.75 ± 3.08 ^a^	↓ 0.35 ± 0.05
A0A034VGU8	Eukaryotic translation initiation factor 2 subunit 3	475	45.68	16	↑ 8.09 ± 1.03	↑ 4.25 ± 1.63 ^a^	↓ 0.49 ± 0.05
A0A034VGS8	Tubulin β-3 chain	454	60.79	27	↑ 7.51 ± 0.22	↑ 3.32 ± 1.16 ^a^	↓ 0.42 ± 0.08
A0A034V5U3	Staphylococcal nuclease domain-containing protein 1	928	64.12	47	↑ 7.34 ± 0.89	↑ 2.91 ± 1.09 ^a^	↓ 0.35 ± 0.04
A0A034VX01	DNA-binding protein modulo	578	37.89	22	↑ 7.2 ± 1.01	↑ 2.62 ± 0.83 ^a^	↓ 0.35 ± 0.01
A0A034VMS2	Peptide methionine sulfoxide reductase	243	29.63	6	↑ 6.68 ± 0.34	↑ 3.19 ± 0.6 ^a^	↓ 0.45 ± 0.04
A0A034VUY6	Methenyltetrahydrofolate synthase domain-containing protein	538	56.51	32	↑ 5.91 ± 0.39	1.09 ± 0.49 ^b^	↓ 0.16 ± 0.03
A0A034WNF6	Importin subunit α	519	29.09	12	↑ 5.51 ± 0.44	1 ± 0.32 ^b^	↓ 0.19 ± 0.02
A0A034VER3	Eukaryotic translation initiation factor 3 subunit C	923	37.92	32	↑ 5.49 ± 0.72	↑ 2.35 ± 1.36 ^a^	↓ 0.42 ± 0.09
A0A034W2L3	Citrate synthase	257	63.42	15	↑ 5.37 ± 0.42	↑ 1.75 ± 1.24 ^a^	↓ 0.31 ± 0.09
A0A034VH70	La-related protein	1206	24.71	20	↑ 5.31 ± 0.28	↑ 2.77 ± 0.4 ^a^	↓ 0.5 ± 0.03
A0A034VA48	Prolyl 4-hydroxylase subunit α2	561	23.53	9	↑ 5.19 ± 0.6	1.08 ± 0.28 ^b^	↓ 0.21 ± 0.01
A0A034W6X9	GTP-binding protein 128up	368	52.45	16	↑ 5.12 ± 0.22	↑ 2.39 ± 0.93 ^a^	↓ 0.48 ± 0.08
A0A034WIR4	DnaJ-like protein subfamily C member 2	618	28.48	14	↑ 4.9 ± 0.62	↑ 2.81 ± 0.79 ^a^	↓ 0.56 ± 0.03
A0A034WLF6	FK506-binding protein 59	437	59.27	29	↑ 4.84 ± 0.23	↑ 2.14 ± 1.3 ^a^	↓ 0.41 ± 0.11
A0A034V6Q5	mRNA turnover protein 4-like protein	257	28.79	8	↑ 4.61 ± 0.36	↑ 2.13 ± 0.13 ^a^	↓ 0.47 ± 0.04
A0A034W8Z3	Putative ATP-dependent RNA helicase DDX43	662	47.28	27	↑ 4.6 ± 0.28	↑ 2.5 ± 0.55 ^a^	↓ 0.51 ± 0.04
A0A034VYR3	Nucleolar protein 58	728	43.82	30	↑ 4.56 ± 0.83	↑ 1.98 ± 1.14 ^a^	↓ 0.41 ± 0.07
A0A034WSY7	Nucleolar GTP-binding protein	651	23.04	13	↑ 3.91 ± 0.06	1.14 ± 0.41 ^b^	↓ 0.28 ± 0.05
A0A034V087	Guanine nucleotide-binding-like protein 3-like protein	610	29.02	16	↑ 3.67 ± 0.47	0.76 ± 0.24 ^b^	↓ 0.2 ± 0.01
A0A034V7N4	Myosin heavy chain 95F	1240	28.63	32	↑ 3.6 ± 0.57	1.09 ± 0.56 ^b^	↓ 0.3 ± 0.06
A0A034VCY4	Tudor domain-containing protein 7	956	25.63	18	↑ 3.41 ± 0.76	0.85 ± 0.49 ^b^	↓ 0.25 ± 0.05
A0A034WCL6	Peptidyl-prolyl cis-trans isomerase FKBP6	478	43.93	16	↑ 3.22 ± 0.32	↑ 1.53 ± 0.39 ^a^	↓ 0.46 ± 0.04
A0A034VFT5	tRNA (Cytosine(34)-C(5))-methyltransferase	728	33.52	16	↑ 3.05 ± 0.54	↑ 1.78 ± 0.68 ^a^	↓ 0.57 ± 0.03
A0A034VFV1	Cytosolic 10-formyltetrahydrofolate dehydrogenase	687	47.02	23	↑ 3.04 ± 0.23	1.42 ± 0.26 ^b^	↓ 0.48 ± 0.04
A0A034VJX4	Very long-chain fatty-acid--CoA ligase bubblegum	666	30.33	15	↑ 2.95 ± 0.41	1.3 ± 0.44 ^b^	↓ 0.43 ± 0.04

^a^ the fold change is ≥1.5- or ≤0.67-fold but at least one *p*-value ≥ 0.05; ^b^ no difference of protein abundance; “↑” represents protein abundance up-regulation; “↓” represents protein abundance down-regulation; Proteins with no functional annotation, and also sequence coverage ≤20% were not listed in this table. Ov-1, ov-6 and ov-9 represent the ovary from 1-, 6- and 9-day-old *B. dorsalis* adult.

**Table 3:** Proteins highly abundant in ovary of 9-day-old adult of *Bactrocera dorsalis*.

Protein ID	Annotation	Length	Coverage	Peptides	Ov-6/Ov-1 ^a,b^	Ov-9/Ov-1	Ov-9/Ov-6
A0A034WSR6	Defective chorion-1 protein	723	28.49	25	↑ 27.31 ± 1.25	↑ 45.64 ± 1.03	↑ 2.8 ± 0.2
A0A034W812	Importin subunit α	520	63.65	22	↑ 10.77 ± 0.98	↑ 26.6 ± 1.41	↑ 3.62 ± 0.43
A0A034VZV8	Chorion peroxidase	836	40.31	29	↑ 12 ± 1.27	↑ 20.11 ± 2.27	↑ 2.56 ± 0.39
A0A034UZ33	Importin subunit β	885	37.51	26	↑ 10.39 ± 1.97	↑ 16.2 ± 2.62	↑ 1.94 ± 0.02
A0A034V3F4	Tripeptidyl-peptidase 2	1353	47.97	54	↑ 4.9 ± 0.24	↑ 11.08 ± 0.67	↑ 2.61 ± 0.16
A0A034V085	Proteasome-associated protein ECM29-like protein	1889	26.95	41	↑ 7.64 ± 2.22	↑ 11.06 ± 3.02	↑ 1.6 ± 0.04
A0A034VPR3	Elongation factor Tu GTP-binding domain-containing protein 1	1043	31.06	28	↑ 7.36 ± 0.75	↑ 10.71 ± 1	↑ 1.62 ± 0.04
A0A034WID4	Putative cation-transporting ATPase 13A1	1216	18.67	19	↑ 6.44 ± 0.93	↑ 10.57 ± 1.35	↑ 1.79 ± 0.07
A0A034VC08	Xaa-Pro dipeptidase	480	42.29	18	↑ 5.88 ± 0.25	↑ 10.51 ± 0.34	↑ 2.09 ± 0.08
A0A034VG52	Adenosylhomocysteinase	509	53.24	23	↑ 6.14 ± 0.98	↑ 9.11 ± 1.26	↑ 1.66 ± 0.04
A0A034VV21	Nuclear pore complex protein Nup205	1088	22.79	20	↑ 4.06 ± 0.49	↑ 7.84 ± 0.87	↑ 2.11 ± 0.04
A0A034VMD8	Cytoskeleton-associated protein 5	1227	26.57	26	↑ 3.15 ± 0.56	↑ 6.72 ± 1.11	↑ 2.26 ± 0.11
A0A034WDU4	Endoplasmin	797	56.21	46	↑ 3.9 ± 0.12	↑ 6.47 ± 0.19	↑ 1.74 ± 0.03
A0A034W4A7	Glutathione S-transferase 1-1	207	49.76	11	↑ 2.74 ± 0.06	↑ 5.86 ± 0.51	↑ 2.26 ± 0.2
A0A034WP50	Chorion protein S36	233	54.08	10	↑ 8.94 ± 1.36 ^a^	↑ 36.76 ± 2.19	↑ 15.69 ± 1.6
A0A034VUE5	Myotubularin-related protein 14	765	30.59	18	↑ 8.44 ± 0.67 ^a^	↑ 29.22 ± 1.46	↑ 6.21 ± 0.44
A0A034W787	Juvenile hormone epoxide hydrolase 1	459	32.46	18	↑ 16.8 ± 1.38 ^a^	↑ 26.88 ± 1.96	↑ 2.64 ± 0.29
A0A034VVU8	Muskelin	890	38.2	32	↑ 6.51 ± 0.55 ^a^	↑ 17.65 ± 3.35	↑ 4.04 ± 0.9
A0A034W030	MAP kinase-activating death domain protein	2175	7.724	13	↑ 5.65 ± 0.65 ^a^	↑ 17.31 ± 1.03	↑ 3.84 ± 0.3
A0A034W1D2	UDP-glucuronosyltransferase 1-1	640	8.594	5	↑ 4.31 ± 0.54 ^a^	↑ 12.79 ± 1.88	↑ 3.44 ± 0.53
A0A034W397	Dual specificity mitogen-activated protein kinase kinase dSOR1	397	44.58	18	↑ 5.39 ± 0.1 ^a^	↑ 11.14 ± 0.87	↑ 2.67 ± 0.14
A0A034WCE7	Insulin receptor	963	30.43	21	↑ 3.01 ± 0.76 ^a^	↑ 10.2 ± 1.62	↑ 4.24 ± 0.39
A0A034W9D5	Glutaminyl-peptide cyclotransferase-like protein	330	15.45	5	↑ 2.57 ± 0.71 ^a^	↑ 9.6 ± 1.15	↑ 4.79 ± 1
A0A034WBX7	Ribonucleoside-diphosphate reductase	804	46.27	34	↑ 2.49 ± 0.45 ^a^	↑ 9.34 ± 1.45	↑ 3.83 ± 0.28
A0A034V8K5	Aminopeptidase N	1035	19.81	16	↑ 2.64 ± 0.68 ^a^	↑ 7.97 ± 1.66	↑ 3.55 ± 0.26
A0A034VKU6	Myotubularin-related protein 3	1279	17.98	18	↑ 1.86 ± 0.21 ^a^	↑ 7.79 ± 0.76	↑ 4.51 ± 0.12
A0A034VIB7	Protein phosphatase 1B	370	40.54	13	↑ 3± 0.31 ^a^	↑ 7.73 ± 0.75	↑ 2.73 ± 0.23
A0A034WC12	Cytosolic endo-β-*N*-acetylglucosaminidase	604	30.3	15	↑ 2.21 ± 0.39 ^a^	↑ 7.67 ± 1.22	↑ 3.88 ± 0.72
A0A034WUY1	Ubiquitin carboxyl-terminal hydrolase	227	74.89	11	↑ 4.46 ± 0.45 ^a^	↑ 7.42 ± 0.68	↑ 1.9 ± 0.07
A0A034VK02	Dihydrolipoyl dehydrogenase	504	58.33	26	↑ 3.79 ± 0.22 ^a^	↑ 6.88 ± 0.35	↑ 1.98 ± 0.15
A0A034VAA7	Centrosomin	992	10.58	8	↑ 1.57 ± 0.21 ^a^	↑ 6.77 ± 0.68	↑ 4.8 ± 0.35
A0A034W647	Peptidyl-prolyl cis-trans isomerase D	442	34.62	15	↑ 2.29 ± 0.07 ^a^	↑ 6.55 ± 0.29	↑ 2.98 ± 0.2
G9F9Y5	Chitinase	483	24.43	8	1.18 ± 0.17 ^b^	↑ 6.26 ± 0.61	↑ 5.86 ± 0.67
A0A034WDM9	26S protease regulatory subunit 6A	428	63.55	25	↑ 3.65 ± 0.06 ^a^	↑ 5.63 ± 0.08	↑ 1.68 ± 0.06
A0A034WB53	Cullin-5	850	16.24	12	0.95 ± 0.23 ^b^	↑ 5.13 ± 0.53	↑ 5.75 ± 1.07
A0A034W865	Gamma-tubulin complex component 3-like protein	951	19.77	15	↑ 1.86 ± 0.13 ^a^	↑ 4.98 ± 0.65	↑ 2.88 ± 0.36
A0A034WNA4	GMP synthase (Glutamine-hydrolyzing)	683	53.44	30	↑ 1.94 ± 0.1 ^a^	↑ 4.92 ± 0.18	↑ 2.68 ± 0.09
A0A034W2K6	Major royal jelly protein 1	425	40.24	14	0.67 ± 0.05 ^b^	↑ 4.68 ± 0.62	↑ 6.5 ± 0.67
A0A034WHL8	Venom carboxylesterase-6	549	25.14	12	1.31 ± 0.07 ^b^	↑ 4.68 ± 0.27	↑ 3.84 ± 0.3
A0A034WMG0	Ubiquitin carboxyl-terminal hydrolase	1110	31.08	29	↑ 2.24 ± 0.47 ^a^	↑ 4.28 ± 0.91	↑ 2.08 ± 0.18
A0A034VRJ4	Neutral α-glucosidase AB	449	45.88	18	1.1 ± 0.18 ^b^	↑ 3.91 ± 0.57	↑ 3.5 ± 0.24
A0A034VK16	Cytoskeleton-associated protein 5	734	32.7	20	↑ 2.41 ± 0.18 ^a^	↑ 3.9 ± 0.22	↑ 1.69 ± 0.06
A0A034W7H6	Cyclin-dependent kinase 5-like protein	293	14.68	4	1.22 ± 0.17 ^b^	↑ 3.82 ± 0.33	↑ 3.46 ± 0.48
A0A034V2C6	Serine/threonine-protein kinase Warts	610	16.23	7	1.41 ± 0.06 ^b^	↑ 3.62 ± 0.07	↑ 2.66 ± 0.14
A0A034W7N0	Protein NASP-like protein	427	41.22	15	↑ 1.67 ± 0.1 ^a^	↑ 3.4 ± 0.19	↑ 2.11 ± 0.12
A0A034WQ10	Cullin-4A	841	27.59	20	↑ 1.79 ± 0.14 ^a^	↑ 3.35 ± 0.17	↑ 1.92 ± 0.12
A0A034VQ97	CD109 antigen	1430	30.49	39	0.87 ± 0.14 ^b^	↑ 3.11 ± 0.21	↑ 3.78 ± 0.54
A0A034VKF8	Cysteine and histidine-rich protein 1-like protein	447	30.2	10	1.26 ± 0.03 ^b^	↑ 2.88 ± 0.13	↑ 2.34 ± 0.12
A0A034WBQ5	α-1,4 glucan phosphorylase	845	61.07	50	1.33 ± 0.11 ^b^	↑ 2.84 ± 0.33	↑ 2.2 ± 0.3
A0A034VJU9	85/88 kDa calcium-independent phospholipase A2	872	23.28	19	1.36 ± 0.11 ^b^	↑ 2.8 ± 0.08	↑ 2.17 ± 0.2
A0A034WAP1	β-ureidopropionase	385	41.3	11	0.8 ± 0.03 ^b^	↑ 1.98 ± 0.09	↑ 2.41 ± 0.12

^a^ the fold change is ≥1.5- or ≤0.67-fold but at least one *p*-value ≥ 0.05; ^b^ no difference of protein abundance; “↑” represents protein abundance up-regulation; “↓” represents protein abundance down-regulation; Proteins with no functional annotation, and also sequence coverage ≤20% were not listed in this table. Ov-1, ov-6 and ov-9 represent the ovary from 1-, 6- and 9-day-old *B. dorsalis* adult.

## Supplementary Materials

Supplementary materials can be found at www.mdpi.com/1422-0067/18/7/1379/s1.
