# Supplementary material for: Comparative Proteomic Profiling Reveals Molecular Characteristics Associated with Oogenesis and Oocyte Maturation during Ovarian Development of Bactrocera dorsalis (Hendel)
Source: Int J Mol Sci. 2017 Jun 30;18(7):1379. doi: 10.3390/ijms18071379 (PMC5535872; doi:10.3390/ijms18071379)
Supplement: Supplementary file 1 [file ijms-18-01379-s001.zip › 1-ijms-200319-supplement-final.pdf]

Comparative Proteomic Profiling Reveals Molecular Characteristics Associated with Oogenesis and Oocyte Maturation during Ovarian Development of *Bactrocera dorsalis* (Hendel)

Dong Wei , Ran Li , Meng-Yi Zhang , Yu-Wei Liu , Zheng Zhang , Guy Smagghe and Jin-Jun Wang

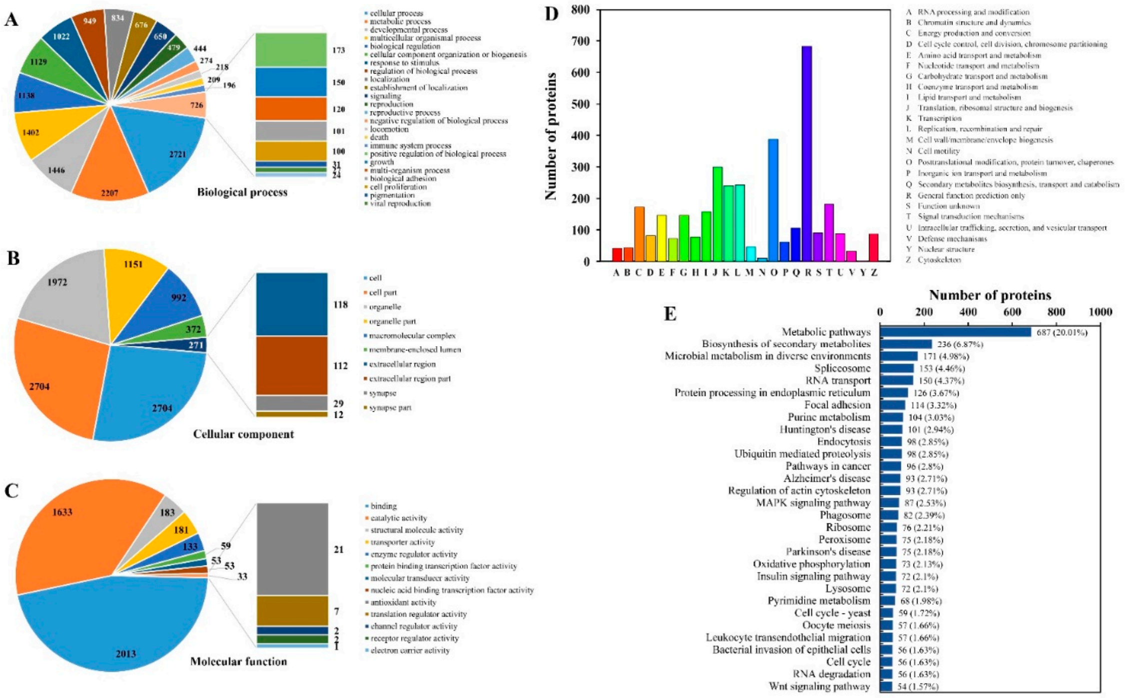

Figure S1. Functional annotation of proteins identified in Bactrocera dorsalis ovary. Gene Ontology analysis showed three ontologies were analyzed including biological process (**A**), cellular component (**B**) and molecular function (**C**); Cluster of Orthologous Groups of proteins (**D**); Top 30 pathways with highest protein number as determined by Kyoto Encyclopedia of Gene and Genomes analysis.

Table S1. Proteins differentially expressed between 1- and 6-day-old ovaries of *Bactrocera dorsalis*.

Accession Number	Annotation	Protein Mass	Protein Length	Sequence Coverage	Peptides	Fold Change	Difference
A0A034WHP3	Vitelline membrane protein Vm26Aa	11467	114	82.46	16	35.99	up
A0A034WY57	Putative ATP-dependent RNA helicase me31b	51888	457	77.46	46	29.41	up
A0A034V9A3	CUGBP Elav-like family member 2	62542	587	25.55	13	29.06	up
A0A034WSR6	Defective chorion-1 protein (Fragment)	79162	723	28.49	25	27.31	up
A0A034VQJ3	Vitellogenin-1	47853	436	83.26	82	26.95	up
A0A034WRE4	60S ribosomal protein L30	12248	111	60.36	9	26.93	up
A0A034WWC1	General odorant-binding protein 99b	16792	143	49.65	10	26.43	up
Q8T7E3	Vitellogenin 1	47781	436	83.26	77	23.51	up
A0A034VP94	40S ribosomal protein S12	15218	139	84.17	16	22.03	up
A0A034W432	Cytochrome P450 306a1	63391	545	40.92	19	20.86	up
A0A034V196	Protein disulfide-isomerase	15091	135	51.85	6	20.18	up
A0A034WNR3	46 kDa FK506-binding nuclear protein (Fragment)	20602	180	28.89	4	19.46	up
A0A034WDZ9	Importin-5	123792	1106	53.35	47	18.40	up
A0A034W366	Guanine nucleotide-binding protein subunit beta-like protein	35592	318	79.87	26	17.92	up
A0A034W6L5	Importin-7	119886	1048	37.6	31	17.87	up
A0A034WM43	Defective chorion-1 protein, FC125 isoform (Fragment)	55679	506	31.42	13	17.64	up
A0A034VSG4	Maternal protein exuperantia	55182	495	51.11	26	17.00	up
A0A034VFV2	6-phosphogluconate dehydrogenase, decarboxylating	52780	481	63.2	27	16.85	up
A7YVH6	Cytochrome b	43024	378	2.38	1	16.66	up
A0A034WW15	40S ribosomal protein S3	27365	244	80.33	26	16.58	up
A0A034VTT0	Transketolase-like protein 2 (Fragment)	21534	202	64.85	10	16.36	up
A0A034VPT4	40S ribosomal protein S6	28294	248	48.79	17	15.92	up
A0A034VWK2	60S ribosomal protein L14	19353	166	44.58	12	15.79	up
I1SWI8	Heat shock protein 60	61054	573	77.66	47	15.44	up
A0A034V6V9	Importin-4	120939	1087	42.23	40	14.62	up
A0A034VJ67	Protein cup (Fragment)	127850	1173	22.25	24	14.35	up

A0A034WK63	40S ribosomal protein S8 (Fragment)	25654	222	45.95	14	13.86	up
A0A034V1V6	Peroxiredoxin-5, mitochondrial	19883	186	78.49	16	13.13	up
A0A034VV64	Eukaryotic translation initiation factor 3 subunit A	132786	1126	34.46	37	13.06	up
A0A034VE18	Heat shock protein 23	18967	170	78.82	15	12.97	up
A0A034VR21	Protein bicaudal C	98705	899	26.25	15	12.51	up
A0A034VXK9	Aspartate aminotransferase (Fragment)	42034	376	76.86	26	12.49	up
A0A034VGT6	Eukaryotic translation initiation factor 5	51176	461	33.62	15	12.46	up
A0A034W5H1	60S ribosomal protein L23 (Fragment)	15903	149	65.77	13	12.44	up
A0A034VHY0	Uncharacterized protein (Fragment)	70311	662	26.89	10	12.33	up
A0A034VED9	UPF0586 protein CG11596	50004	435	13.1	6	12.01	up
A0A034VZV8	Chorion peroxidase	94126	836	40.31	29	12.00	up
A0A034V1Z6	Valine--tRNA ligase	120041	1054	39.75	36	11.94	up
A0A034VMA5	ATP-binding cassette sub-family F member 2	69942	610	27.7	17	11.71	up
A0A034VAV1	Proactivator polypeptide	107020	960	17.92	16	11.64	up
A0A034W4L6	Lipid storage droplets surface-binding protein 2	37989	353	61.47	20	11.61	up
A0A034VN85	Eukaryotic translation initiation factor 3 subunit E	50964	434	38.71	15	11.54	up
A0A034WBX6	Eukaryotic translation initiation factor 3 subunit L	63113	538	28.81	14	11.49	up
A0A034WLA5	ATP-dependent RNA helicase vasa, isoform A	67940	621	57.49	35	11.45	up
A0A034W0T2	Inosine-5'-monophosphate dehydrogenase	57498	534	35.96	12	11.42	up
A0A034W2M7	Elongation factor 1-gamma (Fragment)	49900	438	58.68	31	11.41	up
A0A034VAY0	40S ribosomal protein S3a (Fragment)	30469	269	71	24	11.40	up
A0A034V5W7	Methyltransferase-like protein 23	30225	276	18.48	5	11.34	up
A0A034VY08	S-adenosylmethionine synthase	44908	409	49.14	15	11.30	up
A0A034WL43	Putative arginine--tRNA ligase, cytoplasmic	76511	682	49.27	31	11.17	up
A0A034WB81	T-complex protein 1 subunit gamma	59276	543	71.27	41	10.98	up
A0A034WWD8	Exportin-1	123439	1070	40.56	37	10.85	up
A0A034W812	Importin subunit alpha	57164	520	63.65	22	10.77	up

A0A034V8K3	RING finger protein 17 (Fragment)	76371	675	34.81	17	10.68	up
A0A034WFM3	60S ribosomal protein L3	46663	413	50.36	26	10.66	up
A0A034WNG6	Alanine--tRNA ligase, cytoplasmic	108265	967	50.26	38	10.57	up
A0A034VX78	T-complex protein 1 subunit theta	59247	546	60.26	31	10.55	up
A0A034UZ33	Importin subunit beta	98887	885	37.51	26	10.39	up
A0A034WKN9	C2 domain-containing protein 3 (Fragment)	75399	682	33.43	14	10.37	up
A0A034WMJ8	40S ribosomal protein S4 (Fragment)	31529	280	59.29	23	10.35	up
A0A034WL96	60S ribosomal protein L13	25137	216	39.81	13	10.27	up
A0A034VA13	Eukaryotic initiation factor 4A	45755	403	67	28	10.20	up
A0A034W486	Protein transport protein Sec61 subunit alpha isoform 2 (Fragment)	37285	340	20.29	7	10.07	up
A0A034WNL1	ADP,ATP carrier protein	33080	302	73.51	27	10.05	up
A0A034WBZ8	Putative serine protease K12H4.7	59035	517	39.65	19	10.02	up
A0A034WJW9	26S proteasome non-ATPase regulatory subunit 5	56937	499	19.24	9	10.01	up
A0A034WT35	Uncharacterized protein	107757	923	22.1	17	10.01	up
A0A034VN45	60S ribosomal protein L4	46425	411	57.91	28	10.00	up
A0A034V6H0	Serine protease nudel (Fragment)	205363	1841	19.72	28	9.87	up
A0A034VXX3	Uncharacterized protein	38416	353	40.51	10	9.87	up
A0A034VCB2	60S ribosomal protein L12 (Fragment)	21090	196	52.55	8	9.85	up
A0A034VGJ1	Clustered mitochondria protein homolog	153417	1362	30.54	35	9.85	up
A0A034V813	Acetyl-CoA carboxylase	270348	2391	34.84	58	9.80	up
A0A034VUK5	Proliferation-associated protein 2G4	44175	403	79.9	29	9.71	up
A0A034WGZ4	Elongation factor Ts, mitochondrial	33818	305	40.66	9	9.68	up
A0A034VEP1	Exportin-5	43240	388	18.56	5	9.67	up
K9LCM4	Cytochrome P450 (Fragment)	26153	226	15.04	3	9.61	up
A0A034WRF7	D-3-phosphoglycerate dehydrogenase	35346	334	53.29	17	9.49	up
A0A034W7B3	Uncharacterized protein	45156	395	40.25	15	9.49	up
X2JR29	Ribosomal protein L19	24335	205	32.68	11	9.42	up
A0A034WNB0	26S proteasome non-ATPase regulatory subunit 12 (Fragment)	60837	531	33.71	21	9.38	up
A0A034WGD2	Ribosomal L1 domain-containing protein CG13096 (Fragment)	68771	608	32.07	18	9.37	up
A0A034VXE8	Uncharacterized protein	27095	242	35.12	6	9.33	up

A0A034WGD6	Protein zwilch	73614	646	18.27	10	9.31	up
A0A034VUT0	Protein singed (Fragment)	56975	509	31.43	13	9.29	up
A0A034VYI4	Tyrosine--tRNA ligase (Fragment)	58023	523	65.01	29	9.25	up
A0A034W3H8	T-complex protein 1 subunit epsilon	58973	540	61.67	28	9.24	up
A0A034VZW3	Transitional endoplasmic reticulum ATPase TER94	88721	799	65.08	52	9.20	up
A0A034VUQ7	Glucose-6-phosphate 1-dehydrogenase	60835	527	52.56	22	9.14	up
A0A034V9B8	Putative cytochrome P450 6a14	55942	490	49.8	22	9.10	up
A0A034WJ52	Eukaryotic translation initiation factor 4 gamma 1	195326	1766	36.98	46	9.09	up
A0A034WQL2	60S ribosomal protein L27a	17047	149	46.31	10	9.07	up
A0A034WG46	ATP synthase subunit b, mitochondrial	27344	240	47.08	15	9.03	up
A0A034VLP4	Transforming growth factor-beta-induced protein ig-h3	89994	810	44.69	28	9.02	up
A0A034VG22	RING finger protein 17 (Fragment)	60868	530	58.3	27	9.01	up
A0A034WIU2	Trypsin (Fragment)	25933	249	31.33	7	9.00	up
A0A034WCS4	Dehydrogenase/reductase SDR family member 11	27759	251	57.77	8	8.92	up
A0A034VH20	Piwi-like protein	102440	900	49.56	37	8.88	up
A0A034W6G9	Troponin C-akin-1 protein	18719	166	68.07	8	8.88	up
A0A034WQB6	Putative elongation factor 1-beta	24244	222	73.87	18	8.86	up
A0A034WVH2	Protein arginine N-methyltransferase 1	42277	366	51.37	18	8.79	up
A0A034W1W8	Aminoacyl tRNA synthase complex-interacting multifunctional protein 1	34730	318	32.7	9	8.62	up
A0A034V4Z0	Uncharacterized protein	213808	1884	10.35	18	8.62	up
A0A034W0G5	Eukaryotic translation initiation factor 3 subunit B	80496	689	42.24	28	8.55	up
A0A034W3A2	Serine--tRNA ligase, cytoplasmic	56415	497	49.09	17	8.51	up
A0A034WPR9	Asparagine synthetase	63212	557	11.85	6	8.49	up
A0A034WVX5	60S ribosomal protein L9	21321	190	60	9	8.38	up
A0A034V7V4	Uncharacterized protein (Fragment)	123947	1092	18.5	16	8.38	up
A0A034VC07	Thioredoxin reductase 1, mitochondrial	53451	493	71.4	28	8.14	up
A0A034VGU8	Eukaryotic translation initiation factor 2 subunit 3	51705	475	45.68	16	8.09	up
A0A034VLR1	40S ribosomal protein S10b (Fragment)	17968	159	58.49	15	8.04	up

A0A034W1V3	Diphthine synthase	31790	280	47.14	9	8.00	up
A0A034VUM4	Protein extra bases (Fragment)	48866	420	46.19	23	8.00	up
A0A034VS73	Transketolase-like protein 2 (Fragment)	47536	434	74.19	29	7.98	up
A0A034V4V7	HEAT repeat-containing protein 1-like protein (Fragment)	73677	651	11.67	7	7.94	up
A0A034WXR5	Ribosomal protein	24492	217	46.08	12	7.90	up
A0A034WEB5	T-complex protein 1 subunit alpha	59549	557	56.55	28	7.85	up
A0A034VDY8	Bifunctional glutamate/proline--tRNA ligase	185312	1636	48.66	65	7.80	up
A0A034WFZ2	Uncharacterized protein	29640	256	45.31	9	7.79	up
A0A034VPD5	Villin-like protein quail	103012	895	18.21	13	7.68	up
A0A034V085	Proteasome-associated protein ECM29-like protein	212089	1889	26.95	41	7.64	up
A0A034VUE9	40S ribosomal protein SA	30293	271	78.97	20	7.63	up
A0A034W0V2	Cystathionine beta-synthase	56287	515	13.4	6	7.60	up
A0A034VJX9	Mitochondrial-processing peptidase subunit beta	52467	472	58.47	22	7.59	up
A0A034WRN7	T-complex protein 1 subunit delta	57179	533	51.59	22	7.58	up
A0A034WAB1	Polyadenylate-binding protein	71344	649	63.17	38	7.52	up
A0A034VGS8	Tubulin beta-3 chain	50889	454	60.79	27	7.51	up
A0A034WL41	Eukaryotic translation initiation factor 3 subunit I	36174	326	58.28	14	7.49	up
A0A034WLZ1	Nascent polypeptide-associated complex subunit alpha (Fragment)	25683	238	38.66	12	7.45	up
A0A034VUA6	26S proteasome non-ATPase regulatory subunit 1	112769	1017	52.51	45	7.42	up
A0A034VPR3	Elongation factor Tu GTP-binding domain-containing protein 1	116814	1043	31.06	28	7.36	up
A0A034VRS1	Isocitrate dehydrogenase [NAD] subunit, mitochondrial	38533	354	56.78	19	7.35	up
A0A034V5U3	Staphylococcal nuclease domain-containing protein 1	103711	928	64.12	47	7.34	up
A0A034VRE9	40S ribosomal protein S15a	14758	130	60	9	7.27	up
A0A034VB23	RRP12-like protein	154813	1385	12.92	16	7.27	up
A0A034WQX9	Leucine-rich repeat-containing protein 59	35882	318	33.02	9	7.26	up

A0A034WMX5	60S ribosomal protein L44	12489	104	20.19	3	7.25	up
A0A034VHM0	cAMP-dependent protein kinase type I regulatory subunit	35904	322	35.09	9	7.24	up
A0A034VFE1	NADH dehydrogenase (Ubiquinone) iron-sulfur protein 3, mitochondrial	30290	264	59.47	14	7.24	up
A0A034V3P5	Uncharacterized protein (Fragment)	114127	1011	15.03	11	7.23	up
A0A034VX01	DNA-binding protein modulo (Fragment)	65113	578	37.89	22	7.20	up
A0A034V7H3	Niemann-Pick C1 protein (Fragment)	118447	1061	14.42	13	7.15	up
A0A034WTE1	Eukaryotic translation initiation factor 3 subunit M (Fragment)	46503	410	50.98	17	7.14	up
A0A034W9W1	Eukaryotic translation initiation factor 3 subunit H (Fragment)	38552	338	49.41	15	7.11	up
A0A034UZF8	Uncharacterized protein (Fragment)	150148	1341	6.26	8	7.11	up
A0A034VW82	1,4-alpha-glucan-branching enzyme	79824	689	47.61	26	6.99	up
A0A034WE93	Glycine--tRNA ligase	76602	679	50.07	31	6.98	up
A0A034VKS3	Threonine--tRNA ligase, cytoplasmic	79257	689	60.09	35	6.93	up
A0A034V173	Putative N-acetyltransferase san	21206	186	50.54	10	6.88	up
A0A034WPA4	28S ribosomal protein S35, mitochondrial	38291	332	31.02	8	6.87	up
A0A034W163	Xanthine dehydrogenase (Fragment)	143901	1298	19.95	21	6.86	up
A0A034UZI7	Midasin (Fragment)	430710	3762	6.01	20	6.76	up
A0A034VMS2	Peptide methionine sulfoxide reductase	27545	243	29.63	6	6.68	up
A0A034V1D4	CAD protein (Fragment)	111533	999	28.43	25	6.61	up
A0A034W5K7	Putative phenylalanine--tRNA ligase beta subunit	66264	591	43.82	21	6.61	up
A0A034VEZ1	Glutamate synthase (NADH), amyloplastic	230930	2093	43.91	77	6.50	up
A0A034WLE7	T-complex protein 1 subunit beta	57934	535	62.99	33	6.50	up
A0A034W9X8	E3 ubiquitin-protein ligase HECTD1	315089	2883	7.49	18	6.45	up
A0A034WAN3	Eukaryotic peptide chain release factor subunit 1	49005	437	29.98	12	6.45	up
A0A034WKN7	Methyltransferase-like protein 13	76261	675	33.63	19	6.45	up
A0A034WID4	Putative cation-transporting ATPase 13A1	136813	1216	18.67	19	6.44	up
A0A034W4W3	Transportin-1	100645	886	27.2	18	6.40	up

A0A034VMZ5	T-complex protein 1 subunit eta	59276	543	72.56	32	6.37	up
A0A034V225	Extracellular serine/threonine protein kinase FAM20C	61379	541	15.9	6	6.36	up
A0A034VA47	Uncharacterized protein (Fragment)	96787	853	21.81	18	6.36	up
A0A034VWI5	Leucine-rich PPR motif-containing protein, mitochondrial (Fragment)	159347	1418	43.86	50	6.35	up
A0A034WM27	Tubulin--tyrosine ligase-like protein 12	74892	641	29.49	17	6.28	up
A0A034V651	Sortilin-related receptor (Fragment)	61745	553	21.52	11	6.24	up
I6MC53	Heat shock protein 90	81750	715	55.8	49	6.23	up
A0A034WMH5	26S proteasome non-ATPase regulatory subunit 13	43727	382	47.64	20	6.22	up
A0A034WKN2	Putative aminoacyl tRNA synthase complex-interacting multifunctional protein 2	33483	296	38.85	11	6.20	up
A0A034VBA8	Isoleucine--tRNA ligase, cytoplasmic (Fragment)	53686	468	38.46	16	6.16	up
A0A034VG52	Adenosylhomocysteinase	56004	509	53.24	23	6.14	up
A0A034WM52	Cullin-associated NEDD8-dissociated protein 1	139107	1236	39.56	41	6.03	up
A0A034W3T2	Serine/threonine-protein phosphatase 5	61618	544	40.26	19	6.02	up
A0A034VRQ0	Translational activator GCN1 (Fragment)	161957	1471	34.81	39	5.98	up
A0A034V598	Coatomer subunit gamma	97386	877	47.09	29	5.96	up
A0A034VUY6	Methenyltetrahydrofolate synthase domain-containing protein	59463	538	56.51	32	5.91	up
A0A034WCI6	E3 UFM1-protein ligase 1-like protein (Fragment)	83853	744	34.01	22	5.90	up
A0A034VMW4	Uridine 5'-monophosphate synthase	53209	487	49.08	20	5.90	up
A0A034VRE6	Adenosylhomocysteinase	47406	431	39.91	19	5.89	up
A0A034VC08	Xaa-Pro dipeptidase	53654	480	42.29	18	5.88	up
A0A034WF61	Serine/threonine-protein phosphatase	34736	303	36.96	9	5.86	up
A0A034W9M9	Cytosolic 10-formyltetrahydrofolate dehydrogenase (Fragment)	25559	231	47.19	9	5.84	up
A0A034UZ41	Sepiapterin reductase	29503	263	50.57	10	5.82	up
A0A034VE03	ATP-binding cassette sub-family E member 1	69344	611	36.66	19	5.81	up

A0A034V3I0	CAD protein (Fragment)	136404	1234	20.83	17	5.81	up
A0A034W9S7	ATP synthase subunit O, mitochondrial (Fragment)	24333	225	68	18	5.75	up
A0A034WEU3	Enoyl-CoA delta isomerase 2, mitochondrial	28863	260	48.46	10	5.70	up
A0A034WFJ7	Putative elongator complex protein 1 (Fragment)	118063	1025	26.63	23	5.69	up
A0A034VAG4	Tudor domain-containing protein 12	96482	848	10.73	8	5.69	up
A0A034WUA5	Coatomer subunit beta	104950	931	50.7	34	5.67	up
A0A034VFZ9	Kinetochore-associated protein 1 (Fragment)	89215	770	26.36	17	5.67	up
A0A034WP91	NADH dehydrogenase (Ubiquinone) 1 alpha subcomplex subunit 8	19823	175	59.43	7	5.66	up
M1F4N9	Glutathione-s-transferase epsilon class 3	25749	228	48.25	11	5.64	up
A0A034WE30	Uncharacterized protein	43623	383	19.84	7	5.63	up
A0A034VLF2	Coatomer subunit beta	106993	953	33.47	26	5.60	up
A0A034V3E5	Nuclear pore complex protein Nup107 (Fragment)	103735	899	34.59	25	5.55	up
A0A034VAK1	Heat shock protein 75 kDa, mitochondrial	80438	710	42.25	25	5.53	up
A0A034WV61	Putative glutamine--tRNA ligase	89401	784	48.21	32	5.53	up
A0A034WNF6	Importin subunit alpha	57681	519	29.09	12	5.51	up
A0A034VER3	Eukaryotic translation initiation factor 3 subunit C	108335	923	37.92	32	5.49	up
A0A034WIL4	NADH dehydrogenase (Ubiquinone) iron-sulfur protein 2, mitochondrial	53289	473	43.97	18	5.45	up
A0A034W2L3	Citrate synthase (Fragment)	28581	257	63.42	15	5.37	up
A0A034V786	AP-3 complex subunit delta	116643	1033	8.03	8	5.36	up
A0A034VD95	39S ribosomal protein L1, mitochondrial	41222	358	19.27	6	5.34	up
A0A034VQT4	DNA topoisomerase 1	115728	1000	7.1	7	5.33	up
A0A034V9P5	Isoleucine--tRNA ligase, cytoplasmic (Fragment)	83274	719	49.65	29	5.33	up
A0A034WR13	39S ribosomal protein L23, mitochondrial	17960	150	30.67	5	5.32	up
A0A034VKN3	Leucine--tRNA ligase, cytoplasmic	135606	1182	45.18	44	5.32	up
A0A034VH70	La-related protein (Fragment)	132976	1206	24.71	20	5.31	up

A0A034WDL1	NADH-ubiquinone oxidoreductase 75 kDa subunit, mitochondrial	80086	736	46.47	29	5.25	up
A0A034WUW3	UPF0160 protein MYG1, mitochondrial (Fragment)	44465	385	37.14	12	5.24	up
A0A034VFR8	Glyoxylate reductase (Fragment)	30612	273	45.05	8	5.21	up
A0A034VH25	Alpha-tocopherol transfer-like protein	37515	324	28.4	10	5.20	up
A0A034V3F2	26S protease regulatory subunit 7	48544	433	62.82	30	5.19	up
A0A034VA48	Prolyl 4-hydroxylase subunit alpha-2	64303	561	23.53	9	5.19	up
A0A034W1C0	Eukaryotic translation initiation factor 4 gamma 2	106157	942	27.81	19	5.18	up
A0A034VKX9	Alkyldihydroxyacetonephosphate synthase (Fragment)	76813	689	22.35	11	5.12	up
A0A034W6X9	GTP-binding protein 128up	41005	368	52.45	16	5.12	up
A0A034VKY3	RuvB-like helicase 2	53267	476	36.34	14	5.07	up
A0A034VM32	CCR4-NOT transcription complex subunit 1 (Fragment)	123312	1091	20.99	20	5.06	up
A0A034VFI5	Centromere/kinetochore protein zw10	81461	708	17.94	11	5.03	up
A0A034W6I8	Exportin-2	109799	972	34.88	33	5.02	up
A0A034VRR7	E3 ubiquitin-protein ligase listerin	193519	1664	9.74	14	5.00	up
A0A034V2H4	Methionine--tRNA ligase, cytoplasmic	107507	969	41.49	29	4.97	up
A0A034VCV5	Nuclear cap-binding protein subunit 1	93543	801	26.34	16	4.96	up
A0A034WCI4	ATP synthase subunit alpha	59516	552	57.43	34	4.95	up
A0A034VFL7	Nucleolar protein 6	148323	1294	9.43	10	4.95	up
A0A034WU84	Proteasome subunit alpha type	28026	253	60.08	14	4.93	up
A0A034WIR4	DnaJ-like protein subfamily C member 2	72239	618	28.48	14	4.90	up
A0A034VJS5	Heat shock 70 kDa protein 14	57360	513	31.38	13	4.90	up
A0A034V3F4	Tripeptidyl-peptidase 2	149295	1353	47.97	54	4.90	up
A0A034VDF5	Developmentally-regulated GTP-binding protein 2	40576	363	50.14	14	4.89	up
A0A034V402	COP9 signalosome complex subunit 8	21657	184	21.2	3	4.88	up
A0A034WNZ1	Ubiquitin-conjugating enzyme E2 N	17208	151	52.32	8	4.88	up
A0A034W3L0	Ankyrin repeat and KH domain-containing protein mask (Fragment)	54726	507	29.98	11	4.84	up
A0A034WLF6	FK506-binding protein 59	48986	437	59.27	29	4.84	up
A0A034UZB5	Protein purity of essence (Fragment)	237724	2178	13.77	25	4.84	up

A0A034W9B6	T-complex protein 1 subunit zeta	58468	531	64.22	28	4.79	up
A0A034W590	Ubiquitin-like modifier-activating enzyme 1	133041	1204	58.89	53	4.79	up
A0A034VJH4	N-alpha-acetyltransferase 16, NatA auxiliary subunit	101634	878	33.94	28	4.76	up
D9YID1	Ribosomal protein S6 kinase	57202	507	11.05	5	4.76	up
A0A034V2H3	Protein purity of essence (Fragment)	104460	945	15.24	10	4.73	up
A0A034W6E5	Lysine--tRNA ligase	66232	584	42.12	23	4.72	up
A0A034WFC0	ATP synthase subunit gamma	33092	297	38.72	12	4.64	up
A0A034WRG8	Putative ribosomal RNA methyltransferase NOP2	93568	812	16.87	11	4.64	up
A0A034VAW1	Signal recognition particle subunit SRP68	69067	601	32.45	20	4.62	up
A0A034V6Q5	mRNA turnover protein 4-like protein	29891	257	28.79	8	4.61	up
A0A034W8Z3	Putative ATP-dependent RNA helicase DDX43	74677	662	47.28	27	4.60	up
A0A034VH61	Putative aminopeptidase W07G4.4	54904	505	59.8	27	4.58	up
A0A034W9F2	Histone-lysine N-methyltransferase setd3	61477	534	36.14	17	4.57	up
A0A034VYR3	Nucleolar protein 58	81673	728	43.82	30	4.56	up
A0A034V675	Unconventional myosin-Va (Fragment)	161865	1379	6.74	7	4.54	up
A0A034VP19	WD repeat-containing protein 36	104546	932	9.44	7	4.53	up
A0A034V1T1	Protein purity of essence (Fragment)	110879	976	15.16	12	4.50	up
A0A034VEG2	ER membrane protein complex subunit 1 (Fragment)	68095	611	24.39	11	4.49	up
A0A034WI55	Eukaryotic translation initiation factor 3 subunit D	63076	554	43.68	18	4.48	up
A0A034WNJ6	Heat shock 70 kDa protein cognate 5	73628	679	55.23	34	4.47	up
A0A034WL74	Midasin (Fragment)	124703	1102	8.89	9	4.44	up
A0A034VKY9	DnaJ-like protein subfamily C member 7	54472	474	24.05	10	4.40	up
A0A034W901	NADH dehydrogenase (Ubiquinone) 1 alpha subcomplex subunit 10, mitochondrial	46869	405	54.81	20	4.37	up
A0A034VRL9	H/ACA ribonucleoprotein complex subunit 4 (Fragment)	61846	547	25.59	13	4.36	up
A0A034WPS5	COP9 signalosome complex subunit 1b	56677	511	25.24	10	4.35	up
A0A034VFY5	Ras-related protein Rab-6A	23481	208	51.44	8	4.35	up

A0A034WPC8	Cysteine--tRNA ligase, cytoplasmic	84539	740	38.38	25	4.34	up
A0A034VEH7	Prefoldin subunit 4	15819	137	34.31	4	4.32	up
A0A034VMA3	L-threonine 3-dehydrogenase, mitochondrial	41242	365	17.53	5	4.31	up
A0A034WVA3	Elongation factor Tu	53920	490	49.39	19	4.26	up
A0A034W692	Putative ATP-dependent RNA helicase DDX20 (Fragment)	42397	381	50.92	16	4.26	up
A0A034W1G8	Transcription elongation regulator 1 (Fragment)	92302	765	10.85	8	4.25	up
A0A034VBM4	Trifunctional purine biosynthetic protein adenosine-3	148058	1368	35.75	39	4.25	up
A0A034VN48	Putative fatty acyl-CoA reductase CG5065	64021	566	12.19	7	4.23	up
A0A034WRK8	Farnesyl pyrophosphate synthase	46845	408	17.16	6	4.21	up
A0A034VA86	116 kDa U5 small nuclear ribonucleoprotein component	110665	975	26.46	22	4.18	up
A0A034V6V7	Coatomer subunit alpha	139329	1233	38.04	36	4.18	up
A0A034WJ65	Vigilin	142149	1278	43.27	45	4.18	up
A0A034W550	Phospholipase A-2-activating protein	85785	787	43.58	24	4.17	up
A0A034V9G7	Eukaryotic translation initiation factor 5B	134978	1215	24.86	20	4.16	up
A0A034V3U3	Mitochondrial import receptor subunit TOM22-like protein	16705	150	51.33	7	4.15	up
A0A034VGJ0	Putative multidrug resistance-associated protein lethal(2)03659	57681	513	21.64	9	4.15	up
A0A034WIJ6	Transducin beta-like protein 3 (Fragment)	83189	750	10.4	6	4.12	up
A0A034V083	Uncharacterized protein	33332	295	43.73	8	4.11	up
A0A034V540	Uncharacterized protein	28794	253	43.08	9	4.10	up
A0A034WSI9	Glutathione S-transferase 1	25141	221	33.03	9	4.07	up
A0A034VX54	Aspartate aminotransferase, cytoplasmic	45679	407	60.69	20	4.06	up
A0A034VV21	Nuclear pore complex protein Nup205 (Fragment)	124867	1088	22.79	20	4.06	up
A0A034V2I5	Putative ATP-dependent RNA helicase DDX17 (Fragment)	57084	523	40.34	18	4.06	up
A0A034WRS4	60S acidic ribosomal protein P2	11743	113	74.34	9	4.04	up

A0A034WIU7	Tetratricopeptide repeat protein 27	91135	792	16.67	11	3.99	up
A0A034WVQ7	Nodal modulator 1	135226	1207	22.37	20	3.98	up
A0A034WHJ1	Histidine--tRNA ligase, cytoplasmic	56910	512	50.59	22	3.97	up
A0A034VNJ3	Proline dehydrogenase 1, mitochondrial	80238	704	23.72	13	3.97	up
A0A034V6T2	Insulin-degrading enzyme	120515	1048	24.43	24	3.94	up
A0A034VZH7	26S proteasome non-ATPase regulatory subunit 2	101079	911	48.74	35	3.93	up
A0A034WSY7	Nucleolar GTP-binding protein 1	75319	651	23.04	13	3.91	up
A0A034WDU4	Endoplasmin	91351	797	56.21	46	3.90	up
A0A034WNJ2	Signal recognition particle subunit SRP72	73751	654	43.58	24	3.90	up
A0A034WMW7	Putative elongator complex protein 4	51886	463	29.81	9	3.89	up
A0A034WU78	Fumarate hydratase, mitochondrial	54067	500	48	18	3.87	up
A0A034WGZ6	40S ribosomal protein S7 (Fragment)	16318	141	49.65	10	3.84	up
A0A034VAS4	Acetyltransferase component of pyruvate dehydrogenase complex	53683	503	39.36	14	3.79	up
A0A034WK84	Proteasome subunit beta type	29551	265	44.53	12	3.79	up
A0A034V4B4	Maternal protein tudor	303712	2704	25.89	58	3.77	up
A0A034WAN7	Signal recognition particle 54 kDa protein	55927	508	36.22	12	3.76	up
A0A034W710	Ankyrin repeat and KH domain-containing protein mask (Fragment)	85099	788	10.79	5	3.71	up
A0A034WRK0	Mitochondrial import receptor subunit TOM70	66886	591	40.61	21	3.70	up
A0A034W1H3	Nuclear pore complex protein Nup93	92985	809	38.32	27	3.68	up
A0A034W0P2	Tudor domain-containing protein 1	75457	653	16.69	10	3.68	up
A0A034V250	Glycogen debranching enzyme	210785	1878	18.69	32	3.67	up
A0A034V087	Guanine nucleotide-binding-like protein 3-like protein (Fragment)	69470	610	29.02	16	3.67	up
A0A034V4D6	DnaJ-like protein subfamily C member 13 (Fragment)	220756	1936	21.02	31	3.66	up
A0A034VW24	NADH dehydrogenase (Ubiquinone) 1 alpha subcomplex subunit 9, mitochondrial	47184	412	39.08	16	3.65	up
A0A034VEP3	4-aminobutyrate aminotransferase, mitochondrial	54817	491	48.88	18	3.64	up
A0A034VF36	Ubiquitin-conjugating enzyme E2 Q2	43953	389	9.77	3	3.63	up

A0A034VST3	Nicalin-1	62816	561	23.71	10	3.61	up
A0A034V7N4	Myosin heavy chain 95F	141826	1240	28.63	32	3.60	up
A0A034WCY3	Dedicator of cytokinesis protein 3 (Fragment)	204946	1790	5.81	9	3.56	up
A0A034VIU6	ATP synthase-coupling factor 6, mitochondrial	11877	105	39.05	6	3.50	up
A0A034VFE4	DDB1-and CUL4-associated factor-like 1 (Fragment)	106321	962	12.89	9	3.50	up
A0A034VY24	ATP-dependent RNA helicase Ddx1 (Fragment)	84144	750	41.33	27	3.48	up
A0A034WGV9	Tryptophan--tRNA ligase, cytoplasmic	48047	428	29.44	11	3.46	up
A0A034VCY4	Tudor domain-containing protein 7 (Fragment)	108951	956	25.63	18	3.41	up
A0A034WNV2	39S ribosomal protein L3, mitochondrial	42074	370	24.32	8	3.40	up
A0A034WPQ1	DnaJ-like protein subfamily A member 1	45576	402	55.72	22	3.40	up
A0A034VNQ2	Nuclear pore complex protein Nup85	77843	677	36.63	19	3.38	up
A0A034WJS4	Protein l(2)37Cc	30367	276	85.14	21	3.38	up
A0A034WBB3	Serine-threonine kinase receptor-associated protein	32672	295	48.14	10	3.38	up
A0A034WTU5	39S ribosomal protein L12, mitochondrial	19776	183	24.04	2	3.37	up
A0A034VDF3	Squamous cell carcinoma antigen recognized by T-cells 3 (Fragment)	61804	532	31.2	17	3.34	up
A0A034VXN0	Short/branched chain specific acyl-CoA dehydrogenase, mitochondrial	45479	413	41.16	12	3.30	up
A0A034W308	Bifunctional 3'-phosphoadenosine 5'-phosphosulfate synthase	70060	625	38.56	17	3.29	up
A0A034WWN2	DEAD-box helicase Dbp80	51990	463	59.83	22	3.29	up
A0A034V555	Ubiquitin carboxyl-terminal hydrolase (Fragment)	138806	1221	14.58	15	3.28	up
A0A034W1I4	Exportin-5 (Fragment)	95335	841	14.63	11	3.26	up
A0A034W705	Ras-related protein Rab-2A	23664	213	66.2	12	3.26	up
A0A034WMK4	Zinc finger protein ZPR1	51616	457	23.85	10	3.25	up
A0A034W5W7	Kelch domain-containing protein 4	57367	514	15.76	7	3.24	up
A0A034VQ24	60S ribosomal export protein NMD3	59124	517	30.95	12	3.23	up

A0A034VFS4	Glutamate-rich WD repeat-containing protein 1	52062	465	18.49	7	3.23	up
A0A034VG90	Nuclear pore complex protein Nup133	135450	1193	20.12	19	3.23	up
A0A034WCL6	Peptidyl-prolyl cis-trans isomerase FKBP6 (Fragment)	54511	478	43.93	16	3.22	up
A0A034VMD8	Cytoskeleton-associated protein 5 (Fragment)	135423	1227	26.57	26	3.15	up
A0A034WQD6	Armadillo repeat-containing protein 8	74696	664	15.21	9	3.14	up
A0A034W6W9	Translocation protein SEC63-like protein	86851	759	15.68	10	3.11	up
A0A034WIS7	NADH dehydrogenase (Ubiquinone) flavoprotein 1, mitochondrial (Fragment)	52627	480	44.37	17	3.10	up
A0A034WSC9	Putative apoptosis-inducing factor 1, mitochondrial	74598	685	29.78	13	3.09	up
A0A034W273	Aspartate--tRNA ligase, cytoplasmic	60020	539	49.91	25	3.07	up
A0A034WMX6	Putative prefoldin subunit 5	18487	160	76.88	10	3.07	up
A0A034WCH5	Obg-like ATPase 1	44839	397	53.9	16	3.06	up
A0A034WJH2	Protein dpy-30-like protein	10966	99	47.47	4	3.05	up
A0A034VFT5	tRNA (Cytosine(34)-C(5))-methyltransferase	82282	728	33.52	16	3.05	up
A0A034VFV1	Cytosolic 10-formyltetrahydrofolate dehydrogenase (Fragment)	75060	687	47.02	23	3.04	up
A0A034WBD3	Protein TBRG4	63632	581	18.76	8	3.02	up
A0A034VRI8	E3 ubiquitin-protein ligase hyd (Fragment)	266419	2371	11.43	24	2.95	up
A0A034W9S5	Glycylpeptide N-tetradecanoyltransferase	54261	475	41.89	18	2.95	up
A0A034VJX4	Very long-chain-fatty-acid--CoA ligase bubblegum	74013	666	30.33	15	2.95	up
A0A034VS83	Hypoxia up-regulated protein 1	107546	957	42.11	30	2.93	up
A0A034W954	Protein RRP5-like protein	163045	1433	17.52	19	2.93	up
A0A034WCD3	NEDD8-activating enzyme E1 regulatory subunit	58530	522	29.12	13	2.92	up
A0A034WAX6	Oligopeptidase A	81683	724	43.09	26	2.84	up
A0A034WPD8	Putative rRNA methyltransferase	93409	814	10.44	8	2.83	up
A0A034VW78	Protein misato	64326	564	12.41	5	2.81	up

A0A034WG96	Putative elongator complex protein 2	93814	830	17.35	11	2.79	up
A0A034WBU6	Putative 39S ribosomal protein L24, mitochondrial	29141	248	37.1	7	2.75	up
A0A034W4A7	Glutathione S-transferase 1-1	23716	207	49.76	11	2.74	up
A0A034VMX3	NADH dehydrogenase (Ubiquinone) flavoprotein 2, mitochondrial	26900	243	53.09	10	2.74	up
A0A034VZ68	Protein arginine N-methyltransferase	72002	624	20.35	10	2.71	up
A0A034VF44	WD repeat-containing protein mio (Fragment)	69582	613	15.33	8	2.70	up
A0A034W4T9	Ubiquitin carboxyl-terminal hydrolase	110903	1010	16.53	11	2.67	up
A0A034VCR1	Putative glycogen (Starch) synthase	80869	702	25.21	14	2.64	up
A0A034VVK7	Chaperone activity of bc1 complex-like, mitochondrial	78284	701	24.39	13	2.62	up
A0A034WPC0	D-arabinose 1-dehydrogenase	39068	346	36.99	12	2.61	up
A0A034WKD6	Activator of 90 kDa heat shock protein ATPase-like protein 1	40213	353	44.19	15	2.58	up
A0A034W8R2	Ribosomal RNA processing protein 1-like protein	81414	704	13.49	8	2.58	up
A0A034VTV6	Glutaredoxin-related protein 5, mitochondrial	18955	169	29.59	4	2.56	up
A0A034WKL8	Putative rRNA-processing protein EBP2-like protein	35357	307	15.96	4	2.54	up
A0A034V8U5	Putative RNA helicase armi	139061	1208	13.91	15	2.53	up
A0A034WSS0	Protein unc-45-like protein B	105514	943	14.1	11	2.50	up
A0A034WE70	Eukaryotic translation initiation factor 3 subunit F	31217	280	65.36	13	2.49	up
A0A034WMK1	DNA-directed RNA polymerase	186312	1649	16.43	20	2.42	up
A0A034V222	39S ribosomal protein L16, mitochondrial	21943	189	32.8	5	2.40	up
A0A034VSP0	Phagocyte signaling-impaired protein	114527	989	12.44	12	2.30	up
A0A034V7P1	DNA polymerase	125024	1098	19.95	19	2.27	up
A0A034WSK7	Signal recognition particle receptor subunit alpha-like protein (Fragment)	66631	597	34.34	18	2.24	up
A0A034W887	Prolactin regulatory element-binding protein	49585	448	23.44	8	2.13	up
A0A034W439	Ubiquitin carboxyl-terminal hydrolase	90946	809	43.39	26	2.07	up

A0A034WYF0	Peroxiredoxin-2	26204	233	51.93	11	2.03	up
A0A034V9S0	Large subunit GTPase 1-like protein	73159	641	23.87	12	1.92	up
A0A034WAD0	Uncharacterized protein	14800	150	80	8	0.58	down
A0A034VRI7	Alpha-actinin, sarcomeric	103820	895	62.35	53	0.55	down
A0A034WIC2	Putative alpha-aspartyl dipeptidase	27043	240	33.33	7	0.55	down
A0A034VPM4	Vinculin	106095	960	50.63	38	0.54	down
A0A034VKE6	Bifunctional methylenetetrahydrofolate dehydrogenase/cyclohydrolase, mitochondrial	34038	316	32.59	8	0.50	down
A0A034VXI2	Aldehyde dehydrogenase family 3 member B1	62405	559	35.24	17	0.49	down
A0A034VUR2	Protein disulfide-isomerase A5	195906	1693	4.13	6	0.49	down
A0A034WHJ3	Uncharacterized protein	276170	2345	24.18	52	0.49	down
A0A034WDJ1	CD109 antigen	165284	1472	15.35	21	0.48	down
A0A034WAL2	Histone H1, gonadal	23448	215	31.16	6	0.47	down
A0A034W374	Metal transporter CNNM2	105061	947	19.54	14	0.47	down
A0A034VQG5	Nucleoprotein TPR	271019	2410	26.22	53	0.47	down
A0A034VK06	Dystonin (Fragment)	566833	4968	12.32	52	0.46	down
A0A034VFW9	Uncharacterized protein (Fragment)	50113	443	24.83	7	0.46	down
A0A034WQZ8	Zinc carboxypeptidase A 1	53165	464	49.35	16	0.46	down
A0A034VYI5	Non-specific protein-tyrosine kinase	58701	510	27.25	12	0.45	down
A0A034VW38	SUN domain-containing protein 1 (Fragment)	67523	594	21.55	13	0.45	down
A0A034V5V1	ZNF domain containing Pogo transposable element	147005	1326	16.14	18	0.43	down
A0A034WSJ2	Membrane-associated progesterone receptor component 2	25529	231	40.26	8	0.42	down
A0A034WSP8	Tumor protein D54	39216	351	43.3	13	0.42	down
A0A034VNQ6	UBX domain-containing protein 1	35187	316	31.65	9	0.42	down
A0A034WBM1	Acetyl-CoA acetyltransferase, cytosolic	41553	394	41.62	11	0.41	down
A0A034VU54	Neuroserpin (Fragment)	50223	451	37.47	14	0.41	down
A0A034WG74	Beta-galactosidase	72023	639	28.48	13	0.40	down
A0A034UZL6	Vacuolar protein sorting-associated protein 13A (Fragment)	293380	2604	23.85	49	0.40	down
A0A034WM87	Glutamine synthetase	44947	402	29.35	8	0.39	down

A0A034V413	Nesprin-1	915393	7973	19.68	119	0.39	down
A0A034VI64	Carboxypeptidase	55213	483	44.72	25	0.38	down
A0A034WAI7	Non-specific serine/threonine protein kinase	64369	571	16.81	7	0.38	down
A0A034WQQ0	Calcyphosin-like protein	24650	219	27.4	6	0.37	down
A0A034VE56	Galectin	60787	532	12.78	7	0.37	down
A0A034WSZ7	Heterochromatin protein 1	23688	212	23.11	5	0.37	down
A0A034W5F4	Uncharacterized protein	28374	246	26.02	7	0.37	down
A0A034V6L8	Filamin-B	87995	815	5.15	4	0.36	down
A0A034V3R0	Papilin	333708	3093	19.92	49	0.36	down
A0A034VM61	Clavesin-2	36462	320	33.44	9	0.35	down
A0A034VUS9	Dihydropyrimidine dehydrogenase (NADP(+))	113562	1047	5.83	5	0.35	down
A0A034VDX0	L-2-hydroxyglutarate dehydrogenase, mitochondrial	50614	455	31.21	10	0.35	down
A0A034VPR7	Putative peptidyl-prolyl cis-trans isomerase dodo	17917	160	57.5	11	0.35	down
A0A034VS34	Uncharacterized protein	90376	818	4.28	3	0.35	down
A0A034WLM6	Chromosomal protein D1	23347	211	30.33	4	0.34	down
M1F3Z9	Glutathione S-transferase omega-1	29161	255	46.67	11	0.34	down
A0A034WN98	17-beta-hydroxysteroid dehydrogenase 13	36367	326	25.15	7	0.33	down
A0A034V7D4	Microsomal triglyceride transfer protein large subunit (Fragment)	82310	737	34.6	24	0.33	down
A0A034V7N7	Protein lethal(2) giant larvae	127930	1176	24.23	21	0.33	down
A0A034VZK0	Muscle M-line assembly protein unc-89	372226	3274	12.43	33	0.32	down
A0A034WJT9	Chitooligosaccharidolytic beta-N-acetylglucosaminidase (Fragment)	51218	451	16.63	6	0.31	down
A0A034VG51	Uncharacterized protein (Fragment)	67454	610	9.67	6	0.31	down
A0A034V8X4	Alcohol dehydrogenase (NADP(+)) A	39659	350	40	11	0.30	down
A0A034W4V5	DE-cadherin	178717	1580	14.75	22	0.30	down
A0A034VTK4	DNA topoisomerase 2	167839	1481	42.13	59	0.30	down
A0A034WS42	Histone H4	11382	103	65.05	15	0.30	down
A0A034W2B1	Protein peanut	59357	526	21.86	11	0.30	down
A0A034WF58	Glutathione peroxidase	30583	278	48.92	13	0.29	down

A0A034WML8	Protein takeout	30874	269	20.07	4	0.29	down
A0A034WT25	Uncharacterized protein	31077	274	43.8	8	0.29	down
A0A034V3G3	Uncharacterized protein (Fragment)	45689	397	24.18	7	0.29	down
A0A034VRQ6	Protein held out wings	39076	356	37.64	11	0.28	down
A0A034W4Y6	Pyruvate carboxylase	130974	1181	47.59	45	0.28	down
A0A034VKY5	Uncharacterized protein	112036	1045	9.09	7	0.28	down
A0A034V2B1	Uncharacterized protein (Fragment)	199309	1825	7.18	10	0.28	down
A0A034VLS7	V-type proton ATPase subunit a	94994	834	18.11	15	0.28	down
A0A034VI38	Lysosomal acid phosphatase	55250	477	33.75	12	0.27	down
A0A034WB29	Cytoplasmic protein NCK1	62548	564	12.06	6	0.26	down
A0A034V4T6	Fasciclin-2 (Fragment)	57857	520	45.19	17	0.26	down
A0A034VAC6	Protein elav	47180	446	35.65	12	0.26	down
A0A034VVJ5	V-type proton ATPase subunit E	26077	226	63.27	13	0.26	down
A0A034VUP0	Chitinase-like protein Idgf1 (Fragment)	53054	471	15.92	6	0.25	down
A0A034V1H8	Filamin-A (Fragment)	141569	1301	54.04	58	0.25	down
A0A034VWH3	Myosin regulatory light chain 2	23610	222	52.7	10	0.25	down
A0A034W3K4	Myosin-IB	117898	1025	10.15	10	0.25	down
A0A034W317	Uncharacterized protein	84817	798	20.93	14	0.25	down
A0A034WRZ0	Leucine-rich repeat transmembrane neuronal protein 2 (Fragment)	52267	472	15.25	6	0.24	down
A0A034W6C4	Ubiquitin-conjugating enzyme E2 H	20867	183	39.34	7	0.24	down
A0A034V975	Uncharacterized protein	75107	674	30.42	18	0.24	down
A0A034WT83	Alpha-tocopherol transfer-like protein	33631	287	19.86	4	0.23	down
A0A034WWI9	Ejaculatory bulb-specific protein 3	14702	127	22.05	3	0.23	down
A0A034VPA5	Heterogeneous nuclear ribonucleoprotein Q (Fragment)	47111	417	21.34	9	0.23	down
A0A034WL14	RING finger protein unkempt (Fragment)	69020	604	12.91	5	0.23	down
A0A034UX56	Trehalose 6-phosphate phosphatase	31189	274	81.75	25	0.23	down
A0A034VE41	Twitchin (Fragment)	556499	4978	23.54	86	0.23	down
A0A034WR33	Copper homeostasis protein cutC-like protein	27453	256	18.75	4	0.22	down
A0A034V1F4	Low-density lipoprotein receptor-related protein 2 (Fragment)	187971	1677	7.39	9	0.22	down
A0A034VDP0	Obscurin (Fragment)	70619	631	30.74	13	0.22	down
A0A034W8V0	Otefin	44986	402	29.6	9	0.22	down

A0A034UZS9	PDZ and LIM domain protein Zasp	52978	495	22.22	9	0.22	down
A0A034VZ39	Peroxidasin	136185	1202	18.89	19	0.22	down
A0A034V2K7	Spectrin beta chain, non-erythrocytic 5	483273	4200	26.48	94	0.22	down
A0A034VR92	Tropomyosin-2	32743	284	57.04	17	0.22	down
A0A034V520	Uncharacterized protein	65301	610	16.89	10	0.22	down
A0A034WJW3	Uncharacterized protein	34599	300	18.33	5	0.22	down
A0A034V6M3	60S ribosomal protein L23a	28521	266	26.32	10	0.21	down
A0A034V322	CD109 antigen	164963	1472	4.82	6	0.21	down
A0A034VJR8	Endochitinase (Fragment)	69901	617	7.78	4	0.21	down
A0A034W8D6	Ferritin	25119	223	52.47	9	0.21	down
Q86QQ0	Glutathione S-transferase	23756	209	37.32	6	0.21	down
A0A034VSX0	Phosphate-regulating neutral endopeptidase	79759	683	22.69	13	0.21	down
A0A034UZB9	Sushi, von Willebrand factor type A, EGF and pentraxin domain-containing protein 1 (Fragment)	127179	1152	7.46	6	0.21	down
A0A034V5Q6	Talin-1 (Fragment)	82628	783	31.67	17	0.21	down
A0A034VND6	Tropomyosin-1, isoforms 33/34 (Fragment)	32457	282	48.58	20	0.21	down
A0A034VRE0	Angiotensin-converting enzyme	71478	617	6.48	4	0.20	down
A0A034VU99	Glutathione S-transferase S1	26938	240	65.83	17	0.20	down
A0A034WSM6	Guanine nucleotide-binding protein G(S) subunit alpha	44569	382	32.2	10	0.20	down
A0A034VII9	Inhibitor of growth protein	44693	413	13.56	4	0.20	down
A0A034WF82	Neurotrimin	61287	550	9.45	5	0.20	down
A0A034VP83	Poly(U)-specific endoribonuclease-like protein	71754	675	24.74	13	0.20	down
A0A034WJD1	3-ketoacyl-CoA thiolase, mitochondrial	41809	398	63.07	21	0.19	down
A0A034V2V8	5-formyltetrahydrofolate cyclo-ligase	22678	201	29.85	5	0.19	down
A0A034W1X2	Alaserpin	44489	398	27.39	10	0.19	down
A0A034WRQ2	Alpha-parvin	42015	366	33.06	10	0.19	down
A0A034W3G0	Chitinase-like protein Idgf4	48370	439	68.56	20	0.19	down
A0A034W8L1	Flotillin-2	46568	425	43.53	17	0.19	down
A0A034VY88	Protein hu-li tai shao	76611	698	69.63	35	0.19	down
A0A034VIH5	Protein lap4 (Fragment)	83204	783	17.5	9	0.19	down

A0A034VQR8	Troponin T, skeletal muscle	45988	384	39.32	22	0.19	down
A0A034VW95	Alaserpin (Fragment)	35699	321	31.46	9	0.18	down
A0A034W4R7	Alpha,alpha-trehalose-phosphate synthase (UDP-forming) A	91999	813	33.46	24	0.18	down
A0A034WI29	Protein lin-7-like protein B	21479	195	28.72	5	0.18	down
A0A034VVP8	Serpin B9	56716	504	29.76	14	0.18	down
A0A034V946	Src substrate cortactin (Fragment)	67412	601	16.97	9	0.18	down
A0A034W6I6	Amidophosphoribosyltransferase	57877	532	14.85	7	0.17	down
A0A034WI17	ATP-binding cassette sub-family A member 3	36412	320	15.31	4	0.17	down
A0A034W8P6	Protein E(Sev)2B	24462	211	43.6	9	0.17	down
A0A034WR79	Uncharacterized protein	24755	214	59.35	13	0.17	down
A0A034VTK3	Chitinase-like protein Idgf5	48605	432	27.31	9	0.16	down
A0A034VAR1	Clavesin-1	33921	293	29.35	8	0.16	down
A0A034VDB5	Protein lap4 (Fragment)	101687	915	20.11	14	0.16	down
A0A034WT05	Uncharacterized protein (Fragment)	26868	237	16.46	3	0.16	down
A0A034VQ98	Arylphorin subunit C223 (Fragment)	47837	407	1.97	1	0.15	down
A0A034W4H4	Chitinase-like protein Idgf2	49041	438	41.55	14	0.15	down
A0A034WM22	Multiple inositol polyphosphate phosphatase 1	54124	467	13.7	6	0.15	down
A0A034VKX8	Phosphatidylinositol 4-phosphate 5-kinase type-1 alpha	86262	777	6.82	4	0.15	down
A0A034V6W2	Short-chain dehydrogenase/reductase family 16C member 6	40762	376	35.11	10	0.15	down
A0A034V5R2	Talin-1 (Fragment)	211049	1928	48.86	69	0.15	down
A0A034VLD0	Xaa-Pro aminopeptidase 2	80936	704	25	19	0.15	down
A0A034VQZ0	Zinc finger protein 512B	44855	427	26.23	7	0.15	down
A0A034WGA0	Kin of IRRE-like protein 2 (Fragment)	68865	620	5.64	4	0.14	down
A0A034WBA7	Putative G-protein coupled receptor 158	94535	809	29.05	20	0.14	down
A0A034WG07	Uncharacterized protein	25352	222	31.98	7	0.14	down
A0A034W0B4	Chitinase-like protein CG5210 (Fragment)	31320	279	51.25	14	0.13	down
A0A034W237	Esterase B1	64331	566	14.49	6	0.13	down
A0A034VJ94	Glucose dehydrogenase (Acceptor)	68918	620	11.77	6	0.13	down

A0A034V5I2	Muscle M-line assembly protein unc-89 (Fragment)	213521	1922	17.9	27	0.13	down
A0A034WJ51	Nucleosomal histone kinase 1	70264	637	13.66	7	0.13	down
A0A034V5Q1	Ras suppressor protein 1 (Fragment)	15927	138	39.86	5	0.13	down
A0A034VA84	Uncharacterized protein	57231	502	22.91	10	0.13	down
A0A034VBU4	Uncharacterized protein (Fragment)	72518	648	11.57	7	0.13	down
A0A034VKK9	Alcohol dehydrogenase (NADP(+))	36548	324	38.89	13	0.12	down
A0A034V1E0	Cell surface glycoprotein 1	139929	1257	15.27	17	0.12	down
A0A034VYW9	Contactin	158548	1393	25.2	27	0.12	down
A0A034V9E0	Gelsolin	88396	789	17.36	13	0.12	down
A0A034VUS5	Integrin-linked protein kinase	50786	448	32.37	14	0.12	down
A0A034WVT6	Membrane metallo-endopeptidase-like 1 (Fragment)	82524	714	22.13	14	0.12	down
A0A034WC26	Sarcotoxin-2A	31053	301	72.76	16	0.12	down
A0A034VCQ3	Uncharacterized protein	127687	1161	6.46	7	0.12	down
A0A034WGR6	Uncharacterized protein	26306	231	38.53	6	0.12	down
A0A034V2S6	Uncharacterized protein	105991	944	10.7	7	0.12	down
A0A034V6M6	Antithrombin-III	47900	422	41.47	17	0.11	down
A0A034WAX0	Collagen alpha-1(XV) chain	124401	1184	8.53	10	0.11	down
A0A034VC57	Peroxidasin-like protein	155580	1384	11.49	13	0.11	down
A0A034VI82	Tropomodulin	43782	386	34.97	10	0.11	down
A0A034VQI8	Uncharacterized protein (Fragment)	72191	683	33.82	17	0.11	down
A0A034V3K5	CUB and sushi domain-containing protein 1 (Fragment)	96461	887	14.99	10	0.10	down
A0A034VHH3	Laminin subunit alpha-1 (Fragment)	188941	1677	10.26	14	0.10	down
A0A034VW09	Protein Skeletor, isoforms B/C	82875	741	29.82	14	0.10	down
A0A034V7Q2	Protein yellow (Fragment)	29702	261	31.42	6	0.10	down
A0A034W3W2	Regucalcin	32384	296	42.91	12	0.10	down
A0A034VEP9	Tenascin-R (Fragment)	29717	257	14.79	3	0.10	down
A0A034VE17	Uncharacterized protein	27887	240	26.67	6	0.10	down
A0A034VIV7	Uncharacterized protein (Fragment)	15284	132	12.88	2	0.10	down
A0A034V8W7	Cytochrome P450 4g15	55074	484	23.76	10	0.09	down
A0A034VCC3	Filamin-B (Fragment)	124654	1130	5.93	7	0.09	down
A0A034VIW3	Homer protein-like protein 2	42349	389	19.02	6	0.09	down
A0A034WSV2	Lamin-C	70077	622	56.27	37	0.09	down

A0A034V2U0	Protein 4.1-like protein	185190	1703	57.37	82	0.09	down
A0A034W6R3	Tissue inhibitor of metalloproteases	23428	210	15.71	5	0.09	down
A0A034VPE5	Tropomyosin-1, isoforms 9A/A/B (Fragment)	21903	188	69.68	19	0.09	down
A0A034VP05	Uncharacterized protein	103708	904	6.42	6	0.09	down
A0A034WAU8	Uncharacterized protein	73989	755	7.81	4	0.09	down
A0A034WH51	Uncharacterized protein	52329	462	22.51	10	0.09	down
A0A034VXK4	Uncharacterized protein (Fragment)	44748	431	59.63	16	0.09	down
A0A034VUV3	Lamin Dm0	69746	617	71.64	46	0.08	down
A0A034W3F0	Microtubule-associated protein	41229	389	20.05	4	0.08	down
A0A034V2D5	Neurogenic locus notch-like protein 1 (Fragment)	446774	4167	1.46	5	0.08	down
A0A034VJG4	Neurotactin	95358	871	9.87	6	0.08	down
A0A034VWT9	Putative cytochrome P450 309a1	58142	502	13.15	6	0.08	down
A0A034WF27	TPPP family protein CG4893	21493	201	40.3	7	0.08	down
A0A034VSB5	Fasciclin-1	61398	542	35.42	17	0.07	down
A0A034WRI9	Sarcocystatin-A	13543	125	47.2	6	0.07	down
A0A034VZZ4	Uncharacterized protein	16306	145	17.24	2	0.07	down
A0A034VAC8	Uncharacterized protein	95118	885	8.93	8	0.07	down
A0A034WHZ8	Uncharacterized protein (Fragment)	53199	464	21.77	12	0.07	down
A0A034W3I2	Zinc finger protein 512B	44649	414	11.59	3	0.07	down
A0A034WCQ3	Carbonic anhydrase 15 (Fragment)	39936	350	9.71	3	0.06	down
A0A034VC00	Collagen alpha-1(IV) chain	185541	1833	13.04	21	0.06	down
A0A034WXP8	Uncharacterized protein	16783	153	9.15	2	0.06	down
A0A034VT34	Angiopoietin-4	52090	454	20.04	7	0.05	down
A0A034WHU4	Delta-1-pyrroline-5-carboxylate synthase	83870	773	19.79	13	0.05	down
A0A034VWI9	General odorant-binding protein 99a (Fragment)	20372	176	18.18	4	0.05	down
I1T1H2	Heat shock protein 20	18932	170	67.65	10	0.05	down
A0A034W9Y1	Larval cuticle protein A2B	24482	250	92.4	19	0.05	down
A0A034W4Q0	Larval cuticle protein A2B (Fragment)	18617	180	73.89	17	0.05	down
A0A034VU29	Regucalcin	39454	351	64.96	26	0.05	down
A0A034VGV7	Uncharacterized protein	89626	838	31.5	19	0.05	down
A0A034VSZ7	Uncharacterized protein	26073	229	47.16	13	0.05	down
A0A034WC07	Uncharacterized protein	24901	219	23.74	4	0.05	down

A0A034VZ11	Uncharacterized protein (Fragment)	21843	231	27.71	5	0.05	down
A0A034V3D6	J domain-containing protein	19631	177	70.62	15	0.04	down
A0A034VM08	Uncharacterized protein	62881	556	59.71	40	0.04	down
A0A034WP07	Attacin-A	25021	239	72.8	14	0.03	down
A0A034WL03	Development-specific 25 kDa protein	28860	259	69.5	19	0.03	down
A0A034W0D7	Endocuticle structural glycoprotein ABD-4	14232	135	51.85	4	0.03	down
A0A034WXX0	Histone H1	24996	240	30.83	11	0.03	down
A0A034WSW4	Larval cuticle protein 8	11478	104	22.12	2	0.03	down
A0A034W585	Larval cuticle protein A3A	14397	138	75.36	17	0.03	down
A0A034WGB5	Uncharacterized protein	25308	221	55.2	12	0.03	down
A0A034WFT6	Uncharacterized protein	19920	195	59.49	14	0.03	down
A0A034VBG9	Uncharacterized protein	23773	229	65.07	16	0.03	down
A0A034V3B3	Arylphorin subunit A4 (Fragment)	140432	1224	45.92	55	0.02	down
A0A034WJQ4	Fat-body protein 1 (Fragment)	58417	511	48.53	31	0.02	down
A0A034V6N3	High mobility group protein Z	12805	112	33.04	5	0.02	down
A0A034VYM4	Larval serum protein 1 gamma chain (Fragment)	92074	773	31.31	27	0.02	down
A0A034W7C2	Uncharacterized protein	26739	235	43.83	10	0.02	down

Table S2. Proteins differentially expressed between 6- and 9-day-old ovaries of *Bactrocera dorsalis*.

Accession Number	Annotation	Protein Mass	Protein Length	Sequence Coverage	Peptides	Fold Change	Difference
A0A034WP50	Chorion protein S36 (Fragment)	24308	233	54.08	10	15.69	up
A0A034WSH3	Uncharacterized protein (Fragment)	18283	185	41.62	4	15.21	up
A0A034W023	Uncharacterized protein	43192	377	29.97	11	9.01	up
A0A034WJD1	3-ketoacyl-CoA thiolase, mitochondrial	41809	398	63.07	21	8.87	up
A0A034VSB5	Fasciclin-1	61398	542	35.42	17	8.35	up
A0A034VMV0	Putative 4-coumarate--CoA ligase 3	65064	587	29.64	13	7.50	up
A0A034VBU8	Uncharacterized protein (Fragment)	39869	352	32.95	8	6.86	up
A0A034V6M6	Antithrombin-III	47900	422	41.47	17	6.67	up
A0A034W2K6	Major royal jelly protein 1	47088	425	40.24	14	6.50	up
A0A034VUE5	Myotubularin-related protein 14	85100	765	30.59	18	6.21	up
A0A034VDF9	Uncharacterized protein (Fragment)	73996	639	28.64	13	6.14	up
A0A034WN56	Ubiquitin-like domain-containing CTD phosphatase 1	39155	334	31.74	9	6.07	up
A0A059XRL4	Ferritin	23169	204	56.86	10	5.97	up
G9F9Y5	Chitinase	54275	483	24.43	8	5.86	up
A0A034WB53	Cullin-5 (Fragment)	97906	850	16.24	12	5.75	up
A0A034VLB6	Ubiquitin-conjugating enzyme E2 C (Fragment)	12197	113	64.60	5	5.03	up
A0A034VCJ1	Malic enzyme	95358	869	53.74	39	4.85	up
A0A034V7D4	Microsomal triglyceride transfer protein large subunit (Fragment)	82310	737	34.60	24	4.82	up
A0A034WER3	Putative medium-chain specific acyl-CoA dehydrogenase, mitochondrial	45928	418	53.83	28	4.81	up
A0A034VAA7	Centrosomin (Fragment)	112702	992	10.58	8	4.80	up
A0A034W4S7	Succinyl-CoA:3-ketoacid coenzyme A transferase 1, mitochondrial (Fragment)	33458	309	32.04	7	4.79	up
A0A034W9D5	Glutaminyl-peptide cyclotransferase-like protein	37294	330	15.45	5	4.79	up
A0A034VAS8	Trifunctional enzyme subunit alpha, mitochondrial	83448	770	45.84	28	4.79	up
A0A034WRP5	Aminopeptidase N (Fragment)	69091	586	12.46	6	4.65	up
A0A034VKU6	Myotubularin-related protein 3	139934	1279	17.98	18	4.51	up
A0A034WT22	Uncharacterized protein	32198	290	21.72	6	4.50	up
A0A034WJ30	Uncharacterized protein	38270	326	23.01	6	4.41	up
A0A034W382	Peroxiredoxin 1	21784	194	68.56	15	4.26	up

A0A034VXX3	Uncharacterized protein	38416	353	40.51	10	4.25	up
A0A034WCE7	Insulin receptor	109284	963	30.43	21	4.24	up
A0A034VVU8	Muskelin	101135	890	38.20	32	4.04	up
A0A034W7B3	Uncharacterized protein	45156	395	40.25	15	3.98	up
A0A034VU99	Glutathione S-transferase S1	26938	240	65.83	17	3.92	up
A0A034WC12	Cytosolic endo-beta-N-acetylglucosaminidase	70022	604	30.30	15	3.88	up
A0A034W3G0	Chitinase-like protein Idgf4	48370	439	68.56	20	3.85	up
A0A034W030	MAP kinase-activating death domain protein (Fragment)	239912	2175	7.72	13	3.84	up
A0A034WF11	Trifunctional enzyme subunit beta, mitochondrial	52447	483	46.79	19	3.84	up
A0A034WHL8	Venom carboxylesterase-6	62660	549	25.14	12	3.84	up
A0A034WBX7	Ribonucleoside-diphosphate reductase	90971	804	46.27	34	3.83	up
A0A034VQ97	CD109 antigen	159290	1430	30.49	39	3.78	up
A0A034W812	Importin subunit alpha	57164	520	63.65	22	3.62	up
A0A034V8K5	Aminopeptidase N	119690	1035	19.81	16	3.55	up
A0A034V1H8	Filamin-A (Fragment)	141569	1301	54.04	58	3.54	up
A0A034VRJ4	Neutral alpha-glucosidase AB (Fragment)	51947	449	45.88	18	3.50	up
A0A034VI38	Lysosomal acid phosphatase	55250	477	33.75	12	3.49	up
A0A034VMU5	Flap endonuclease 1	42981	382	26.44	8	3.48	up
A0A034VRV3	Mitogen-activated protein kinase kinase kinase 15 (Fragment)	58729	508	10.63	5	3.48	up
A0A034WNV7	Peptide transporter family 1	78097	706	13.46	8	3.47	up
A0A034W7H6	Cyclin-dependent kinase 5-like protein	33341	293	14.68	4	3.46	up
A0A034WSJ2	Membrane-associated progesterone receptor component 2	25529	231	40.26	8	3.45	up
A0A034W1D2	UDP-glucuronosyltransferase 1-1 (Fragment)	74468	640	8.59	5	3.44	up
A0A034V1F4	Low-density lipoprotein receptor-related protein 2 (Fragment)	187971	1677	7.39	9	3.44	up
A0A034VLY5	V-type proton ATPase subunit S1	43114	388	30.15	9	3.42	up
A0A034WMH1	S-formylglutathione hydrolase	34158	305	45.25	10	3.41	up
A0A034V463	Low-density lipoprotein receptor-related protein 2 (Fragment)	341567	3066	10.89	28	3.39	up
A0A034V5Q6	Talin-1 (Fragment)	82628	783	31.67	17	3.34	up
A0A034V5J3	Glycerophosphocholine phosphodiesterase GPCPD1	74362	657	20.24	10	3.24	up
A0A034W3Z9	Vesicle-associated membrane protein/synaptobrevin-binding protein	28696	247	55.47	10	3.19	up
A0A034V5R2	Talin-1 (Fragment)	211049	1928	48.86	69	3.15	up

A0A034WA61	Maltase A3	71388	623	35.79	19	3.06	up
A0A034W647	Peptidyl-prolyl cis-trans isomerase D	51319	442	34.62	15	2.98	up
A0A034WDN8	Cytosolic non-specific dipeptidase	53660	479	55.95	21	2.91	up
A0A034W865	Gamma-tubulin complex component 3-like protein	107662	951	19.77	15	2.88	up
A0A034WSR6	Defective chorion-1 protein (Fragment)	79162	723	28.49	25	2.80	up
A0A034VEW6	Glyceraldehyde-3-phosphate dehydrogenase	35597	332	81.02	25	2.74	up
A0A034VIB7	Protein phosphatase 1B	41338	370	40.54	13	2.73	up
A0A034WNA4	GMP synthase (Glutamine-hydrolyzing)	76303	683	53.44	30	2.68	up
A0A034W397	Dual specificity mitogen-activated protein kinase kinase dSOR1	44030	397	44.58	18	2.67	up
A0A034V2C6	Serine/threonine-protein kinase Warts (Fragment)	68956	610	16.23	7	2.66	up
A0A034W787	Juvenile hormone epoxide hydrolase 1	52625	459	32.46	18	2.64	up
A0A034VS29	DNA mismatch repair protein MSH6	133796	1187	25.11	24	2.62	up
A0A034V3F4	Tripeptidyl-peptidase 2	149295	1353	47.97	54	2.61	up
A0A034VFZ2	Esterase FE4	66231	589	32.43	15	2.57	up
A0A034VZV8	Chorion peroxidase	94126	836	40.31	29	2.56	up
A0A034WVC4	UDP-N-acetylglucosamine--peptide N-acetylglucosaminyltransferase 110 kDa subunit	118456	1061	30.63	22	2.48	up
A0A034WAP1	Beta-ureidopropionase	43439	385	41.30	11	2.41	up
A0A034VXA2	Tubulin alpha-4 chain	51222	462	54.76	20	2.37	up
A0A034VIE8	Sulfhydryl oxidase	72338	640	31.09	12	2.35	up
A0A034VKF8	Cysteine and histidine-rich protein 1-like protein	49166	447	30.20	10	2.34	up
A0A034VFK7	Nuclear pore membrane glycoprotein 210	212907	1902	25.34	41	2.34	up
A0A034VMD8	Cytoskeleton-associated protein 5 (Fragment)	135423	1227	26.57	26	2.26	up
A0A034W4A7	Glutathione S-transferase 1-1	23716	207	49.76	11	2.26	up
A0A034WBQ5	Alpha-1,4 glucan phosphorylase	97349	845	61.07	50	2.20	up
A0A034VJU9	85/88 kDa calcium-independent phospholipase A2	96050	872	23.28	19	2.17	up
A0A034VKB5	Glucose-6-phosphate isomerase	62234	558	55.38	24	2.16	up
A0A034V6L1	Signal transducer and activator of transcription	84585	737	20.76	14	2.12	up
A0A034VU54	Neuroserpin (Fragment)	50223	451	37.47	14	2.12	up
A0A034W7N0	Protein NASP-like protein	46397	427	41.22	15	2.11	up
A0A034VV21	Nuclear pore complex protein Nup205 (Fragment)	124867	1088	22.79	20	2.11	up
A0A034VC08	Xaa-Pro dipeptidase	53654	480	42.29	18	2.09	up
A0A034WMG0	Ubiquitin carboxyl-terminal hydrolase	128892	1110	31.08	29	2.08	up
A0A034V4Z0	Uncharacterized protein	213808	1884	10.35	18	2.07	up

A0A034VK02	Dihydrolipoyl dehydrogenase	53044	504	58.33	26	1.98	up
A0A034UZ33	Importin subunit beta	98887	885	37.51	26	1.94	up
A0A034WQ10	Cullin-4A	97155	841	27.59	20	1.92	up
A0A034WUY1	Ubiquitin carboxyl-terminal hydrolase	25481	227	74.89	11	1.90	up
A0A034WAG6	Ran GTPase-activating protein	64995	587	34.07	19	1.87	up
A0A034WID4	Putative cation-transporting ATPase 13A1	136813	1216	18.67	19	1.79	up
A0A034WDU4	Endoplasmin	91351	797	56.21	46	1.74	up
A0A034VK16	Cytoskeleton-associated protein 5 (Fragment)	79847	734	32.70	20	1.69	up
A0A034WDM9	26S protease regulatory subunit 6A	47779	428	63.55	25	1.68	up
A0A034VG52	Adenosylhomocysteinase	56004	509	53.24	23	1.66	up
A0A034VPR3	Elongation factor Tu GTP-binding domain-containing protein 1	116814	1043	31.06	28	1.62	up
A0A034V085	Proteasome-associated protein ECM29-like protein	212089	1889	26.95	41	1.60	up
A0A034WBQ4	Putative ATP-dependent RNA helicase CG8611 (Fragment)	94276	841	17.24	12	0.62	down
A0A034VFT5	tRNA (Cytosine(34)-C(5))-methyltransferase	82282	728	33.52	16	0.57	down
A0A034WIR4	DnaJ-like protein subfamily C member 2	72239	618	28.48	14	0.56	down
A0A034WW15	40S ribosomal protein S3	27365	244	80.33	26	0.54	down
A0A034VGJ1	Clustered mitochondria protein homolog	153417	1362	30.54	35	0.53	down
A0A034WDZ9	Importin-5	123792	1106	53.35	47	0.53	down
A0A034WLA5	ATP-dependent RNA helicase vasa, isoform A	67940	621	57.49	35	0.51	down
A0A034V813	Acetyl-CoA carboxylase	270348	2391	34.84	58	0.51	down
A0A034W8Z3	Putative ATP-dependent RNA helicase DDX43	74677	662	47.28	27	0.51	down
A0A034WFM3	60S ribosomal protein L3	46663	413	50.36	26	0.51	down
A0A034VH70	La-related protein (Fragment)	132976	1206	24.71	20	0.50	down
A0A034VPD5	Villin-like protein quail	103012	895	18.21	13	0.49	down
A0A034VGU8	Eukaryotic translation initiation factor 2 subunit 3	51705	475	45.68	16	0.49	down
A0A034W6X9	GTP-binding protein 128up	41005	368	52.45	16	0.48	down
A0A034VFV1	Cytosolic 10-formyltetrahydrofolate dehydrogenase (Fragment)	75060	687	47.02	23	0.48	down
A0A034UZI7	Midasin (Fragment)	430710	3762	6.01	20	0.48	down
A0A034V6Q5	mRNA turnover protein 4-like protein	29891	257	28.79	8	0.47	down
W5ZSP5	Dicer2	192043	1672	24.88	36	0.47	down
A0A034VFE7	Far upstream element-binding protein 2	82953	794	23.68	16	0.47	down
A0A034WCL6	Peptidyl-prolyl cis-trans isomerase FKBP6 (Fragment)	54511	478	43.93	16	0.46	down

A0A034VZY9	Heat shock 70 kDa protein 4	89947	810	60.25	44	0.45	down
A0A034VMS2	Peptide methionine sulfoxide reductase	27545	243	29.63	6	0.45	down
A0A034W0U6	Pre-rRNA-processing protein TSR1-like protein	86592	756	18.92	13	0.45	down
A0A034VLD7	Peptidyl-prolyl cis-trans isomerase	111376	996	27.61	25	0.44	down
A0A034W524	Annexin	36449	322	44.41	13	0.44	down
A0A034VJX4	Very long-chain-fatty-acid--CoA ligase bubblegum	74013	666	30.33	15	0.43	down
A0A034VBD0	Putative ATP-dependent RNA helicase DDX5	79548	732	30.60	18	0.43	down
A0A034VGS8	Tubulin beta-3 chain	50889	454	60.79	27	0.42	down
A0A034VN85	Eukaryotic translation initiation factor 3 subunit E	50964	434	38.71	15	0.42	down
A0A034VER3	Eukaryotic translation initiation factor 3 subunit C	108335	923	37.92	32	0.42	down
A0A034WST6	Selenide, water dikinase	43668	400	52.25	17	0.42	down
A0A034WKB0	40S ribosomal protein S5a	25415	227	32.60	7	0.42	down
A0A034WA29	Aspartate--tRNA ligase, mitochondrial	120485	1077	30.64	29	0.41	down
A0A034WTX8	Pescadillo-like protein (Fragment)	72609	613	8.97	5	0.41	down
A0A034VYR3	Nucleolar protein 58	81673	728	43.82	30	0.41	down
A0A034VWU6	La protein-like protein	48148	413	40.19	15	0.41	down
A0A034WMJ8	40S ribosomal protein S4 (Fragment)	31529	280	59.29	23	0.41	down
A0A034WLF6	FK506-binding protein 59	48986	437	59.27	29	0.41	down
A0A034W3W4	Calponin	19151	169	71.01	13	0.40	down
A0A034VAP8	Eukaryotic initiation factor 4A-III	46062	402	31.59	14	0.39	down
A0A034VQT4	DNA topoisomerase 1	115728	1000	7.10	7	0.39	down
A0A034VV96	Symplekin	133220	1175	8.17	7	0.39	down
A0A034V7Y9	Eukaryotic peptide chain release factor GTP-binding subunit ERF3A	66511	616	42.37	25	0.39	down
A0A034W3J9	Calnexin	69222	616	23.21	12	0.38	down
A0A034W1D4	Hepatoma-derived growth factor	61550	545	30.09	15	0.38	down
A0A034W954	Protein RRP5-like protein	163045	1433	17.52	19	0.37	down
A0A034V9A3	CUGBP Elav-like family member 2	62542	587	25.55	13	0.37	down
A0A034VKH4	Innexin	42257	365	23.29	9	0.36	down
A0A034VJF0	Ribosome biogenesis protein BOP1 homolog	97678	845	19.05	12	0.35	down
A0A034VH20	Piwi-like protein	102440	900	49.56	37	0.35	down
A0A034VB23	RRP12-like protein	154813	1385	12.92	16	0.35	down
A0A034VQY4	Protein bric-a-brac 2	101626	929	17.01	11	0.35	down
A0A034VX01	DNA-binding protein modulo (Fragment)	65113	578	37.89	22	0.35	down
A0A034V5U3	Staphylococcal nuclease domain-containing protein 1	103711	928	64.12	47	0.35	down

A0A034WKG9	Ecdysteroid UDP-glucosyltransferase	62370	549	24.77	10	0.35	down
A0A034VQG9	Protein penguin	79228	703	17.64	11	0.35	down
A0A034V4U2	Putative ATP-dependent RNA helicase spindle-E (Fragment)	82430	722	32.55	19	0.34	down
A0A034WPR9	Asparagine synthetase	63212	557	11.85	6	0.33	down
A0A034VGV1	Tudor domain-containing protein 1 (Fragment)	136071	1183	24.09	23	0.32	down
A0A034WRG8	Putative ribosomal RNA methyltransferase NOP2	93568	812	16.87	11	0.32	down
A0A034W2L3	Citrate synthase (Fragment)	28581	257	63.42	15	0.31	down
A0A034V7N4	Myosin heavy chain 95F	141826	1240	28.63	32	0.30	down
A0A034WGD2	Ribosomal L1 domain-containing protein CG13096 (Fragment)	68771	608	32.07	18	0.29	down
A0A034VD80	RNA-binding protein squid (Fragment)	30830	281	59.07	14	0.28	down
A0A034WSY7	Nucleolar GTP-binding protein 1	75319	651	23.04	13	0.28	down
A0A034WPS6	Inositol-3-phosphate synthase	62553	568	42.96	18	0.27	down
A0A034VSG4	Maternal protein exuperantia	55182	495	51.11	26	0.27	down
A0A034VY07	Nucleolar protein 56	57438	512	34.18	13	0.26	down
A0A034VTB7	Uncharacterized protein	47334	421	42.76	12	0.26	down
A0A034WTE5	Trans-1,2-dihydrobenzene-1,2-diol dehydrogenase	37022	332	35.24	13	0.25	down
A0A034VYF9	Paramyosin, long form	102321	879	53.70	41	0.25	down
A0A034VCY4	Tudor domain-containing protein 7 (Fragment)	108951	956	25.63	18	0.25	down
A0A034VR52	Procollagen-lysine,2-oxoglutarate 5-dioxygenase 1	85847	743	24.23	18	0.24	down
A0A034WS42	Histone H4	11382	103	65.05	15	0.24	down
A0A034WJE4	Transcription elongation factor SPT5	120771	1090	16.24	13	0.24	down
A0A034WJ92	Protein piwi (Fragment)	78854	688	51.45	37	0.24	down
A0A034WUE5	Ribosome production factor 2-like protein	36400	314	30.25	9	0.23	down
A0A034WNU4	H/ACA ribonucleoprotein complex subunit 2-like protein	17742	159	43.40	5	0.23	down
A0A034WST2	ATP-dependent RNA helicase WM6	48520	423	55.08	22	0.23	down
A0A034VR92	Tropomyosin-2	32743	284	57.04	17	0.22	down
A0A034W3Y6	RNA-binding protein 1	16004	142	30.99	6	0.22	down
A0A034VA48	Prolyl 4-hydroxylase subunit alpha-2	64303	561	23.53	9	0.21	down
A0A034V087	Guanine nucleotide-binding-like protein 3-like protein (Fragment)	69470	610	29.02	16	0.20	down
A0A034WG23	ATP-dependent RNA helicase p62	81987	757	51.12	31	0.20	down
A0A034WL04	RNA-binding protein lark	38612	338	47.34	15	0.20	down
A0A034WNF6	Importin subunit alpha	57681	519	29.09	12	0.19	down

A0A034VUY6	Methenyltetrahydrofolate synthase domain-containing protein	59463	538	56.51	32	0.16	down
A0A034V1Z3	Atlastin (Fragment)	91611	791	48.93	37	0.16	down
A0A034WHQ6	Nucleolar GTP-binding protein 2	77079	674	15.73	9	0.16	down
A0A034VSE8	Serrate RNA effector molecule-like protein	113786	997	19.16	17	0.11	down
A0A034WUS8	Protein maelstrom	58096	514	17.90	7	0.10	down
A0A034UX56	Trehalose 6-phosphate phosphatase	31189	274	81.75	25	0.08	down

Table S3. Proteins differentially expressed between 1- and 9-day-old ovaries of *Bactrocera dorsalis*.

Accession Number	Annotation	Protein Mass	Protein Length	Sequence Coverage	Peptides	Fold Change	Difference
A0A034WA19	Chorion protein S38	27957	283	32.51	8	48.89	up
A0A034WSR6	Defective chorion-1 protein (Fragment)	79162	723	28.49	25	45.64	up
A0A034WP50	Chorion protein S36 (Fragment)	24308	233	54.08	10	36.76	up
A0A034WHP3	Vitelline membrane protein Vm26Aa	11467	114	82.46	16	35.57	up
A0A034VWY8	Fatty-acid amide hydrolase 2	58853	530	40.94	20	34.15	up
A0A034WSH3	Uncharacterized protein (Fragment)	18283	185	41.62	4	33.82	up
A0A034WY57	Putative ATP-dependent RNA helicase me31b	51888	457	77.46	46	32.45	up
A0A034W023	Uncharacterized protein	43192	377	29.97	11	31.16	up
A0A034V1T8	Putative vitellogenin receptor	214000	1923	36.45	58	30.29	up
A0A034VQJ3	Vitellogenin-1	47853	436	83.26	82	29.72	up
A0A034VUE5	Myotubularin-related protein 14	85100	765	30.59	18	29.22	up
A0A034VE18	Heat shock protein 23	18967	170	78.82	15	29.02	up
A0A034W787	Juvenile hormone epoxide hydrolase 1	52625	459	32.46	18	26.88	up
A0A034W812	Importin subunit alpha	57164	520	63.65	22	26.60	up
A0A034VXX3	Uncharacterized protein	38416	353	40.51	10	26.18	up
A0A034W7B3	Uncharacterized protein	45156	395	40.25	15	25.66	up
A0A034WRE4	60S ribosomal protein L30	12248	111	60.36	9	23.90	up
A0A034V5D1	Chorion protein S16	14769	144	24.31	5	23.56	up
A0A034V196	Protein disulfide-isomerase	15091	135	51.85	6	23.15	up
Q8T7E3	Vitellogenin 1	47781	436	83.26	77	21.25	up
A0A034VDF9	Uncharacterized protein (Fragment)	73996	639	28.64	13	21.09	up
A0A034VZV8	Chorion peroxidase	94126	836	40.31	29	20.11	up
A0A034W6U8	Glucose dehydrogenase (Acceptor)	71439	633	21.48	10	19.74	up
A0A034WNR3	46 kDa FK506-binding nuclear protein (Fragment)	20602	180	28.89	4	19.57	up
A0A034WWC1	General odorant-binding protein 99b	16792	143	49.65	10	19.36	up

A0A034V1J5	Uncharacterized protein (Fragment)	54156	481	19.33	8	18.28	up
A0A034VVU8	Muskelin	101135	890	38.20	32	17.65	up
A0A034W030	MAP kinase-activating death domain protein (Fragment)	239912	2175	7.72	13	17.31	up
A0A034UZ33	Importin subunit beta	98887	885	37.51	26	16.20	up
A7YVH6	Cytochrome b	43024	378	2.38	1	15.96	up
A0A034VP94	40S ribosomal protein S12	15218	139	84.17	16	15.62	up
A0A034V4Z0	Uncharacterized protein	213808	1884	10.35	18	15.53	up
A0A034W432	Cytochrome P450 306a1	63391	545	40.92	19	15.43	up
A0A034WEA0	FAM203 family protein CG6073	43202	376	26.33	10	15.16	up
A0A034VFV2	6-phosphogluconate dehydrogenase, decarboxylating	52780	481	63.20	27	15.02	up
A0A034W6L5	Importin-7	119886	1048	37.60	31	14.03	up
A0A034VJ67	Protein cup (Fragment)	127850	1173	22.25	24	13.94	up
A0A034V6V9	Importin-4	120939	1087	42.23	40	13.56	up
A0A034WBZ8	Putative serine protease K12H4.7	59035	517	39.65	19	13.40	up
A0A034W0T2	Inosine-5'-monophosphate dehydrogenase	57498	534	35.96	12	13.26	up
A0A034WID2	RIP-like protein	25269	220	13.64	3	13.24	up
A0A034W6K6	Tetraspanin	26383	236	22.46	5	13.22	up
A0A034WGD6	Protein zwilch	73614	646	18.27	10	13.16	up
A0A034W1V3	Diphthine synthase	31790	280	47.14	9	12.99	up
A0A034VHX8	DEP domain-containing protein 5 (Fragment)	54054	465	6.88	3	12.96	up
A0A034W1D2	UDP-glucuronosyltransferase 1-1 (Fragment)	74468	640	8.59	5	12.79	up
A0A034UZF8	Uncharacterized protein (Fragment)	150148	1341	6.26	8	12.74	up
A0A034WT22	Uncharacterized protein	32198	290	21.72	6	12.35	up
A0A034VUT0	Protein singed (Fragment)	56975	509	31.43	13	12.32	up
A0A034WCS4	Dehydrogenase/reductase SDR family member 11	27759	251	57.77	8	12.22	up
I1SWI8	Heat shock protein 60	61054	573	77.66	47	12.15	up
A0A034VC07	Thioredoxin reductase 1, mitochondrial	53451	493	71.40	28	12.01	up
A0A034VLP4	Transforming growth factor-beta-induced protein ig-h3	89994	810	44.69	28	12.00	up
A0A034WPW1	Importin-13	111854	975	5.74	5	11.90	up
A0A034V1Z6	Valine--tRNA ligase	120041	1054	39.75	36	11.89	up
A0A034WKN9	C2 domain-containing protein 3 (Fragment)	75399	682	33.43	14	11.82	up
A0A034W4L6	Lipid storage droplets surface-binding protein 2	37989	353	61.47	20	11.82	up
A0A034VXQ0	Phosphate carrier protein, mitochondrial	38352	353	43.91	15	11.37	up
A0A034WDZ9	Importin-5	123792	1106	53.35	47	11.19	up

A0A034W397	Dual specificity mitogen-activated protein kinase kinase dSOR1	44030	397	44.58	18	11.14	up
A0A034V3F4	Tripeptidyl-peptidase 2	149295	1353	47.97	54	11.08	up
A0A034WNG6	Alanine--tRNA ligase, cytoplasmic	108265	967	50.26	38	11.06	up
A0A034V085	Proteasome-associated protein ECM29-like protein	212089	1889	26.95	41	11.06	up
A0A034VMA5	ATP-binding cassette sub-family F member 2	69942	610	27.70	17	10.91	up
A0A034VPR3	Elongation factor Tu GTP-binding domain-containing protein 1	116814	1043	31.06	28	10.71	up
A0A034VXE8	Uncharacterized protein	27095	242	35.12	6	10.69	up
A0A034WWE5	Uncharacterized protein (Fragment)	11638	102	35.29	3	10.67	up
A0A034VXK9	Aspartate aminotransferase (Fragment)	42034	376	76.86	26	10.59	up
A0A034WID4	Putative cation-transporting ATPase 13A1	136813	1216	18.67	19	10.57	up
A0A034VC08	Xaa-Pro dipeptidase	53654	480	42.29	18	10.51	up
A0A034VML9	Importin-9	114510	1018	33.10	25	10.37	up
A0A034WW15	40S ribosomal protein S3	27365	244	80.33	26	10.31	up
A0A034WCE7	Insulin receptor	109284	963	30.43	21	10.20	up
A0A034VUA6	26S proteasome non-ATPase regulatory subunit 1	112769	1017	52.51	45	10.15	up
A0A034W2M7	Elongation factor 1-gamma (Fragment)	49900	438	58.68	31	10.04	up
A0A034WJ30	Uncharacterized protein	38270	326	23.01	6	10.00	up
A0A034W486	Protein transport protein Sec61 subunit alpha isoform 2 (Fragment)	37285	340	20.29	7	9.94	up
A0A034W9W8	Uncharacterized protein	28452	247	22.67	4	9.94	up
A0A034VI39	Bleomycin hydrolase	52713	461	57.70	26	9.85	up
A0A034WEU3	Enoyl-CoA delta isomerase 2, mitochondrial	28863	260	48.46	10	9.78	up
A0A034W9D5	Glutaminyl-peptide cyclotransferase-like protein	37294	330	15.45	5	9.60	up
A0A034VED9	UPF0586 protein CG11596	50004	435	13.10	6	9.59	up
A0A034WD99	Pyruvate kinase	58030	535	81.50	42	9.54	up
A0A034VA47	Uncharacterized protein (Fragment)	96787	853	21.81	18	9.52	up
A0A034WKN7	Methyltransferase-like protein 13	76261	675	33.63	19	9.51	up
A0A034WF02	Uncharacterized protein	42149	373	16.35	5	9.48	up
A0A034WB81	T-complex protein 1 subunit gamma	59276	543	71.27	41	9.44	up
A0A034WBX7	Ribonucleoside-diphosphate reductase	90971	804	46.27	34	9.34	up
A0A034W0V2	Cystathionine beta-synthase	56287	515	13.40	6	9.20	up
A0A034V8K3	RING finger protein 17 (Fragment)	76371	675	34.81	17	9.17	up
A0A034VG52	Adenosylhomocysteinase	56004	509	53.24	23	9.11	up
A0A034V7V4	Uncharacterized protein (Fragment)	123947	1092	18.50	16	9.10	up

A0A034VR21	Protein bicaudal C	98705	899	26.25	15	9.06	up
A0A034VZW3	Transitional endoplasmic reticulum ATPase TER94	88721	799	65.08	52	8.87	up
A0A034V392	Receptor expression-enhancing protein	34730	304	12.50	3	8.84	up
A0A034WGZ4	Elongation factor Ts, mitochondrial	33818	305	40.66	9	8.81	up
A0A034WMH5	26S proteasome non-ATPase regulatory subunit 13	43727	382	47.64	20	8.79	up
A0A034WM52	Cullin-associated NEDD8-dissociated protein 1	139107	1236	39.56	41	8.77	up
A0A034WM27	Tubulin--tyrosine ligase-like protein 12	74892	641	29.49	17	8.49	up
A0A034V3E5	Nuclear pore complex protein Nup107 (Fragment)	103735	899	34.59	25	8.46	up
A0A034WE93	Glycine--tRNA ligase	76602	679	50.07	31	8.36	up
A0A034VGT4	Tetratricopeptide repeat protein 37	140255	1234	7.05	6	8.26	up
A0A034UZ41	Sepiapterin reductase	29503	263	50.57	10	8.20	up
A0A034W9N5	26S proteasome non-ATPase regulatory subunit 8	30629	265	70.57	19	8.19	up
A0A034V3I0	CAD protein (Fragment)	136404	1234	20.83	17	8.12	up
A0A034WL96	60S ribosomal protein L13	25137	216	39.81	13	8.08	up
A0A034VA13	Eukaryotic initiation factor 4A	45755	403	67.00	28	8.02	up
A0A034V3P5	Uncharacterized protein (Fragment)	114127	1011	15.03	11	7.99	up
A0A034V8K5	Aminopeptidase N	119690	1035	19.81	16	7.97	up
A0A034V3F2	26S protease regulatory subunit 7	48544	433	62.82	30	7.91	up
A0A034W163	Xanthine dehydrogenase (Fragment)	143901	1298	19.95	21	7.88	up
A0A034VV21	Nuclear pore complex protein Nup205 (Fragment)	124867	1088	22.79	20	7.84	up
A0A034W3L0	Ankyrin repeat and KH domain-containing protein mask (Fragment)	54726	507	29.98	11	7.83	up
A0A034WWD8	Exportin-1	123439	1070	40.56	37	7.83	up
A0A034W1C0	Eukaryotic translation initiation factor 4 gamma 2	106157	942	27.81	19	7.82	up
A0A034VKU6	Myotubularin-related protein 3	139934	1279	17.98	18	7.79	up
A0A034VIB7	Protein phosphatase 1B	41338	370	40.54	13	7.73	up
A0A034WIQ8	Phosphoserine aminotransferase	40024	366	59.29	18	7.70	up
A0A034WC12	Cytosolic endo-beta-N-acetylglucosaminidase	70022	604	30.30	15	7.67	up
A0A034VI37	Glutamyl aminopeptidase	118712	1045	34.64	31	7.53	up
A0A034VV64	Eukaryotic translation initiation factor 3 subunit A	132786	1126	34.46	37	7.47	up
A0A034WM97	Glutathione S-transferase 1	24717	222	59.46	14	7.46	up

A0A034VYI4	Tyrosine--tRNA ligase (Fragment)	58023	523	65.01	29	7.46	up
A0A034V173	Putative N-acetyltransferase san	21206	186	50.54	10	7.46	up
A0A034WUY1	Ubiquitin carboxyl-terminal hydrolase	25481	227	74.89	11	7.42	up
A0A034V1A5	Putative 6-phosphogluconolactonase	26291	238	59.66	12	7.27	up
A0A034WEC5	Uncharacterized protein	14367	129	30.23	3	7.25	up
A0A034VH61	Putative aminopeptidase W07G4.4	54904	505	59.80	27	7.23	up
A0A034VUQ7	Glucose-6-phosphate 1-dehydrogenase	60835	527	52.56	22	7.12	up
A0A034VU00	Sorting nexin-16	49595	447	11.63	4	7.01	up
A0A034V1D4	CAD protein (Fragment)	111533	999	28.43	25	6.88	up
A0A034VK02	Dihydrolipoyl dehydrogenase	53044	504	58.33	26	6.88	up
A0A034WUZ1	Protein canopy-like protein 2	21486	191	39.27	6	6.77	up
A0A034VAA7	Centrosomin (Fragment)	112702	992	10.58	8	6.77	up
A0A034VFZ9	Kinetochore-associated protein 1 (Fragment)	89215	770	26.36	17	6.74	up
A0A034VMD8	Cytoskeleton-associated protein 5 (Fragment)	135423	1227	26.57	26	6.72	up
A0A034VEP1	Exportin-5	43240	388	18.56	5	6.69	up
A0A034VQT5	Serine/threonine-protein kinase polo	67246	576	29.69	13	6.60	up
A0A034WJW9	26S proteasome non-ATPase regulatory subunit 5	56937	499	19.24	9	6.58	up
A0A034V1T1	Protein purity of essence (Fragment)	110879	976	15.16	12	6.58	up
A0A034W647	Peptidyl-prolyl cis-trans isomerase D	51319	442	34.62	15	6.55	up
A0A034WU84	Proteasome subunit alpha type	28026	253	60.08	14	6.49	up
A0A034WDU4	Endoplasmin	91351	797	56.21	46	6.47	up
A0A034VLF2	Coatomer subunit beta	106993	953	33.47	26	6.38	up
A0A034W3A2	Serine--tRNA ligase, cytoplasmic	56415	497	49.09	17	6.37	up
A0A034V8L4	Aldehyde dehydrogenase, mitochondrial (Fragment)	44957	413	72.15	21	6.30	up
A0A034WPA4	28S ribosomal protein S35, mitochondrial	38291	332	31.02	8	6.28	up
A0A034VJH4	N-alpha-acetyltransferase 16, NatA auxiliary subunit	101634	878	33.94	28	6.26	up
G9F9Y5	Chitinase	54275	483	24.43	8	6.26	up
A0A034VFI5	Centromere/kinetochore protein zw10	81461	708	17.94	11	6.22	up
A0A034VZH7	26S proteasome non-ATPase regulatory subunit 2	101079	911	48.74	35	6.17	up
A0A034WPS5	COP9 signalosome complex subunit 1b	56677	511	25.24	10	6.12	up
A0A034WF61	Serine/threonine-protein phosphatase	34736	303	36.96	9	6.10	up
A0A034WFC0	ATP synthase subunit gamma	33092	297	38.72	12	6.08	up
A0A034VEZ1	Glutamate synthase (NADH), amyloplastic	230930	2093	43.91	77	6.03	up
A0A034W6I8	Exportin-2	109799	972	34.88	33	5.94	up
A0A034VUM9	Protein amalgam	36389	329	54.71	15	5.93	up

A0A034VGJ0	Putative multidrug resistance-associated protein lethal(2)03659	57681	513	21.64	9	5.91	up
A0A034WRK8	Farnesyl pyrophosphate synthase	46845	408	17.16	6	5.90	up
A0A034W4A7	Glutathione S-transferase 1-1	23716	207	49.76	11	5.86	up
A0A034WJJ8	Defective in cullin neddylation protein	28327	243	24.69	5	5.86	up
A0A034VM32	CCR4-NOT transcription complex subunit 1 (Fragment)	123312	1091	20.99	20	5.82	up
A0A034VU18	Protein ref(2)P	76359	702	27.78	12	5.78	up
A0A034VWI5	Leucine-rich PPR motif-containing protein, mitochondrial (Fragment)	159347	1418	43.86	50	5.75	up
A0A034VGJ1	Clustered mitochondria protein homolog	153417	1362	30.54	35	5.70	up
A0A034V2H3	Protein purity of essence (Fragment)	104460	945	15.24	10	5.65	up
A0A034WDM9	26S protease regulatory subunit 6A	47779	428	63.55	25	5.63	up
A0A034WFM3	60S ribosomal protein L3	46663	413	50.36	26	5.58	up
A0A034WIJ0	COP9 signalosome complex subunit 4	46518	407	61.67	21	5.58	up
A0A034VEP3	4-aminobutyrate aminotransferase, mitochondrial	54817	491	48.88	18	5.56	up
A0A034VBA8	Isoleucine--tRNA ligase, cytoplasmic (Fragment)	53686	468	38.46	16	5.54	up
A0A034V4X1	Proton-coupled amino acid transporter 4	48307	443	11.74	4	5.54	up
A0A034WU78	Fumarate hydratase, mitochondrial	54067	500	48.00	18	5.49	up
A0A034V813	Acetyl-CoA carboxylase	270348	2391	34.84	58	5.48	up
A0A034VUM4	Protein extra bases (Fragment)	48866	420	46.19	23	5.47	up
A0A034WR67	COP9 signalosome complex subunit 5	37494	333	41.14	12	5.43	up
A0A034V540	Uncharacterized protein	28794	253	43.08	9	5.42	up
A0A034VKN3	Leucine--tRNA ligase, cytoplasmic	135606	1182	45.18	44	5.40	up
A0A034VRR7	E3 ubiquitin-protein ligase listerin	193519	1664	9.74	14	5.39	up
A0A034VDY8	Bifunctional glutamate/proline--tRNA ligase	185312	1636	48.66	65	5.35	up
A0A034VAK1	Heat shock protein 75 kDa, mitochondrial	80438	710	42.25	25	5.33	up
A0A034VER4	Ubiquitin-conjugating enzyme E2 O	172146	1542	11.22	13	5.32	up
A0A034VGD9	DNA primase (Fragment)	45606	397	11.84	5	5.30	up
A0A034W4L2	Enhancer of mRNA-decapping protein 3	64715	585	19.66	8	5.26	up
A0A034V786	AP-3 complex subunit delta	116643	1033	8.03	8	5.26	up
A0A034W4W3	Transportin-1	100645	886	27.20	18	5.22	up
A0A034WJ52	Eukaryotic translation initiation factor 4 gamma 1	195326	1766	36.98	46	5.18	up
A0A034WFU9	Uncharacterized protein	37742	341	5.86	2	5.17	up
A0A034VKX9	Alkyldihydroxyacetonephosphate synthase (Fragment)	76813	689	22.35	11	5.15	up

A0A034UZB5	Protein purity of essence (Fragment)	237724	2178	13.77	25	5.13	up
A0A034WB53	Cullin-5 (Fragment)	97906	850	16.24	12	5.13	up
A0A034V9L3	Poly(A) RNA polymerase gld-2-like protein A	62204	550	9.09	5	5.09	up
A0A034WIL4	NADH dehydrogenase (Ubiquinone) iron-sulfur protein 2, mitochondrial	53289	473	43.97	18	5.08	up
A0A034VPQ5	Actin-related protein 2/3 complex subunit 4	19578	168	22.62	4	5.02	up
A0A034W6A3	Microtubule-associated protein RP/EB family member 1	28965	253	22.13	6	5.02	up
A0A034V4D6	DnaJ-like protein subfamily C member 13 (Fragment)	220756	1936	21.02	31	5.00	up
A0A034W450	Uncharacterized protein	29321	264	39.77	11	5.00	up
A0A034WDC3	DNA replication inhibitor plutonium (Fragment)	20902	186	24.19	3	4.99	up
A0A034W865	Gamma-tubulin complex component 3-like protein	107662	951	19.77	15	4.98	up
A0A034VNQ2	Nuclear pore complex protein Nup85	77843	677	36.63	19	4.96	up
A0A034WNA4	GMP synthase (Glutamine-hydrolyzing)	76303	683	53.44	30	4.92	up
A0A034WFJ7	Putative elongator complex protein 1 (Fragment)	118063	1025	26.63	23	4.91	up
A0A034VL30	Nuclear pore complex protein Nup160-like protein (Fragment)	119129	1044	18.68	13	4.78	up
A0A034WRP8	Alpha-tocopherol transfer-like protein	36234	311	13.50	4	4.74	up
A0A034W6Z5	26S proteasome non-ATPase regulatory subunit 11	47258	421	57.96	27	4.73	up
A0A034WDL1	NADH-ubiquinone oxidoreductase 75 kDa subunit, mitochondrial	80086	736	46.47	29	4.72	up
A0A034W2K6	Major royal jelly protein 1	47088	425	40.24	14	4.68	up
A0A034WHL8	Venom carboxylesterase-6	62660	549	25.14	12	4.68	up
A0A034UZI2	Aladin	52368	467	37.47	14	4.62	up
A0A034WUW3	UPF0160 protein MYG1, mitochondrial (Fragment)	44465	385	37.14	12	4.61	up
A0A034VCH0	1-acylglycerophosphocholine O-acyltransferase 1	61466	538	26.21	10	4.58	up
A0A034VDI3	Vacuolar protein sorting-associated protein 35	91897	808	24.50	17	4.57	up
A0A034W3T2	Serine/threonine-protein phosphatase 5	61618	544	40.26	19	4.56	up
A0A034V555	Ubiquitin carboxyl-terminal hydrolase (Fragment)	138806	1221	14.58	15	4.48	up
A0A034VW60	Apoptosis-inducing factor 3	61371	557	26.93	13	4.46	up
A0A034VTP3	TBC1 domain family member 5	80221	695	11.65	7	4.45	up

A0A034VRQ0	Translational activator GCN1 (Fragment)	161957	1471	34.81	39	4.44	up
A0A034WCD3	NEDD8-activating enzyme E1 regulatory subunit	58530	522	29.12	13	4.43	up
A0A034VX54	Aspartate aminotransferase, cytoplasmic	45679	407	60.69	20	4.41	up
A0A034W9F2	Histone-lysine N-methyltransferase setd3	61477	534	36.14	17	4.37	up
A0A034V9G7	Eukaryotic translation initiation factor 5B	134978	1215	24.86	20	4.36	up
A0A034VMW4	Uridine 5'-monophosphate synthase	53209	487	49.08	20	4.35	up
A0A034WMG0	Ubiquitin carboxyl-terminal hydrolase	128892	1110	31.08	29	4.28	up
A0A034WJS4	Protein l(2)37Cc	30367	276	85.14	21	4.24	up
A0A034W9B6	T-complex protein 1 subunit zeta	58468	531	64.22	28	4.09	up
A0A034VAG4	Tudor domain-containing protein 12	96482	848	10.73	8	4.01	up
A0A034W1H3	Nuclear pore complex protein Nup93	92985	809	38.32	27	4.00	up
A0A034VN48	Putative fatty acyl-CoA reductase CG5065	64021	566	12.19	7	3.98	up
A0A034V5U4	6-phosphofructokinase	86075	781	34.83	19	3.96	up
A0A034VBM4	Trifunctional purine biosynthetic protein adenosine-3	148058	1368	35.75	39	3.96	up
A0A034VPD5	Villin-like protein quail	103012	895	18.21	13	3.93	up
A0A034WNJ6	Heat shock 70 kDa protein cognate 5	73628	679	55.23	34	3.91	up
A0A034VRJ4	Neutral alpha-glucosidase AB (Fragment)	51947	449	45.88	18	3.91	up
A0A034VK16	Cytoskeleton-associated protein 5 (Fragment)	79847	734	32.70	20	3.90	up
A0A034VK83	Isochorismatase domain-containing protein 1	23205	207	43.48	9	3.88	up
A0A034VV03	Protein phosphatase methylesterase 1	46122	416	16.83	6	3.85	up
A0A034WCY3	Dedicator of cytokinesis protein 3 (Fragment)	204946	1790	5.81	9	3.85	up
A0A034W7H6	Cyclin-dependent kinase 5-like protein	33341	293	14.68	4	3.82	up
A0A034V6X7	Multidrug resistance-associated protein 1	174106	1550	27.42	33	3.76	up
A0A034W5B8	Uncharacterized protein (Fragment)	42772	431	20.65	5	3.74	up
A0A034VS83	Hypoxia up-regulated protein 1	107546	957	42.11	30	3.67	up
A0A034WTT2	Proteasome subunit beta type-1	25619	231	67.10	16	3.65	up
A0A034V6T2	Insulin-degrading enzyme	120515	1048	24.43	24	3.65	up
A0A034V2C6	Serine/threonine-protein kinase Warts (Fragment)	68956	610	16.23	7	3.62	up
A0A034VQ24	60S ribosomal export protein NMD3	59124	517	30.95	12	3.61	up
A0A034VF36	Ubiquitin-conjugating enzyme E2 Q2	43953	389	9.77	3	3.57	up
A0A034VG90	Nuclear pore complex protein Nup133	135450	1193	20.12	19	3.54	up
A0A034WVA3	Elongation factor Tu	53920	490	49.39	19	3.53	up
A0A034W9M9	Cytosolic 10-formyltetrahydrofolate dehydrogenase (Fragment)	25559	231	47.19	9	3.49	up
A0A034VF44	WD repeat-containing protein mio (Fragment)	69582	613	15.33	8	3.47	up
A0A034WIU7	Tetratricopeptide repeat protein 27	91135	792	16.67	11	3.46	up

A0A034VLV8	Glutamyl-tRNA(Gln) amidotransferase subunit A, mitochondrial	45422	414	17.87	6	3.46	up
A0A034VKB0	Transforming acidic coiled-coil-containing protein 1	158551	1441	13.60	15	3.43	up
A0A034VH80	Calcium-binding mitochondrial carrier protein Aralar1	74442	671	34.13	22	3.41	up
A0A034W952	Dymeclin	79918	702	14.81	8	3.41	up
A0A034W7N0	Protein NASP-like protein	46397	427	41.22	15	3.40	up
A0A034WQ10	Cullin-4A	97155	841	27.59	20	3.35	up
A0A034VFE4	DDB1-and CUL4-associated factor-like 1 (Fragment)	106321	962	12.89	9	3.35	up
A0A034WRS4	60S acidic ribosomal protein P2	11743	113	74.34	9	3.31	up
A0A034VAS4	Acetyltransferase component of pyruvate dehydrogenase complex	53683	503	39.36	14	3.31	up
A0A034VN77	Rab proteins geranylgeranyltransferase component A	63386	571	24.87	10	3.30	up
A0A034VLI5	Protein-tyrosine-phosphatase	67935	604	22.85	10	3.28	up
A0A034W439	Ubiquitin carboxyl-terminal hydrolase	90946	809	43.39	26	3.26	up
A0A034V250	Glycogen debranching enzyme	210785	1878	18.69	32	3.22	up
A0A034W273	Aspartate--tRNA ligase, cytoplasmic	60020	539	49.91	25	3.16	up
A0A034WJX0	Protein croquemort	55866	488	21.52	8	3.14	up
A0A034VQ97	CD109 antigen	159290	1430	30.49	39	3.11	up
A0A034WAN7	Signal recognition particle 54 kDa protein	55927	508	36.22	12	3.09	up
A0A034VI89	Conserved oligomeric Golgi complex subunit 6	71503	628	20.06	9	3.07	up
A0A034WJH2	Protein dpy-30-like protein	10966	99	47.47	4	3.00	up
A0A034WBK6	Nuclear pore complex protein Nup155	158067	1404	20.66	21	2.97	up
A0A034VXN0	Short/branched chain specific acyl-CoA dehydrogenase, mitochondrial	45479	413	41.16	12	2.94	up
A0A034VKF8	Cysteine and histidine-rich protein 1-like protein	49166	447	30.20	10	2.88	up
A0A034VDF3	Squamous cell carcinoma antigen recognized by T-cells 3 (Fragment)	61804	532	31.20	17	2.88	up
A0A034WBQ5	Alpha-1,4 glucan phosphorylase	97349	845	61.07	50	2.84	up
V9P6U5	Cytochrome P450 monooxygenase 6g2	62018	535	20.19	10	2.84	up
A0A034VJU9	85/88 kDa calcium-independent phospholipase A2	96050	872	23.28	19	2.80	up
A0A034WGV9	Tryptophan--tRNA ligase, cytoplasmic	48047	428	29.44	11	2.79	up
A0A034WMX6	Putative prefoldin subunit 5	18487	160	76.88	10	2.74	up

A0A034W4T9	Ubiquitin carboxyl-terminal hydrolase	110903	1010	16.53	11	2.68	up
A0A034WLW8	Uncharacterized protein MJ1221	81379	736	6.39	4	2.68	up
A0A034VTV6	Glutaredoxin-related protein 5, mitochondrial	18955	169	29.59	4	2.67	up
A0A034VA05	Molybdenum cofactor synthesis protein cinnamon	66329	610	24.59	10	2.63	up
A0A034WIS7	NADH dehydrogenase (Ubiquinone) flavoprotein 1, mitochondrial (Fragment)	52627	480	44.37	17	2.60	up
A0A034W9G1	Dynamin-like 120 kDa protein, mitochondrial	107255	928	30.50	27	2.52	up
A0A034V7P1	DNA polymerase	125024	1098	19.95	19	2.51	up
A0A034VCX6	Ubiquitin carboxyl-terminal hydrolase (Fragment)	146448	1315	12.09	12	2.49	up
A0A034WG96	Putative elongator complex protein 2	93814	830	17.35	11	2.44	up
A0A034WYF0	Peroxiredoxin-2	26204	233	51.93	11	2.43	up
A0A034W5E6	UDP-glucose:glycoprotein glucosyltransferase	176236	1555	38.01	48	2.41	up
A0A034WVN6	Protein SCO1-like protein, mitochondrial	29967	265	31.70	10	2.36	up
A0A034VKN7	Prolyl endopeptidase	84845	738	34.55	23	2.36	up
A0A034WAP1	Beta-ureidopropionase	43439	385	41.30	11	1.98	up
A0A034VHA7	Cullin-3	90145	776	20.88	13	1.87	up
A0A034WAD0	Uncharacterized protein	14800	150	80.00	8	0.58	down
A0A034VSB5	Fasciclin-1	61398	542	35.42	17	0.56	down
A0A034WUJ7	Protein disulfide-isomerase A6 (Fragment)	27335	262	32.06	5	0.56	down
A0A034VPM4	Vinculin	106095	960	50.63	38	0.54	down
A0A034VWR5	Nucleosome-remodeling factor subunit NURF301	247424	2199	7.46	14	0.48	down
A0A034VDN6	Sorting nexin	63684	561	40.11	19	0.45	down
A0A034WBM1	Acetyl-CoA acetyltransferase, cytosolic	41553	394	41.62	11	0.45	down
A0A034V5R2	Talin-1 (Fragment)	211049	1928	48.86	69	0.45	down
A0A034VM61	Clavesin-2	36462	320	33.44	9	0.45	down
A0A034WHJ3	Uncharacterized protein	276170	2345	24.18	52	0.45	down
A0A034W7F8	Nuclear valosin-containing protein-like protein	114062	1043	37.20	29	0.42	down
A0A034VBD0	Putative ATP-dependent RNA helicase DDX5	79548	732	30.60	18	0.42	down
A0A034W1X2	Alaserpin	44489	398	27.39	10	0.42	down
A0A034VRI7	Alpha-actinin, sarcomeric	103820	895	62.35	53	0.42	down
A0A034VUR2	Protein disulfide-isomerase A5	195906	1693	4.13	6	0.42	down
A0A034WHH1	Cytochrome b5	13023	115	54.78	5	0.41	down
A0A034VUP0	Chitinase-like protein Idgf1 (Fragment)	53054	471	15.92	6	0.41	down
A0A034W3J9	Calnexin	69222	616	23.21	12	0.40	down
A0A034W374	Metal transporter CNNM2	105061	947	19.54	14	0.40	down

A0A034VS34	Uncharacterized protein	90376	818	4.28	3	0.39	down
A0A034VUW1	Uncharacterized protein (Fragment)	106552	1022	8.71	7	0.39	down
A0A034W9G7	REST corepressor	50835	458	16.38	7	0.38	down
A0A034VHT6	Malate dehydrogenase (Fragment)	43820	406	16.75	5	0.38	down
A0A034WA29	Aspartate--tRNA ligase, mitochondrial	120485	1077	30.64	29	0.37	down
A0A034W0B4	Chitinase-like protein CG5210 (Fragment)	31320	279	51.25	14	0.37	down
A0A034V322	CD109 antigen	164963	1472	4.82	6	0.36	down
A0A034WSX7	KH domain-containing, RNA-binding, signal transduction-associated protein 2 (Fragment)	37196	322	31.06	11	0.36	down
A0A034WSM6	Guanine nucleotide-binding protein G(S) subunit alpha	44569	382	32.20	10	0.36	down
A0A034WPS6	Inositol-3-phosphate synthase	62553	568	42.96	18	0.36	down
A0A034VP83	Poly(U)-specific endoribonuclease-like protein	71754	675	24.74	13	0.35	down
A0A034W3Z3	Chromodomain Y-like protein	114982	1051	11.04	10	0.35	down
A0A034V2F7	Titin (Fragment)	193385	1716	18.30	21	0.35	down
A0A034V3H3	Nesprin-1 (Fragment)	521689	4618	14.88	56	0.35	down
A0A034WQQ0	Calcyphosin-like protein	24650	219	27.40	6	0.35	down
A0A034WGX8	Uncharacterized protein	36223	320	20.62	6	0.35	down
A0A034VMT8	Gelsolin	84265	745	28.72	18	0.35	down
A0A034VYI5	Non-specific protein-tyrosine kinase	58701	510	27.25	12	0.34	down
A0A034V3I9	Titin (Fragment)	230612	2040	24.71	38	0.34	down
A0A034VP84	Alpha-aminoadipic semialdehyde synthase, mitochondrial	105417	950	6.63	5	0.34	down
A0A034WJE4	Transcription elongation factor SPT5	120771	1090	16.24	13	0.33	down
A0A034VDX0	L-2-hydroxyglutarate dehydrogenase, mitochondrial	50614	455	31.21	10	0.33	down
A0A034W3K4	Myosin-IB	117898	1025	10.15	10	0.32	down
A0A034WJ92	Protein piwi (Fragment)	78854	688	51.45	37	0.32	down
A0A034W5F4	Uncharacterized protein	28374	246	26.02	7	0.32	down
A0A034VPR7	Putative peptidyl-prolyl cis-trans isomerase dodo	17917	160	57.50	11	0.32	down
A0A034VW38	SUN domain-containing protein 1 (Fragment)	67523	594	21.55	13	0.31	down
A0A034WKG9	Ecdysteroid UDP-glucosyltransferase	62370	549	24.77	10	0.31	down
A0A034VK06	Dystonin (Fragment)	566833	4968	12.32	52	0.31	down
A0A034W4V5	DE-cadherin	178717	1580	14.75	22	0.31	down
A0A034V946	Src substrate cortactin (Fragment)	67412	601	16.97	9	0.30	down
A0A034W0P6	Serine/arginine-rich splicing factor 7	29213	261	17.62	4	0.30	down
A0A034WN98	17-beta-hydroxysteroid dehydrogenase 13	36367	326	25.15	7	0.30	down
A0A034VZK0	Muscle M-line assembly protein unc-89	372226	3274	12.43	33	0.30	down

A0A034VWG7	2-amino-3-ketobutyrate coenzyme A ligase, mitochondrial	47607	435	42.53	15	0.29	down
A0A034W8P6	Protein E(Sev)2B	24462	211	43.60	9	0.29	down
A0A034VQJ6	Uncharacterized protein	66971	622	15.76	6	0.29	down
A0A034WHD8	Structural maintenance of chromosomes protein	159829	1389	7.49	10	0.29	down
A0A034WT25	Uncharacterized protein	31077	274	43.80	8	0.29	down
A0A034VAC6	Protein elav	47180	446	35.65	12	0.29	down
A0A034V2K7	Spectrin beta chain, non-erythrocytic 5	483273	4200	26.48	94	0.29	down
A0A034VQY4	Protein bric-a-brac 2	101626	929	17.01	11	0.28	down
A0A034WBA7	Putative G-protein coupled receptor 158	94535	809	29.05	20	0.28	down
A0A034W1D4	Hepatoma-derived growth factor	61550	545	30.09	15	0.28	down
A0A034W317	Uncharacterized protein	84817	798	20.93	14	0.27	down
A0A034VVP8	Serpin B9	56716	504	29.76	14	0.27	down
M1F3Z9	Glutathione S-transferase omega-1	29161	255	46.67	11	0.26	down
A0A034V6M3	60S ribosomal protein L23a	28521	266	26.32	10	0.26	down
A0A034WRQ2	Alpha-parvin	42015	366	33.06	10	0.26	down
A0A034VDE1	Protein-tyrosine-phosphatase	172176	1506	3.78	5	0.26	down
A0A034VQG5	Nucleoprotein TPR	271019	2410	26.22	53	0.26	down
A0A034WF58	Glutathione peroxidase	30583	278	48.92	13	0.26	down
A0A034V9K3	Ankyrin repeat domain-containing protein 39	20553	181	43.65	6	0.25	down
A0A034V5V1	ZNF domain containing Pogo transposable element	147005	1326	16.14	18	0.25	down
A0A034VL96	Kin of IRRE-like protein 2 (Fragment)	50688	454	10.79	5	0.25	down
A0A034WSZ7	Heterochromatin protein 1	23688	212	23.11	5	0.24	down
A0A034W3W4	Calponin	19151	169	71.01	13	0.24	down
A0A034V7N7	Protein lethal(2) giant larvae	127930	1176	24.23	21	0.24	down
A0A034WHN3	Putative helicase MOV-10	94691	826	40.68	32	0.24	down
A0A034V457	Prolow-density lipoprotein receptor-related protein 1 (Fragment)	515158	4603	3.13	13	0.24	down
A0A034VZ39	Peroxidasin	136185	1202	18.89	19	0.24	down
A0A034VTK4	DNA topoisomerase 2	167839	1481	42.13	59	0.24	down
A0A034VFW9	Uncharacterized protein (Fragment)	50113	443	24.83	7	0.23	down
A0A034VR52	Procollagen-lysine,2-oxoglutarate 5-dioxygenase 1	85847	743	24.23	18	0.23	down
A0A034VIH5	Protein lap4 (Fragment)	83204	783	17.50	9	0.23	down
A0A034WJ51	Nucleosomal histone kinase 1	70264	637	13.66	7	0.23	down
A0A034V413	Nesprin-1	915393	7973	19.68	119	0.23	down
A0A034V975	Uncharacterized protein	75107	674	30.42	18	0.22	down

A0A034V1E0	Cell surface glycoprotein 1	139929	1257	15.27	17	0.21	down
A0A034VYW9	Contactin	158548	1393	25.20	27	0.21	down
A0A034VKE6	Bifunctional methylenetetrahydrofolate dehydrogenase/cyclohydrolase, mitochondrial	34038	316	32.59	8	0.21	down
A0A034VW09	Protein Skeletor, isoforms B/C	82875	741	29.82	14	0.21	down
A0A034WY02	Pyridoxine-5'-phosphate oxidase	28409	245	24.08	7	0.21	down
A0A034V5M7	Uncharacterized protein (Fragment)	107664	923	18.09	15	0.20	down
A0A034VY88	Protein hu-li tai shao	76611	698	69.63	35	0.20	down
A0A034WTE5	Trans-1,2-dihydrobenzene-1,2-diol dehydrogenase	37022	332	35.24	13	0.20	down
A0A034WKG7	Epidermal retinol dehydrogenase 2	35786	317	3.47	1	0.20	down
A0A034VCF3	Elongation factor 1-alpha	45374	414	64.25	22	0.20	down
A0A034UZS9	PDZ and LIM domain protein Zasp	52978	495	22.22	9	0.19	down
A0A034WI29	Protein lin-7-like protein B	21479	195	28.72	5	0.19	down
A0A034WM22	Multiple inositol polyphosphate phosphatase 1	54124	467	13.70	6	0.19	down
A0A034V5Q1	Ras suppressor protein 1 (Fragment)	15927	138	39.86	5	0.19	down
A0A034W1E0	Lachesin	41651	377	23.61	6	0.19	down
A0A034VDJ6	Putative epidermal cell surface receptor	118741	1070	8.69	7	0.19	down
A0A034VUS9	Dihydropyrimidine dehydrogenase (NADP(+))	113562	1047	5.83	5	0.19	down
A0A034WWI9	Ejaculatory bulb-specific protein 3	14702	127	22.05	3	0.18	down
A0A034WGR6	Uncharacterized protein	26306	231	38.53	6	0.18	down
A0A034VIA8	Cytochrome P450 4g1 (Fragment)	55770	488	22.95	10	0.18	down
A0A034VJ94	Glucose dehydrogenase (Acceptor)	68918	620	11.77	6	0.18	down
A0A034WT83	Alpha-tocopherol transfer-like protein	33631	287	19.86	4	0.17	down
A0A034VZF3	Putative RNA-binding protein 15B	56628	507	24.65	13	0.17	down
Q86QQ0	Glutathione S-transferase	23756	209	37.32	6	0.17	down
A0A034V2D5	Neurogenic locus notch-like protein 1 (Fragment)	446774	4167	1.46	5	0.17	down
A0A034VSX0	Phosphate-regulating neutral endopeptidase	79759	683	22.69	13	0.16	down
A0A034VYF9	Paramyosin, long form	102321	879	53.70	41	0.16	down
A0A034WEH8	SH3 domain-binding glutamic acid-rich protein-like protein (Fragment)	38305	346	16.18	5	0.16	down
A0A034VA84	Uncharacterized protein	57231	502	22.91	10	0.16	down
A0A034VTB7	Uncharacterized protein	47334	421	42.76	12	0.16	down
A0A034VTK3	Chitinase-like protein Idgf5	48605	432	27.31	9	0.16	down
A0A034VPA5	Heterogeneous nuclear ribonucleoprotein Q (Fragment)	47111	417	21.34	9	0.15	down

A0A034VDP0	Obscurin (Fragment)	70619	631	30.74	13	0.15	down
A0A034VRQ6	Protein held out wings	39076	356	37.64	11	0.15	down
A0A034WL14	RING finger protein unkempt (Fragment)	69020	604	12.91	5	0.15	down
A0A034VWI9	General odorant-binding protein 99a (Fragment)	20372	176	18.18	4	0.15	down
A0A034WVT6	Membrane metallo-endopeptidase-like 1 (Fragment)	82524	714	22.13	14	0.15	down
A0A034WRI9	Sarcocystatin-A	13543	125	47.20	6	0.15	down
A0A034UZB9	Sushi, von Willebrand factor type A, EGF and pentraxin domain-containing protein 1 (Fragment)	127179	1152	7.46	6	0.14	down
A0A034V3K5	CUB and sushi domain-containing protein 1 (Fragment)	96461	887	14.99	10	0.14	down
A0A034VE41	Twitchin (Fragment)	556499	4978	23.54	86	0.14	down
A0A034VJJ7	LIM and SH3 domain protein Lasp (Fragment)	22613	198	28.79	7	0.14	down
A0A034W9N3	Heterogeneous nuclear ribonucleoprotein 87F	39189	368	40.22	18	0.14	down
A0A034WAI7	Non-specific serine/threonine protein kinase	64369	571	16.81	7	0.14	down
A0A034WML8	Protein takeout	30874	269	20.07	4	0.14	down
A0A034V520	Uncharacterized protein	65301	610	16.89	10	0.14	down
A0A034WSA4	Methionine adenosyltransferase 2 subunit beta	42268	372	22.85	6	0.14	down
A0A034W4D3	L-xylulose reductase	25972	241	28.63	7	0.14	down
A0A034WI17	ATP-binding cassette sub-family A member 3	36412	320	15.31	4	0.14	down
A0A034V2S6	Uncharacterized protein	105991	944	10.70	7	0.14	down
A0A034W6R3	Tissue inhibitor of metalloproteases	23428	210	15.71	5	0.14	down
A0A034W8L1	Flotillin-2	46568	425	43.53	17	0.13	down
A0A034WCQ3	Carbonic anhydrase 15 (Fragment)	39936	350	9.71	3	0.13	down
A0A034VUS5	Integrin-linked protein kinase	50786	448	32.37	14	0.13	down
A0A034W6I6	Amidophosphoribosyltransferase	57877	532	14.85	7	0.13	down
A0A034VND6	Tropomyosin-1, isoforms 33/34 (Fragment)	32457	282	48.58	20	0.13	down
A0A034VQZ0	Zinc finger protein 512B	44855	427	26.23	7	0.13	down
W6A0Y6	Interferon-inducible double stranded RNA-dependent protein kinase activator A	32936	292	17.81	4	0.13	down
A0A034WP07	Attacin-A	25021	239	72.80	14	0.13	down
I1T1H2	Heat shock protein 20	18932	170	67.65	10	0.13	down
A0A034V5I2	Muscle M-line assembly protein unc-89 (Fragment)	213521	1922	17.90	27	0.13	down
A0A034V7Q2	Protein yellow (Fragment)	29702	261	31.42	6	0.13	down
A0A034VKK9	Alcohol dehydrogenase (NADP(+))	36548	324	38.89	13	0.13	down
A0A034VWH3	Myosin regulatory light chain 2	23610	222	52.70	10	0.12	down

A0A034VC57	Peroxidasin-like protein	155580	1384	11.49	13	0.12	down
A0A034V8W7	Cytochrome P450 4g15	55074	484	23.76	10	0.11	down
A0A034V4U6	Integrin alpha-PS2 (Fragment)	132072	1205	13.03	13	0.11	down
A0A034WR79	Uncharacterized protein	24755	214	59.35	13	0.11	down
A0A034WHU4	Delta-1-pyrroline-5-carboxylate synthase	83870	773	19.79	13	0.11	down
A0A034V1Z3	Atlastin (Fragment)	91611	791	48.93	37	0.11	down
A0A034VDB5	Protein lap4 (Fragment)	101687	915	20.11	14	0.11	down
A0A034VQ98	Arylphorin subunit C223 (Fragment)	47837	407	1.97	1	0.11	down
A0A034WGA0	Kin of IRRE-like protein 2 (Fragment)	68865	620	5.64	4	0.11	down
A0A034VR22	Galectin	20799	179	22.35	4	0.11	down
A0A034W237	Esterase B1	64331	566	14.49	6	0.10	down
A0A034VPE5	Tropomyosin-1, isoforms 9A/A/B (Fragment)	21903	188	69.68	19	0.10	down
A0A034WAU8	Uncharacterized protein	73989	755	7.81	4	0.10	down
A0A034WAX0	Collagen alpha-1(XV) chain	124401	1184	8.53	10	0.10	down
A0A034VHE3	Gamma-glutamyltranspeptidase 1	79367	738	14.63	10	0.09	down
A0A034VSE8	Serrate RNA effector molecule-like protein	113786	997	19.16	17	0.09	down
A0A034WF27	TPPP family protein CG4893	21493	201	40.30	7	0.09	down
A0A034V519	Endoplasmic reticulum metallopeptidase 1 (Fragment)	33550	293	13.99	4	0.09	down
A0A034W4R7	Alpha,alpha-trehalose-phosphate synthase (UDP-forming) A	91999	813	33.46	24	0.09	down
A0A034VHH3	Laminin subunit alpha-1 (Fragment)	188941	1677	10.26	14	0.09	down
A0A034WS42	Histone H4	11382	103	65.05	15	0.08	down
A0A034V4T6	Fasciclin-2 (Fragment)	57857	520	45.19	17	0.08	down
A0A034VAC8	Uncharacterized protein	95118	885	8.93	8	0.08	down
A0A034VI82	Tropomodulin	43782	386	34.97	10	0.08	down
A0A034VQI8	Uncharacterized protein (Fragment)	72191	683	33.82	17	0.08	down
A0A034W3W2	Regucalcin	32384	296	42.91	12	0.08	down
A0A034VIV7	Uncharacterized protein (Fragment)	15284	132	12.88	2	0.08	down
A0A034V2U0	Protein 4.1-like protein	185190	1703	57.37	82	0.07	down
A0A034VQR8	Troponin T, skeletal muscle	45988	384	39.32	22	0.07	down
A0A034VJG4	Neurotactin	95358	871	9.87	6	0.07	down
A0A034VSZ7	Uncharacterized protein	26073	229	47.16	13	0.07	down
A0A034VE17	Uncharacterized protein	27887	240	26.67	6	0.07	down
A0A034VCC3	Filamin-B (Fragment)	124654	1130	5.93	7	0.07	down
A0A034VZ11	Uncharacterized protein (Fragment)	21843	231	27.71	5	0.07	down
A0A034VR92	Tropomyosin-2	32743	284	57.04	17	0.06	down
A0A034W4Q0	Larval cuticle protein A2B (Fragment)	18617	180	73.89	17	0.06	down
A0A034WSV2	Lamin-C	70077	622	56.27	37	0.06	down

A0A034VUV3	Lamin Dm0	69746	617	71.64	46	0.06	down
A0A034VT34	Angiopoietin-4	52090	454	20.04	7	0.06	down
A0A034VIW3	Homer protein-like protein 2	42349	389	19.02	6	0.06	down
A0A034VXK4	Uncharacterized protein (Fragment)	44748	431	59.63	16	0.06	down
A0A034W9Y1	Larval cuticle protein A2B	24482	250	92.40	19	0.06	down
A0A034VII9	Inhibitor of growth protein	44693	413	13.56	4	0.06	down
A0A034VU29	Regucalcin	39454	351	64.96	26	0.05	down
A0A034WHZ8	Uncharacterized protein (Fragment)	53199	464	21.77	12	0.05	down
A0A034VM08	Uncharacterized protein	62881	556	59.71	40	0.05	down
A0A034V442	Protein no-on-transient A	78331	691	34.15	23	0.05	down
A0A034WFT6	Uncharacterized protein	19920	195	59.49	14	0.05	down
A0A034WXP8	Uncharacterized protein	16783	153	9.15	2	0.04	down
A0A034VWF5	Basement membrane-specific heparan sulfate proteoglycan core protein (Fragment)	102363	922	34.27	29	0.04	down
A0A034V3B3	Arylphorin subunit A4 (Fragment)	140432	1224	45.92	55	0.04	down
A0A034WSW4	Larval cuticle protein 8	11478	104	22.12	2	0.04	down
A0A034V5B7	Cuticle protein 19	16513	147	17.01	2	0.04	down
A0A034VQS9	Mobility group protein 1A	12648	113	39.82	7	0.04	down
A0A034WL03	Development-specific 25 kDa protein	28860	259	69.50	19	0.04	down
A0A034W585	Larval cuticle protein A3A	14397	138	75.36	17	0.04	down
A0A034V3D6	J domain-containing protein	19631	177	70.62	15	0.03	down
A0A034WXX0	Histone H1	24996	240	30.83	11	0.03	down
A0A034VYM4	Larval serum protein 1 gamma chain (Fragment)	92074	773	31.31	27	0.03	down
A0A034UX56	Trehalose 6-phosphate phosphatase	31189	274	81.75	25	0.03	down
A0A034VC00	Collagen alpha-1(IV) chain	185541	1833	13.04	21	0.03	down
A0A034W7C2	Uncharacterized protein	26739	235	43.83	10	0.03	down
A0A034WJH9	Uncharacterized protein	37316	332	6.93	2	0.03	down
A0A034W347	Uncharacterized protein	23274	224	8.48	1	0.03	down
A0A034V6N3	High mobility group protein Z	12805	112	33.04	5	0.02	down
A0A034WJQ4	Fat-body protein 1 (Fragment)	58417	511	48.53	31	0.02	down
A0A034VBG9	Uncharacterized protein	23773	229	65.07	16	0.02	down

Table S4. Proteins highly abundant in 6- and 9-day-old ovaries compared to 1-day-old ovaries of *Bactrocera dorsalis*.

Accession Number	Annotation	Protein Length	Sequence Coverage	Peptides	Ov-6/Ov-1	Ov-9/Ov-1	Ov-9/Ov-6[a, b]
A0A034WHP3	Vitelline membrane protein Vm26Aa	114	82.46	16	↑ 35.99 ± 3.19	↑ 35.57 ± 1.05	0.99 ± 0.15 b
A0A034WY57	Putative ATP-dependent RNA helicase me31b	457	77.46	46	↑ 29.41 ± 2.41	↑ 32.45 ± 8.44	↑ 1.64 ± 0.24 a
A0A034VQJ3	Vitellogenin-1	436	83.26	82	↑ 26.95 ± 1.34	↑ 29.72 ± 2.42	1.4 ± 0.02 b
A0A034WRE4	60S ribosomal protein L30	111	60.36	9	↑ 26.93 ± 0.51	↑ 23.9 ± 3.95	0.73 ± 0.07 b
A0A034WWC1	General odorant-binding protein 99b	143	49.65	10	↑ 26.43 ± 3.09	↑ 19.36 ± 11.46	0.8 ± 0.11 b
Q8T7E3	Vitellogenin 1	436	83.26	77	↑ 23.51 ± 3.1	↑ 21.25 ± 11.65	0.93 ± 0.04 b
A0A034VP94	40S ribosomal protein S12	139	84.17	16	↑ 22.03 ± 1.71	↑ 15.62 ± 3.41	↓ 0.66 ± 0.01 a
A0A034W432	Cytochrome P450 306a1	545	40.92	19	↑ 20.86 ± 2.93	↑ 15.43 ± 6	↓ 0.67 ± 0 a
A0A034V196	Protein disulfide-isomerase	135	51.85	6	↑ 20.18 ± 1.58	↑ 23.15 ± 1.28	1.34 ± 0.21 b
A0A034WNR3	46 kDa FK506-binding nuclear protein	180	28.89	4	↑ 19.46 ± 4.11	↑ 19.57 ± 8.2	1 ± 0.15 b
A0A034W6L5	Importin-7	1048	37.6	31	↑ 17.87 ± 1.85	↑ 14.03 ± 2.7	0.73 ± 0.12 b
A0A034VFV2	6-phosphogluconate dehydrogenase, decarboxylating	481	63.2	27	↑ 16.85 ± 0.85	↑ 15.02 ± 4.58	1 ± 0.19 b
I1SWI8	Heat shock protein 60	573	77.66	47	↑ 15.44 ± 0.78	↑ 12.15 ± 2.23	0.81 ± 0.04 b
A0A034V6V9	Importin-4	1087	42.23	40	↑ 14.62 ± 1.49	↑ 13.56 ± 3.1	1.05 ± 0.16 b
A0A034VJ67	Protein cup	1173	22.25	24	↑ 14.35 ± 0.54	↑ 13.94 ± 1.59	1.03 ± 0.12 b
A0A034VV64	Eukaryotic translation initiation factor 3 subunit A	1126	34.46	37	↑ 13.06 ± 2.83	↑ 7.47 ± 4.55	↓ 0.51 ± 0 a
A0A034VE18	Heat shock protein 23	170	78.82	15	↑ 12.97 ± 1.84	↑ 29.02 ± 8.22	↑ 3.59 ± 0.02 a
A0A034VR21	Protein bicaudal C	899	26.25	15	↑ 12.51 ± 0.31	↑ 9.06 ± 2.5	0.72 ± 0.04 b
A0A034VXK9	Aspartate aminotransferase	376	76.86	26	↑ 12.49 ± 0.75	↑ 10.59 ± 4.02	0.98 ± 0.17 b
A0A034V1Z6	Valine--tRNA ligase	1054	39.75	36	↑ 11.94 ± 3.05	↑ 11.89 ± 5.67	1.05 ± 0.15 b
A0A034VMA5	ATP-binding cassette sub-family F member 2	610	27.7	17	↑ 11.71 ± 0.72	↑ 10.91 ± 2.25	0.94 ± 0.16 b
A0A034W4L6	Lipid storage droplets surface-binding protein 2	353	61.47	20	↑ 11.61 ± 0.62	↑ 11.82 ± 1.96	1.1 ± 0.2 b

A0A034W0T2	Inosine-5'-monophosphate dehydrogenase	534	35.96	12	↑	11.42 ± 1.58	↑ 13.26 ± 3.8	1.24 ± 0.23 b
A0A034W2M7	Elongation factor 1-gamma	438	58.68	31	↑	11.41 ± 2.71	↑ 10.04 ± 4.76	0.96 ± 0.18 b
A0A034WB81	T-complex protein 1 subunit gamma	543	71.27	41	↑	10.98 ± 2.22	↑ 9.44 ± 3.66	0.88 ± 0.13 b
A0A034WWD8	Exportin-1	1070	40.56	37	↑	10.85 ± 2.08	↑ 7.83 ± 3.84	0.7 ± 0.09 b
A0A034V8K3	RING finger protein 17	675	34.81	17	↑	10.68 ± 2.84	↑ 9.17 ± 5.08	0.79 ± 0.15 b
A0A034WNG6	Alanine--tRNA ligase, cytoplasmic	967	50.26	38	↑	10.57 ± 1.43	↑ 11.06 ± 2.41	1.12 ± 0.06 b
A0A034WKN9	C2 domain-containing protein 3	682	33.43	14	↑	10.37 ± 1.67	↑ 11.82 ± 3.74	1.29 ± 0.24 b
A0A034WL96	60S ribosomal protein L13	216	39.81	13	↑	10.27 ± 0.47	↑ 8.08 ± 0.39	0.76 ± 0.03 b
A0A034VA13	Eukaryotic initiation factor 4A	403	67	28	↑	10.2 ± 0.86	↑ 8.02 ± 1.28	0.77 ± 0.11 b
A0A034W486	Protein transport protein Sec61 subunit alpha isoform 2	340	20.29	7	↑	10.07 ± 1.6	↑ 9.94 ± 3.59	0.97 ± 0.08 b
A0A034WBZ8	Putative serine protease K12H4.7	517	39.65	19	↑	10.02 ± 0.39	↑ 13.4 ± 3.28	↑ 1.53 ± 0.21 a
A0A034WGZ4	Elongation factor Ts, mitochondrial	305	40.66	9	↑	9.68 ± 1.04	↑ 8.81 ± 1.01	0.93 ± 0.07 b
A0A034VUT0	Protein singed	509	31.43	13	↑	9.29 ± 1.67	↑ 12.32 ± 4.27	1.43 ± 0.09 b
A0A034VYI4	Tyrosine--tRNA ligase	523	65.01	29	↑	9.25 ± 1.3	↑ 7.46 ± 3.3	0.81 ± 0.17 b
A0A034VZW3	Transitional endoplasmic reticulum ATPase TER94	799	65.08	52	↑	9.2 ± 0.77	↑ 8.87 ± 3.86	1.14 ± 0.12 b
A0A034VUQ7	Glucose-6-phosphate 1-dehydrogenase	527	52.56	22	↑	9.14 ± 0.14	↑ 7.12 ± 0.85	0.78 ± 0.13 b
A0A034WJ52	Eukaryotic translation initiation factor 4 gamma 1	1766	36.98	46	↑	9.09 ± 0.78	↑ 5.18 ± 2.11	↓ 0.56 ± 0.04 a
A0A034VLP4	Transforming growth factor-beta-induced protein ig-h3	810	44.69	28	↑	9.02 ± 0.7	↑ 12 ± 4.83	↑ 1.65 ± 0.19 a
A0A034WCS4	Dehydrogenase/reductase SDR family member 11	251	57.77	8	↑	8.92 ± 1.42	↑ 12.22 ± 4.56	1.5 ± 0.02 b
A0A034W3A2	Serine--tRNA ligase, cytoplasmic	497	49.09	17	↑	8.51 ± 0.47	↑ 6.37 ± 2.77	0.81 ± 0.04 b
A0A034VC07	Thioredoxin reductase 1, mitochondrial	493	71.4	28	↑	8.14 ± 1.09	↑ 12.01 ± 3.32	↑ 1.76 ± 0.03 a
A0A034VUM4	Protein extra bases	420	46.19	23	↑	8 ± 0.54	↑ 5.47 ± 2.25	0.69 ± 0.11 b
A0A034W1V3	Diphthine synthase	280	47.14	9	↑	8 ± 0.7	↑ 12.99 ± 1.25	↑ 1.67 ± 0.07 a
A0A034VDY8	Bifunctional glutamate/proline--tRNA ligase	1636	48.66	65	↑	7.8 ± 1.74	↑ 5.35 ± 2.86	0.68 ± 0 b

A0A034VUA6	26S proteasome non-ATPase regulatory subunit 1	1017	52.51	45	↑ 7.42 ± 1.44	↑ 10.15 ± 3.44	↑ 1.58 ± 0.2 a
A0A034WE93	Glycine--tRNA ligase	679	50.07	31	↑ 6.98 ± 0.61	↑ 8.36 ± 1.99	1.33 ± 0.15 b
A0A034V173	Putative N-acetyltransferase san	186	50.54	10	↑ 6.88 ± 0.62	↑ 7.46 ± 1.48	1.12 ± 0.09 b
A0A034WPA4	28S ribosomal protein S35, mitochondrial	332	31.02	8	↑ 6.87 ± 1.65	↑ 6.28 ± 3.28	0.89 ± 0.1 b
A0A034V1D4	CAD protein	999	28.43	25	↑ 6.61 ± 0.15	↑ 6.88 ± 1.03	1.06 ± 0.17 b
A0A034VEZ1	Glutamate synthase (NADH), amyloplastic	2093	43.91	77	↑ 6.5 ± 1.71	↑ 6.03 ± 3.38	0.99 ± 0.19 b
A0A034WKN7	Methyltransferase-like protein 13	675	33.63	19	↑ 6.45 ± 0.98	↑ 9.51 ± 2.55	↑ 1.53 ± 0.02 a
A0A034W4W3	Transportin-1	886	27.2	18	↑ 6.4 ± 1.29	↑ 5.22 ± 2.18	0.79 ± 0.06 b
A0A034VWI5	Leucine-rich PPR motif-containing protein, mitochondrial	1418	43.86	50	↑ 6.35 ± 0.52	↑ 5.75 ± 1.15	0.88 ± 0.06 b
A0A034WM27	Tubulin--tyrosine ligase-like protein 12	641	29.49	17	↑ 6.28 ± 0.11	↑ 8.49 ± 1.11	1.43 ± 0.12 b
A0A034WMH5	26S proteasome non-ATPase regulatory subunit 13	382	47.64	20	↑ 6.22 ± 0.99	↑ 8.79 ± 2.28	1.49 ± 0.01 b
A0A034VBA8	Isoleucine--tRNA ligase, cytoplasmic	468	38.46	16	↑ 6.16 ± 0.75	↑ 5.54 ± 1.74	0.96 ± 0.17 b
A0A034WM52	Cullin-associated NEDD8-dissociated protein 1	1236	39.56	41	↑ 6.03 ± 0.99	↑ 8.77 ± 2.69	↑ 1.64 ± 0 a
A0A034W3T2	Serine/threonine-protein phosphatase 5	544	40.26	19	↑ 6.02 ± 0.29	↑ 4.56 ± 0.57	0.78 ± 0.09 b
A0A034VRQ0	Translational activator GCN1	1471	34.81	39	↑ 5.98 ± 1.51	↑ 4.44 ± 2.26	0.73 ± 0 b
A0A034VMW4	Uridine 5'-monophosphate synthase	487	49.08	20	↑ 5.9 ± 0.64	↑ 4.35 ± 1.6	0.75 ± 0.16 b
A0A034WF61	Serine/threonine-protein phosphatase	303	36.96	9	↑ 5.86 ± 0.93	↑ 6.1 ± 1.99	1.06 ± 0.08 b
A0A034W9M9	Cytosolic 10-formyltetrahydrofolate dehydrogenase	231	47.19	9	↑ 5.84 ± 0.65	↑ 3.49 ± 0.24	↓ 0.59 ± 0.07 a
A0A034UZ41	Sepiapterin reductase	263	50.57	10	↑ 5.82 ± 0.33	↑ 8.2 ± 0.52	1.5 ± 0.04 b
A0A034V3I0	CAD protein	1234	20.83	17	↑ 5.81 ± 0.64	↑ 8.12 ± 1.65	1.47 ± 0.01 b
A0A034WEU3	Enoyl-CoA delta isomerase 2, mitochondrial	260	48.46	10	↑ 5.7 ± 0.08	↑ 9.78 ± 0.27	↑ 1.85 ± 0.01 a
A0A034WFJ7	Putative elongator complex protein 1	1025	26.63	23	↑ 5.69 ± 0.77	↑ 4.91 ± 1.84	0.87 ± 0.06 b
A0A034VFZ9	Kinetochore-associated protein 1	770	26.36	17	↑ 5.67 ± 1.25	↑ 6.74 ± 2.88	1.22 ± 0.13 b
A0A034VLF2	Coatomer subunit beta	953	33.47	26	↑ 5.6 ± 0.25	↑ 6.38 ± 0.51	1.14 ± 0.08 b
A0A034V3E5	Nuclear pore complex protein Nup107	899	34.59	25	↑ 5.55 ± 1.61	↑ 8.46 ± 4.5	↑ 1.67 ± 0 a

A0A034VAK1	Heat shock protein 75 kDa, mitochondrial	710	42.25	25	↑ 5.53 ± 0.63	↑ 5.33 ± 1.21	0.97 ± 0.1 b
A0A034WIL4	NADH dehydrogenase (Ubiquinone) iron-sulfur protein 2, mitochondrial	473	43.97	18	↑ 5.45 ± 1.07	↑ 5.08 ± 2.41	0.94 ± 0.12 b
A0A034VKN3	Leucine--tRNA ligase, cytoplasmic	1182	45.18	44	↑ 5.32 ± 1.31	↑ 5.4 ± 2.71	1.02 ± 0.19 b
A0A034WDL1	NADH-ubiquinone oxidoreductase 75 kDa subunit, mitochondrial	736	46.47	29	↑ 5.25 ± 0.2	↑ 4.72 ± 0.27	0.86 ± 0.17 b
A0A034WUW3	UPF0160 protein MYG1, mitochondrial	385	37.14	12	↑ 5.24 ± 1.15	↑ 4.61 ± 2.03	0.86 ± 0.12 b
A0A034V3F2	26S protease regulatory subunit 7	433	62.82	30	↑ 5.19 ± 0.57	↑ 7.91 ± 1.29	↑ 1.64 ± 0.18 a
A0A034W1C0	Eukaryotic translation initiation factor 4 gamma 2	942	27.81	19	↑ 5.18 ± 0.23	↑ 7.82 ± 1.63	↑ 1.64 ± 0.02 a
A0A034VKX9	Alkyldihydroxyacetonephosphate synthase	689	22.35	11	↑ 5.12 ± 0.67	↑ 5.15 ± 1.26	1.03 ± 0.08 b
A0A034VM32	CCR4-NOT transcription complex subunit 1	1091	20.99	20	↑ 5.06 ± 1.42	↑ 5.82 ± 3.17	1.27 ± 0.25 b
A0A034W6I8	Exportin-2	972	34.88	33	↑ 5.02 ± 1.3	↑ 5.94 ± 3.07	1.24 ± 0.24 b
A0A034WU84	Proteasome subunit alpha type	253	60.08	14	↑ 4.93 ± 0.37	↑ 6.49 ± 1.28	1.33 ± 0.09 b
A0A034W3L0	Ankyrin repeat and KH domain-containing protein mask	507	29.98	11	↑ 4.84 ± 0.16	↑ 7.83 ± 0.62	↑ 1.68 ± 0.02 a
A0A034W9B6	T-complex protein 1 subunit zeta	531	64.22	28	↑ 4.79 ± 0.6	↑ 4.09 ± 1.02	0.86 ± 0.13 b
A0A034VJH4	N-alpha-acetyltransferase 16, NatA auxiliary subunit	878	33.94	28	↑ 4.76 ± 0.56	↑ 6.26 ± 1.57	1.33 ± 0.06 b
A0A034WFC0	ATP synthase subunit gamma	297	38.72	12	↑ 4.64 ± 0.42	↑ 6.08 ± 1.36	1.35 ± 0.1 b
A0A034VH61	Putative aminopeptidase W07G4.4	505	59.8	27	↑ 4.58 ± 0.59	↑ 7.23 ± 2.82	↑ 1.78 ± 0.09 a
A0A034W9F2	Histone-lysine N-methyltransferase setd3	534	36.14	17	↑ 4.57 ± 1.01	↑ 4.37 ± 1.91	0.95 ± 0.09 b
A0A034WNJ6	Heat shock 70 kDa protein cognate 5	679	55.23	34	↑ 4.47 ± 0.62	↑ 3.91 ± 0.94	0.89 ± 0.21 b
A0A034WPS5	COP9 signalosome complex subunit 1b	511	25.24	10	↑ 4.35 ± 0.68	↑ 6.12 ± 1.85	↑ 1.51 ± 0.22 a
A0A034WVA3	Elongation factor Tu	490	49.39	19	↑ 4.26 ± 0.6	↑ 3.53 ± 1.64	0.84 ± 0.21 b
A0A034VBM4	Trifunctional purine biosynthetic protein adenosine-3	1368	35.75	39	↑ 4.25 ± 0.48	↑ 3.96 ± 0.49	0.96 ± 0.2 b
A0A034V9G7	Eukaryotic translation initiation factor 5B	1215	24.86	20	↑ 4.16 ± 0.39	↑ 4.36 ± 0.73	1.05 ± 0.22 b
A0A034VGJ0	Putative multidrug resistance-associated protein lethal(2)03659	513	21.64	9	↑ 4.15 ± 0.64	↑ 5.91 ± 1.77	↑ 1.56 ± 0.15 a
A0A034VX54	Aspartate aminotransferase, cytoplasmic	407	60.69	20	↑ 4.06 ± 0.43	↑ 4.41 ± 1.71	1.14 ± 0.16 b

A0A034WRS4	60S acidic ribosomal protein P2	113	74.34	9	↑ 4.04 ± 0.42	↑ 3.31 ± 0.87	0.82 ± 0.18 b
A0A034V6T2	Insulin-degrading enzyme	1048	24.43	24	↑ 3.94 ± 0.43	↑ 3.65 ± 1.61	0.99 ± 0.22 b
A0A034VZH7	26S proteasome non-ATPase regulatory subunit 2	911	48.74	35	↑ 3.93 ± 0.59	↑ 6.17 ± 1.55	↑ 1.65 ± 0.03 a
A0A034WU78	Fumarate hydratase, mitochondrial	500	48	18	↑ 3.87 ± 0.38	↑ 5.49 ± 1.09	1.44 ± 0.07 b
A0A034VAS4	Acetyltransferase component of pyruvate dehydrogenase complex	503	39.36	14	↑ 3.79 ± 0.69	↑ 3.31 ± 1.25	0.9 ± 0.11 b
A0A034WAN7	Signal recognition particle 54 kDa protein	508	36.22	12	↑ 3.76 ± 0.76	↑ 3.09 ± 1.32	0.8 ± 0.13 b
A0A034W1H3	Nuclear pore complex protein Nup93	809	38.32	27	↑ 3.68 ± 0.72	↑ 4 ± 1.52	1.19 ± 0.09 b
A0A034V4D6	DnaJ-like protein subfamily C member 13	1936	21.02	31	↑ 3.66 ± 0.6	↑ 5 ± 1.41	1.42 ± 0 b
A0A034VEP3	4-aminobutyrate aminotransferase, mitochondrial	491	48.88	18	↑ 3.64 ± 0.09	↑ 5.56 ± 0.28	↑ 1.57 ± 0.01 a
A0A034WGV9	Tryptophan--tRNA ligase, cytoplasmic	428	29.44	11	↑ 3.46 ± 0.16	↑ 2.79 ± 0.31	0.82 ± 0.15 b
A0A034WJS4	Protein l(2)37Cc	276	85.14	21	↑ 3.38 ± 0.28	↑ 4.24 ± 1.04	1.26 ± 0.2 b
A0A034VNQ2	Nuclear pore complex protein Nup85	677	36.63	19	↑ 3.38 ± 0.63	↑ 4.96 ± 1.77	1.47 ± 0.18 b
A0A034VDF3	Squamous cell carcinoma antigen recognized by T-cells 3	532	31.2	17	↑ 3.34 ± 0.17	↑ 2.88 ± 0.26	0.84 ± 0.08 b
A0A034VXN0	Short/branched chain specific acyl-CoA dehydrogenase, mitochondrial	413	41.16	12	↑ 3.3 ± 0.04	↑ 2.94 ± 0.28	0.87 ± 0.05 b
A0A034VG90	Nuclear pore complex protein Nup133	1193	20.12	19	↑ 3.23 ± 0.6	↑ 3.54 ± 1.18	1.23 ± 0.04 b
A0A034VQ24	60S ribosomal export protein NMD3	517	30.95	12	↑ 3.23 ± 0.21	↑ 3.61 ± 0.6	1.14 ± 0.18 b
A0A034WIS7	NADH dehydrogenase (Ubiquinone) flavoprotein 1, mitochondrial	480	44.37	17	↑ 3.1 ± 0.13	↑ 2.6 ± 0.1	0.88 ± 0.15 b
A0A034W273	Aspartate--tRNA ligase, cytoplasmic	539	49.91	25	↑ 3.07 ± 0.36	↑ 3.16 ± 0.72	1.07 ± 0.18 b
A0A034WMX6	Putative prefoldin subunit 5	160	76.88	10	↑ 3.07 ± 0.19	↑ 2.74 ± 0.15	0.91 ± 0.11 b
A0A034WJH2	Protein dpy-30-like protein	99	47.47	4	↑ 3.05 ± 0.22	↑ 3 ± 1.34	0.97 ± 0.13 b
A0A034VS83	Hypoxia up-regulated protein 1	957	42.11	30	↑ 2.93 ± 0.32	↑ 3.67 ± 0.83	1.28 ± 0.02 b
A0A034WCD3	NEDD8-activating enzyme E1 regulatory subunit	522	29.12	13	↑ 2.92 ± 0.5	↑ 4.43 ± 1.47	↑ 1.53 ± 0.03 a
A0A034VTV6	Glutaredoxin-related protein 5, mitochondrial	169	29.59	4	↑ 2.56 ± 0.2	↑ 2.67 ± 0.44	1.04 ± 0.1 b
A0A034W439	Ubiquitin carboxyl-terminal hydrolase	809	43.39	26	↑ 2.07 ± 0.2	↑ 3.26 ± 0.22	↑ 1.6 ± 0.05 a

A0A034WYF0	Peroxiredoxin-2	233	51.93	11	↑ 2.03 ± 0.05	↑ 2.43 ± 0.22	1.18 ± 0.17 b

Note: **a** means the fold change is ⩾ 1.5- or ⩽ 0.67-fold but at least one P-value ⩾ 0.05; **b** means no difference of protein abundance; " ↑ " represents protein abundance up-regulation; " ↓ " represents protein abundance down-regulation; Proteins with no functional annotation, and also sequence coverage ⩽ 20% were not listed in this table. Ov-1, ov-6 and ov-9 represent 1-, 6- and 9-day-old *B. dorsalis* ovary, respectively.

Table S5. Primers used for gene expression validation in developing ovaries of Bactrocera dorsalis.

Protein ID	Annotation	5′-Forward-3′	Reverse
A0A034WL03	Development-specific 25 kDa protein	GTCAAGGCGGTATTGTGATG	CCAACATACGATTGCTGACC
A0A034W585	Larval cuticle protein A3A	CATCAACACCGAGATTGACC	AGTGAACCATCAGCATCCAA
A0A034VWI9	General odorant-binding protein 99a	AGATCCGCAATCAGGATGAC	TCAGCCTTTACAGCGTCCTT
A0A034V8W7	Cytochrome P450 4g15	CTACAAGTTGGCCGATCGTT	CGTCACGTGGATCGTAAATG
A0A034VVP8	Serpin B9	GTACAGTGCCGGTCGATATG	CATGCGCTTCGACAAACTAT
M1F3Z9	Glutathione S-transferase omega-1	TCCCGAAGTACCACTCTTCC	CATGCCAGGTTTATCACCAC
A0A034UX56	Trehalose 6-phosphate phosphatase	GAGTATCCATCGGGCAAGAA	TTTCGATCAATTTGGCCTTC
A0A034VR92	Tropomyosin-2	AGGTCGTCGGTAACTCGTTG	CTTGTTAACGCCCAATTCGT
A0A034WDZ9	Importin-5	GGTACGCTTGATGGTCCCAA	GCTTCCGGTTCTGTGCCTAT
A0A034V813	Acetyl-CoA carboxylase	CAGTGTGCTTGCCCAATTCC	GGAACTGCGACTCCACTTCA
A0A034VA48	Prolyl 4-hydroxylase subunit a2	TACGCACAGAAGAGCACGAG	CCACCTTGTTCTACGTCGCT
A0A034V7N4	Myosin heavy chain 95F	CGTTGGCGTTACAAGCACAA	TGGGCGACACATCACTTTGA
A0A034VH20	Piwi-like protein	GGGGCCGTGCAAATGTATTC	AAACGAACCGGCATACCCTT
A0A034VER3	Eukaryotic translation initiation factor 3 subunit C	AAGTGACGCCGAAGAAGAGG	CGACTCAGATTCCGACTCCG
A0A034WHP3	Vitelline membrane protein Vm26Aa	AAGCTGTGAACCTCCAGTCG	TGAGCCGTATGATGGAGCAG
A0A034WWC1	General odorant-binding protein 99b	GGTACAAGCCGATGACTGGA	CGTCTTCCGGCATATCGTCT
Q8T7E3	Vitellogenin 1	GGAAGAACCCCAAACCCACA	GCACCAACACCTTGTCCAAC
A0A034W432	Cytochrome P450 306a1	GATTCCGATATTGCCGAAGA	CACTGCTCCAGATGTTCCAA
I1SWI8	Heat shock protein 60	CAATTGTTTGTCCGCAGCTA	TTGAACCAGTTTTGCACCAA
A0A034V6V9	Importin-4	GATGACCACGGCTACCCAAA	TGGTATTGGGTCGGTGATGC
A0A034WSR6	Defective chorion-1 protein	GCTGGCTTTATCGGTGTCCT	AAAGACTGGCCCACGATCTG
A0A034W812	Importin subunit alpha	GCGCAAATCAAAGAAAGAGG	ACGTTCGCGACTTAGCATTT
A0A034VZV8	Chorion peroxidase	CTGGTGGCACTTTCGGTTAT	TTATCATCAACGGCATTGGA

A0A034UZ33	Importin subunit beta	CAGGAAGACGCATTCCTAGC	AGTTGCTTTAGAGCCCGACA
A0A034V3F4	Tripeptidyl-peptidase 2	TGCCGCACATCCCAAGTATT	AAGGCGGTGCGTATTCTCAA
A0A034VV21	Nuclear pore complex protein Nup205	ACGAGGAAAGGCAGATGAAA	ACGTTGAATCGAGTGGTTCC
A0A034VMD8	Cytoskeleton-associated protein 5	GCGCTGCTAAATCTCATGGC	GCAGATCGGTGAATTTCGGC
A0A034W4A7	Glutathione S-transferase 1-1	CCCGAATTCCTGAAGATCAA	AATCGCCAAAGCAACCATAC
A0A034WP50	Chorion protein S36	AGTTGATCAGGGCGTCTACC	TTCAAGCTGAGTGGAGTTGG
A0A034VUE5	Myotubularin-related protein 14	TGGCCAGTACATTTGTCGAT	TCTGATCGATTCGCTGTAGG
A0A034W787	Juvenile hormone epoxide hydrolase 1	CACTAGACCCGGTTTCGATT	ATCCAACTGGCAATGTACGA
A0A034WCE7	Insulin receptor	AAGTAATACACCGCCGGAAC	TCGGTAATCTCCTCCACCTC
